# Supplementary material for: Determining molecular properties with differential mobility spectrometry and machine learning
Source: Nat Commun. 2018 Nov 30;9:5096. doi: 10.1038/s41467-018-07616-w (PMC6269546; doi:10.1038/s41467-018-07616-w)
Supplement: Supplementary file 1 — Supplementary Information [file 41467_2018_7616_MOESM1_ESM.docx]

Supplementary Information for

**Determining Molecular Properties with Differential Mobility Spectrometry and Machine Learning**

Stephen W. C. Walker,^1^ Ahdia Anwar,^1^ Jarrod M. Psutka,^1^ Jeff Crouse,^1^ Chang Liu,^2^ J. C. Yves Le Blanc,^2^ Justin Montgomery,^3^ Gilles H. Goetz,^3^ John S. Janiszewski,^3*^ J. Larry Campbell,^1,2*^ and W. Scott Hopkins ^1*^

**Affiliations:**

^1^ Department of Chemistry, University of Waterloo, Waterloo, ON, Canada, N2L 3G1.

^2^ SCIEX, 71 Four Valley Drive, Concord, ON, Canada, L4V 4V8.

^3^ Pfizer Global Research and Development, Eastern Point Road, Groton, CT, USA, 06340.

*Correspondence to: Scott.Hopkins@uwaterloo.ca

correspondence to: Scott.Hopkins@uwaterloo.ca

Supplementary Figures


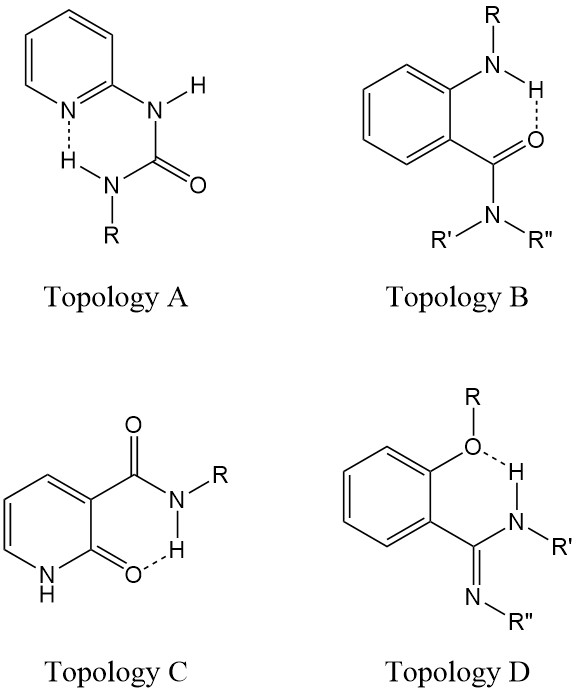


**Supplementary Figure 1.** General Structures for the four IMHB molecular topologies studied. (**A-D**) are given below. Red circles indicate the site(s) of protonation for the molecules, and the green regions highlight intramolecular hydrogen bonds.


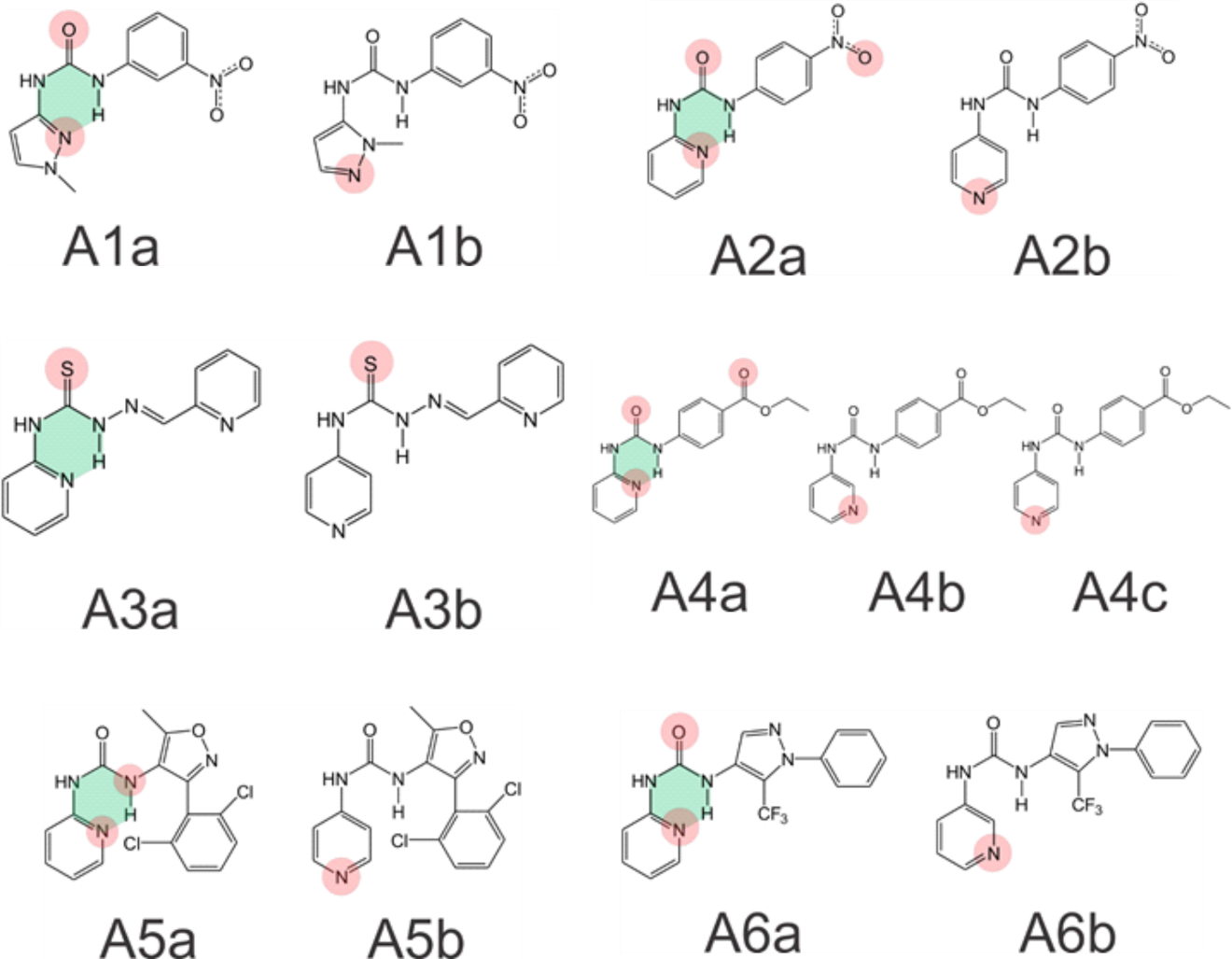


**Supplementary Figure 2.** Topology A IMHB molecules studied. Red circles indicate the site(s) of protonation for the molecules, and the green regions highlight intramolecular hydrogen bonds.


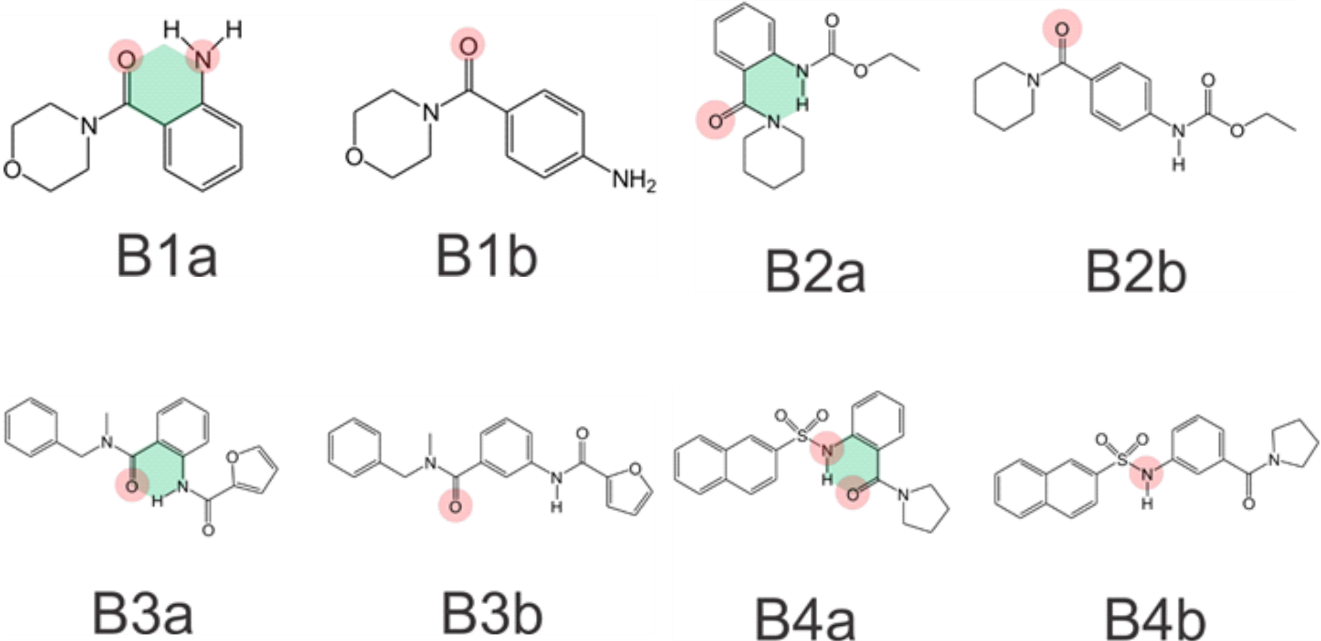


**Supplementary Figure 3.** Topology B IMHB molecules studied. Red circles indicate the site(s) of protonation for the molecules, and the green regions highlight intramolecular hydrogen bonds.


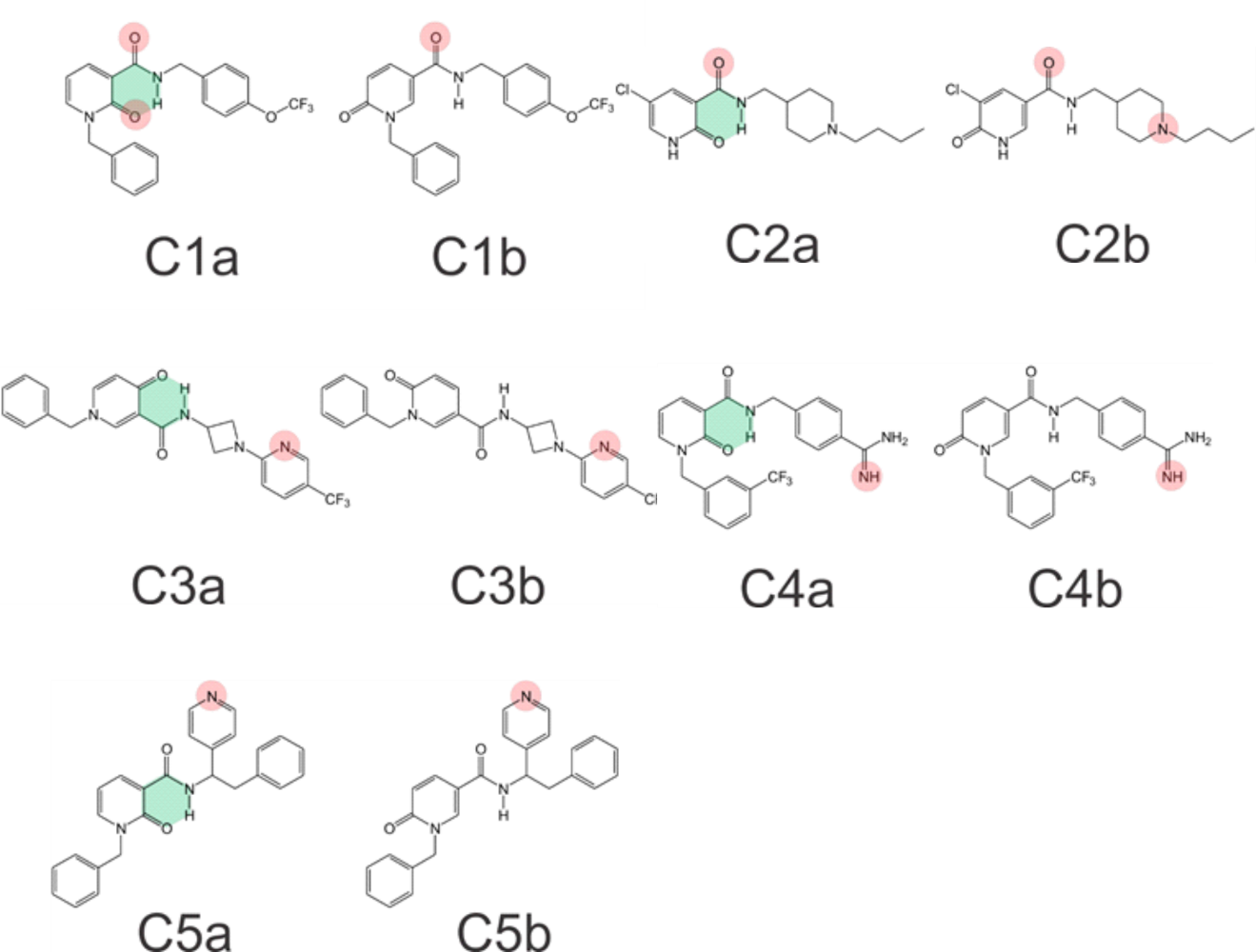


**Supplementary Figure 4.** Topology C IMHB molecules studied. Red circles indicate the site(s) of protonation for the molecules, and the green regions highlight intramolecular hydrogen bonds.


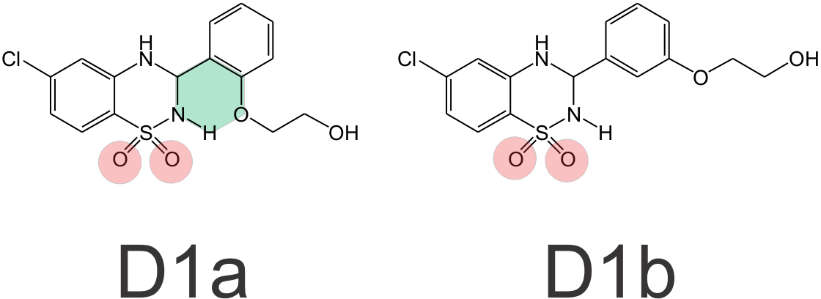


**Supplementary Figure 5.** Topology D IMHB molecules studied. Red circles indicate the site(s) of protonation for the molecules, and the green regions highlight intramolecular hydrogen bonds.


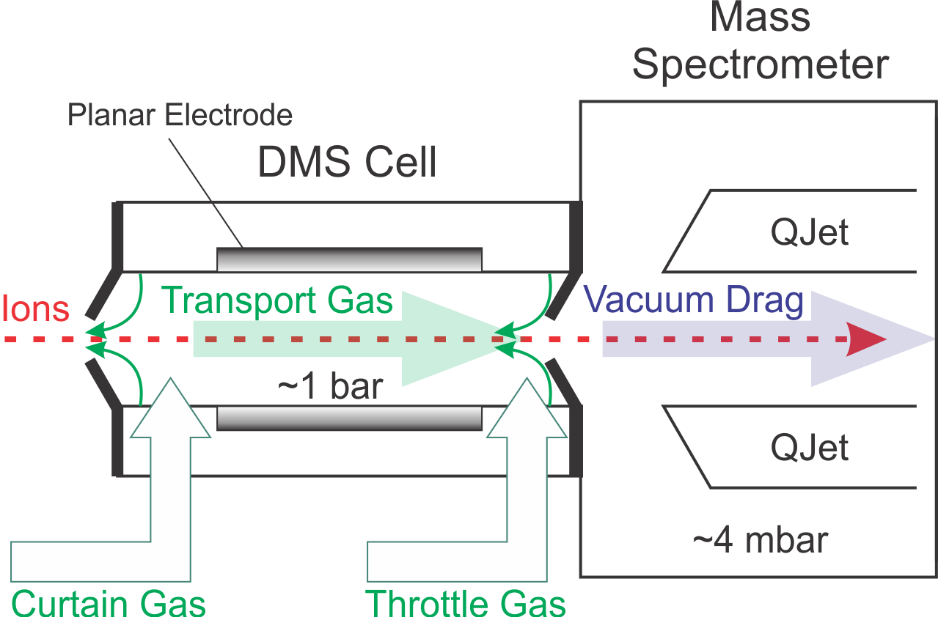


**Supplementary Figure 6.** Cross-sectional view of the Differential Mobility Spectrometry – Mass Spectrometry source. Volatile chemical modifiers can be added to the curtain gas or throttle gas. Deuterated solvent vapor can be added to the throttle gas to perform HDX experiments on the DMS-separated species.


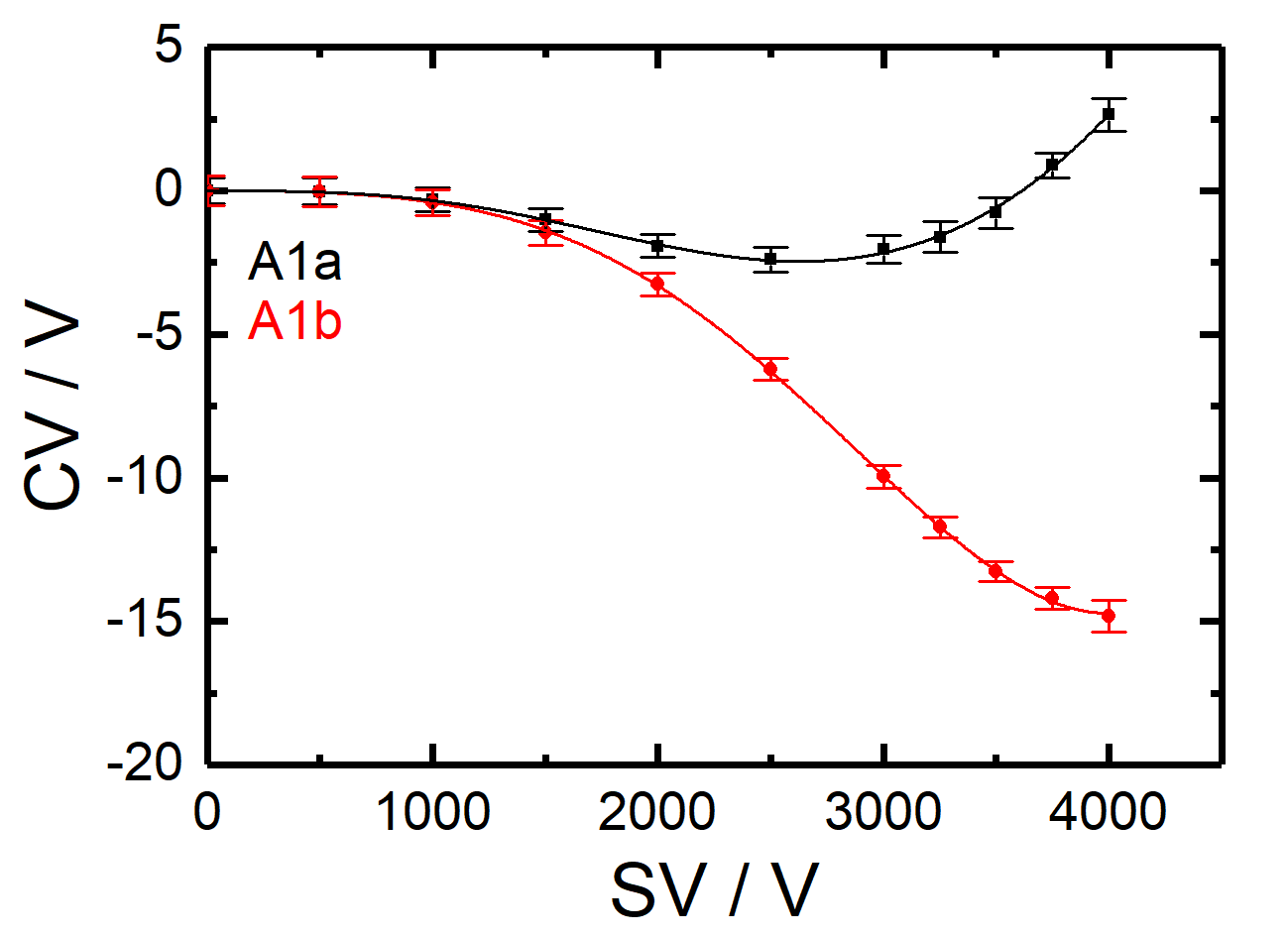


**Supplementary Figure 7.** DMS dispersion plots of molecule set **A1**. All data is recorded in an N_2_ environment seeded with methanol vapor (1.5% v/v; *ca*. 13 mbar) at 300 °C. Molecule **A1a** (black trace) exhibits an IMHB whereas molecule **A1b** (red trace) does not.


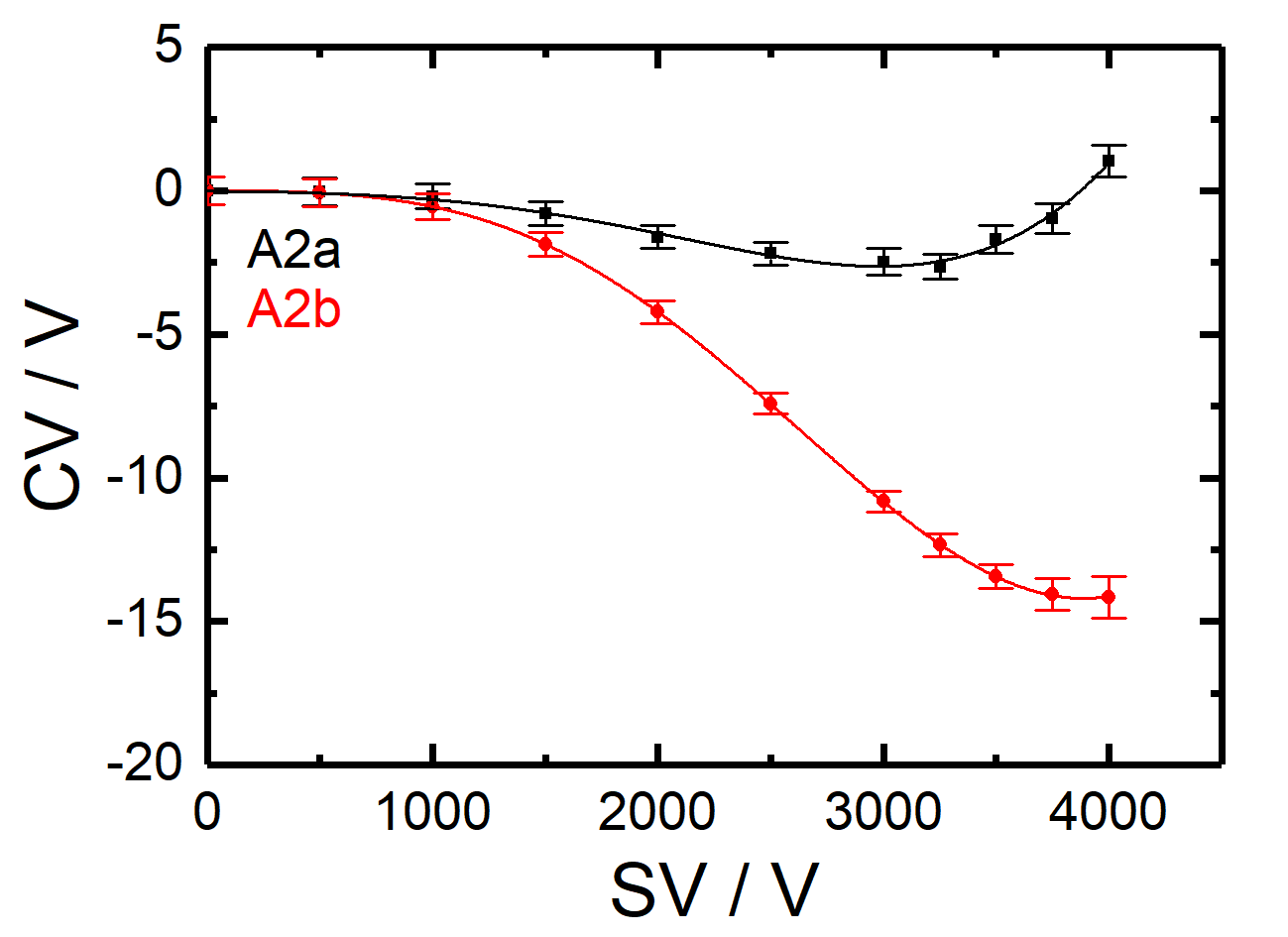


**Supplementary Figure 8.** DMS dispersion plots of molecule set **A2**. All data is recorded in an N_2_ environment seeded with methanol vapor (1.5% v/v; *ca*. 13 mbar) at 300 °C. Molecule **A2a** (black trace) exhibits an IMHB whereas molecule **A2b** (red trace) does not.


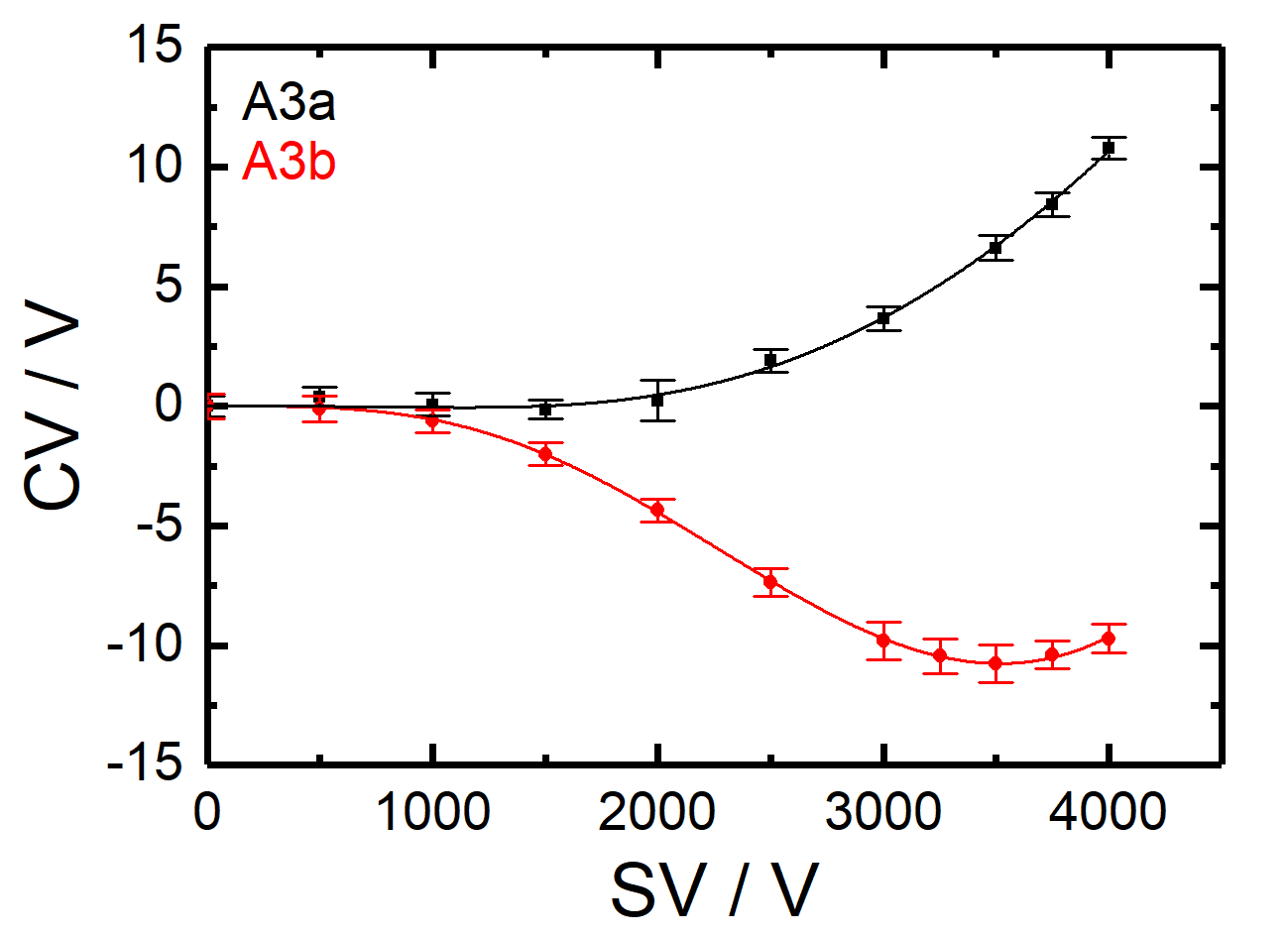


**Supplementary Figure 9.** DMS dispersion plots of molecule set **A3**. All data is recorded in an N_2_ environment seeded with methanol vapor (1.5% v/v; *ca*. 13 mbar) at 300 °C. Molecule **A3a** (black trace) exhibits an IMHB whereas molecule **A3b** (red trace) does not.


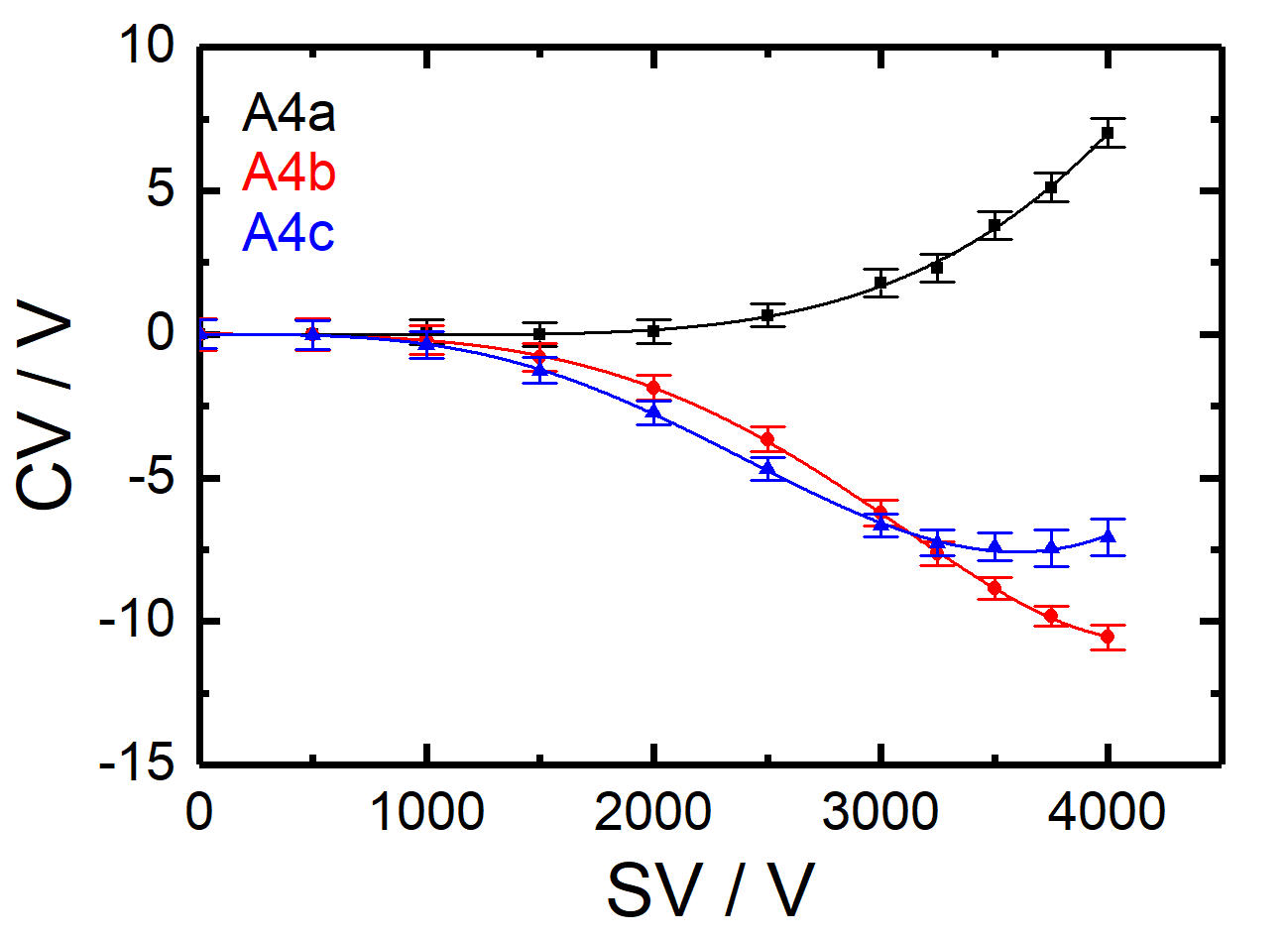


**Supplementary Figure 10.** DMS dispersion plots of molecule set **A4**. All data is recorded in an N_2_ environment seeded with methanol vapor (1.5% v/v; *ca*. 13 mbar) at 300 °C. Molecule **A4a** (black trace) exhibits an IMHB whereas molecules **A4b** (red trace) and **A4c** (blue trace) do not.


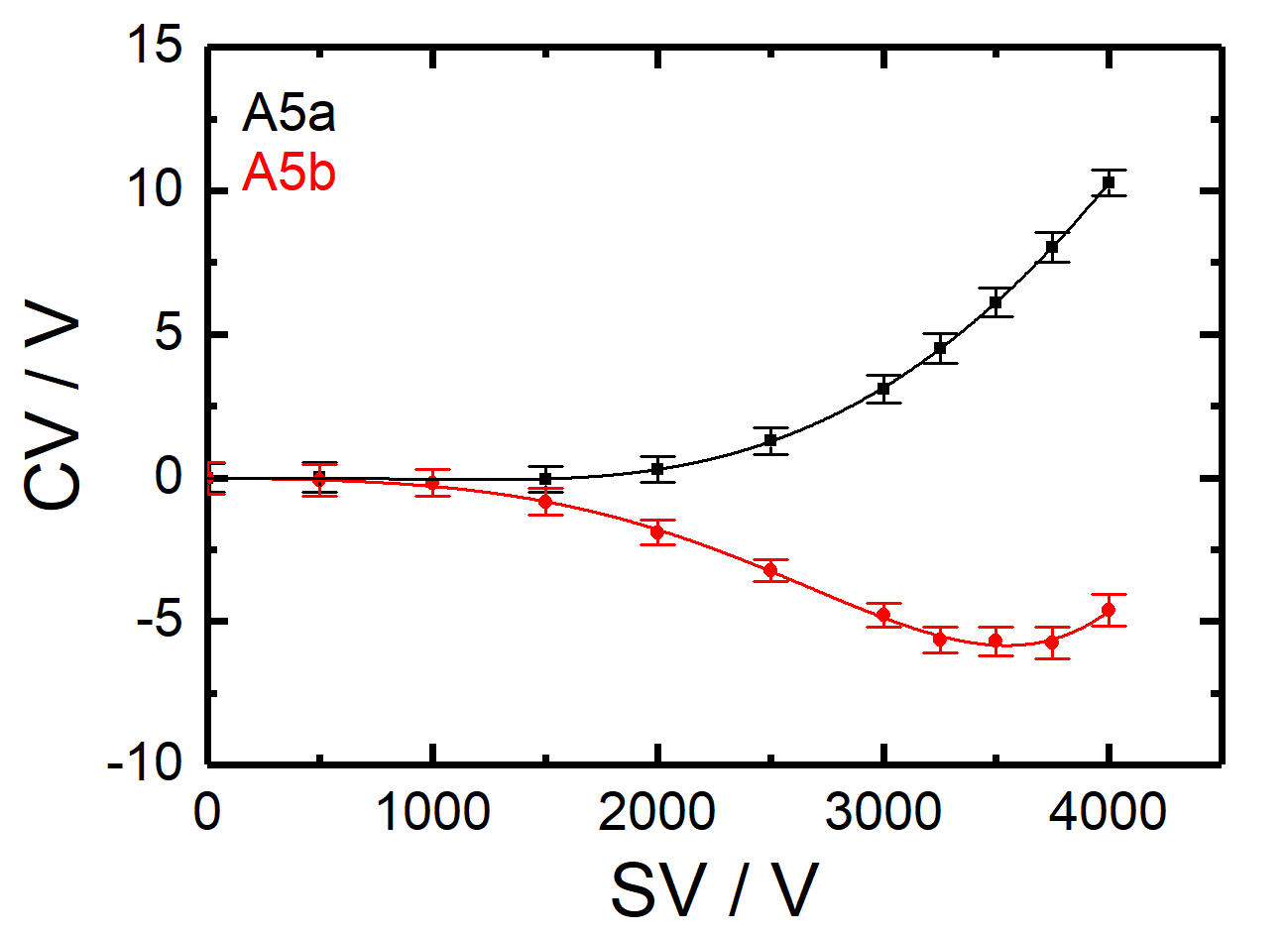


**Supplementary Figure 11.** DMS dispersion plots of molecule set **A5**. All data is recorded in an N_2_ environment seeded with methanol vapor (1.5% v/v; *ca*. 13 mbar) at 300 °C. Molecule **A5a** (black trace) exhibits an IMHB whereas molecule **A5b** (red trace) does not.


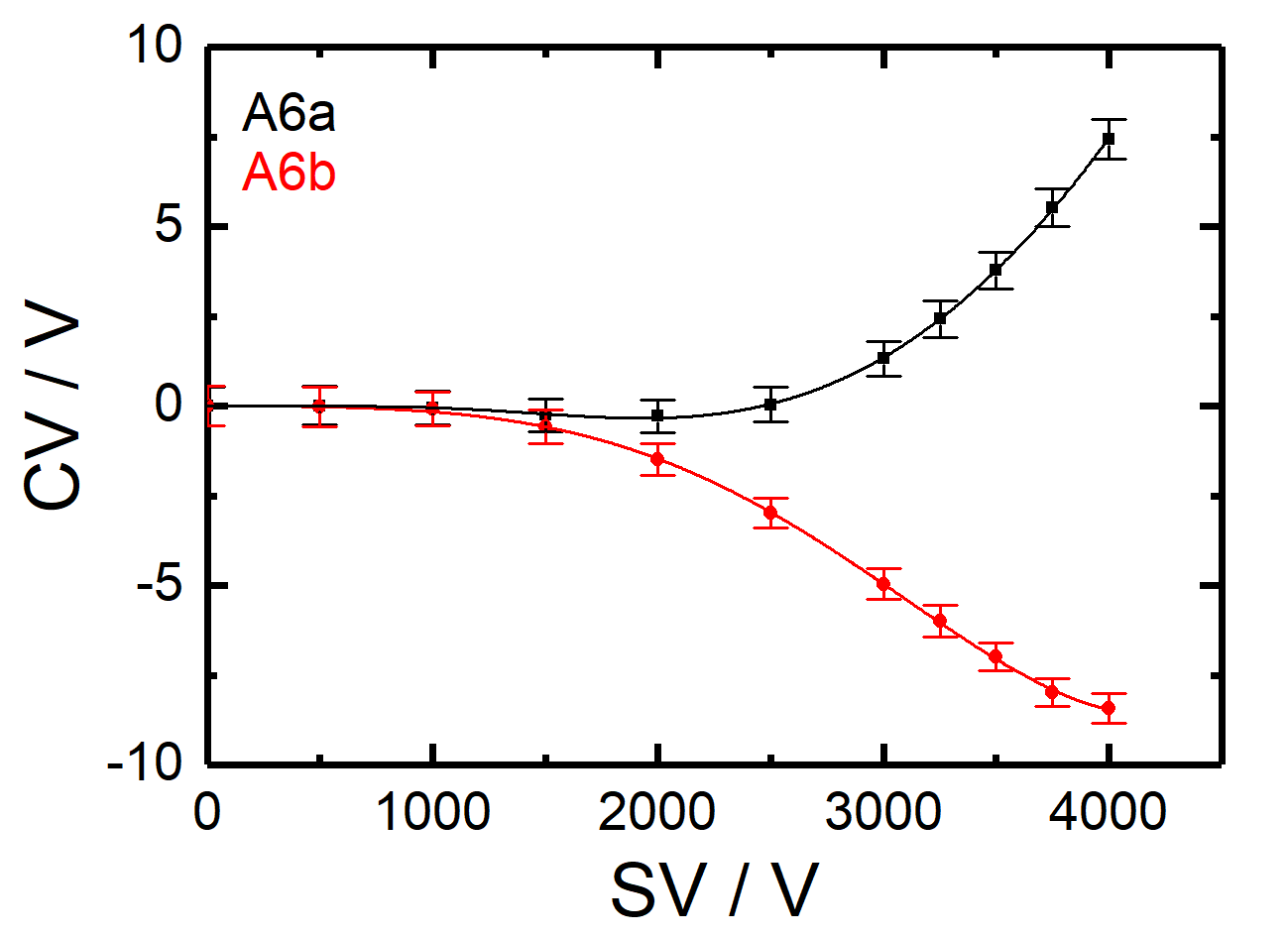


**Supplementary Figure 12.** DMS dispersion plots of molecule set **A6**. All data is recorded in an N_2_ environment seeded with methanol vapor (1.5% v/v; *ca*. 13 mbar) at 300 °C. Molecule **A6a** (black trace) exhibits an IMHB whereas molecule **A6b** (red trace) does not.


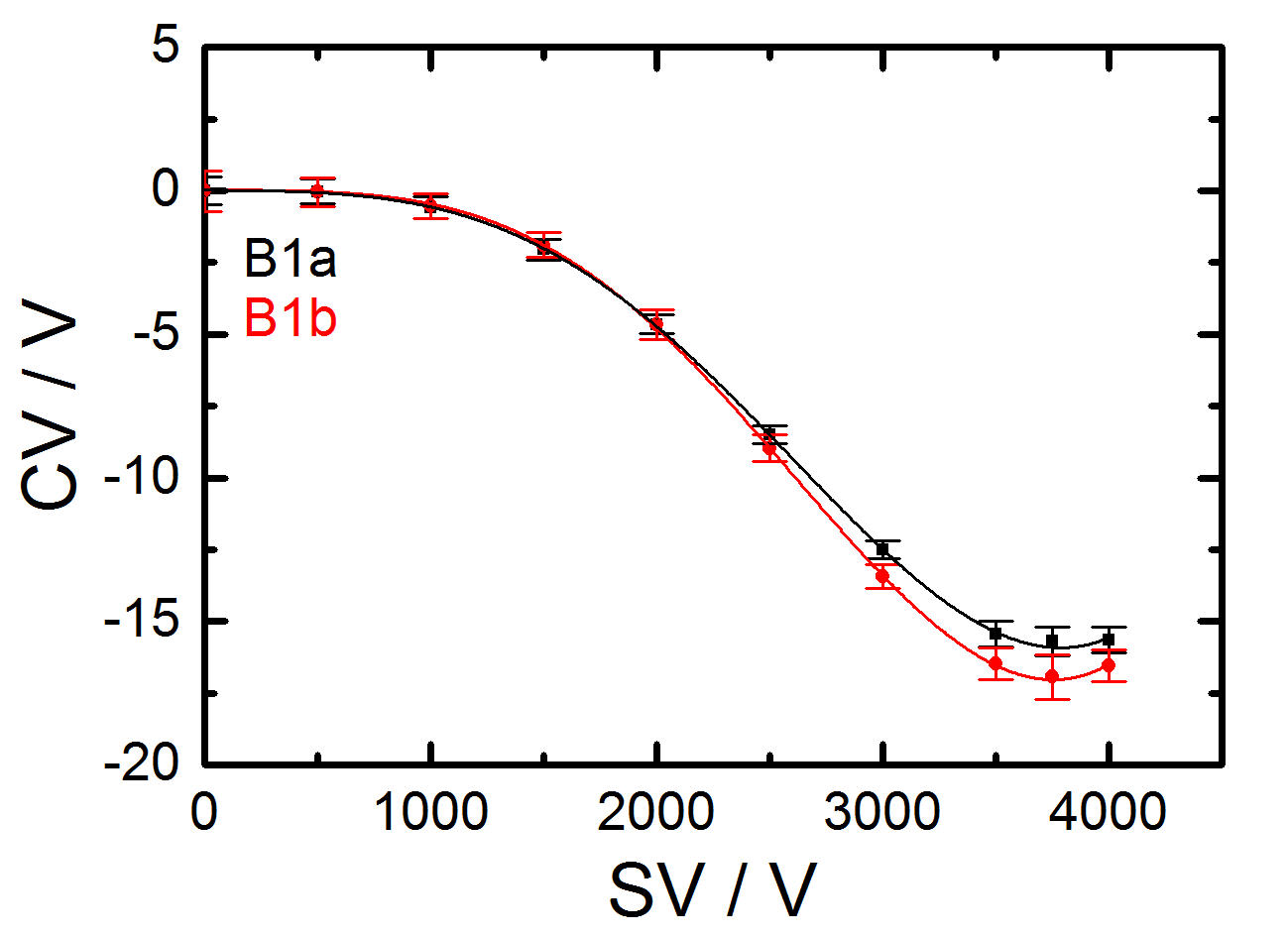


**Supplementary Figure 13.** DMS dispersion plots of molecule set **B1**. All data is recorded in an N_2_ environment seeded with methanol vapor (1.5% v/v; *ca*. 13 mbar) at 300 °C. Molecule **B1a** (black trace) exhibits an IMHB whereas molecule **B1b** (red trace) does not.


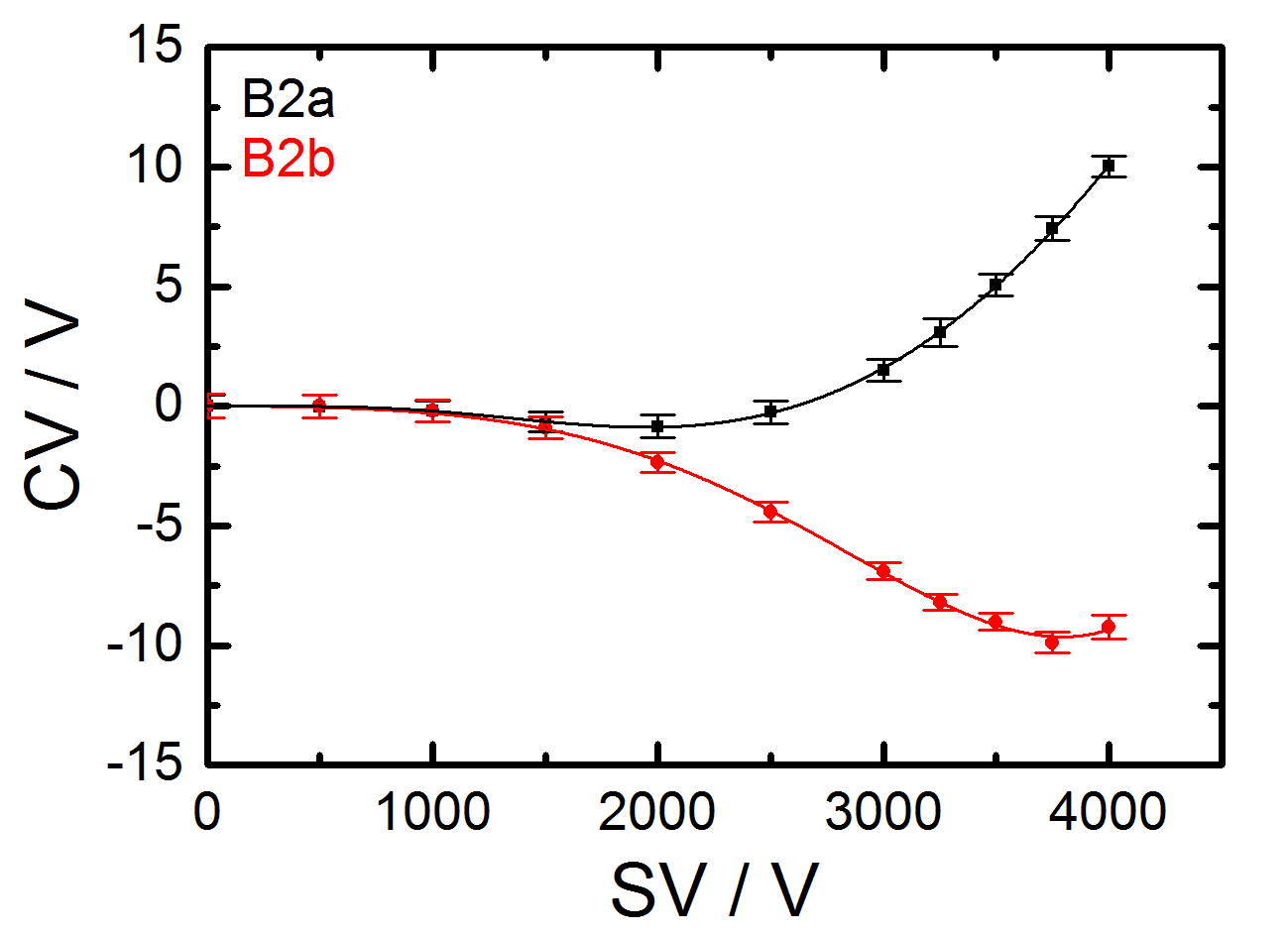


**Supplementary Figure 14.** DMS dispersion plots of molecule set **B2**. All data is recorded in an N_2_ environment seeded with methanol vapor (1.5% v/v; *ca*. 13 mbar) at 300 °C. Molecule **B2a** (black trace) exhibits an IMHB whereas molecule **B2b** (red trace) does not.

**
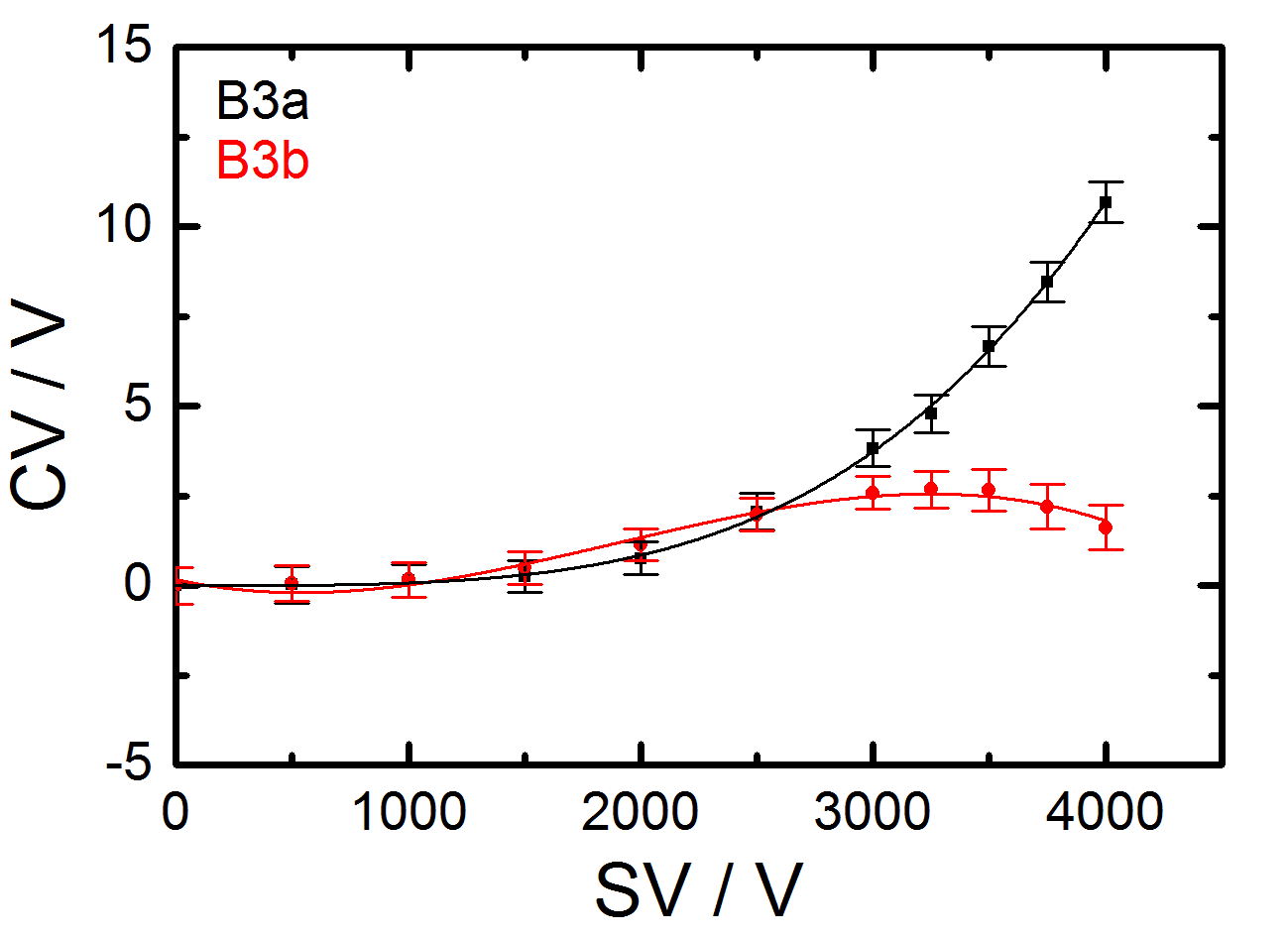
**

**Supplementary Figure 15.** DMS dispersion plots of molecule set **B3**. All data is recorded in an N_2_ environment seeded with methanol vapor (1.5% v/v; *ca*. 13 mbar) at 300 °C. Molecule **B3a** (black trace) exhibits an IMHB whereas molecule **B3b** (red trace) does not. In the case of **B3b** we observe a more exotic clustering behavior, suggesting dissociation of a strongly bound ion-solvent cluster at *ca*. SV = 3000 V.


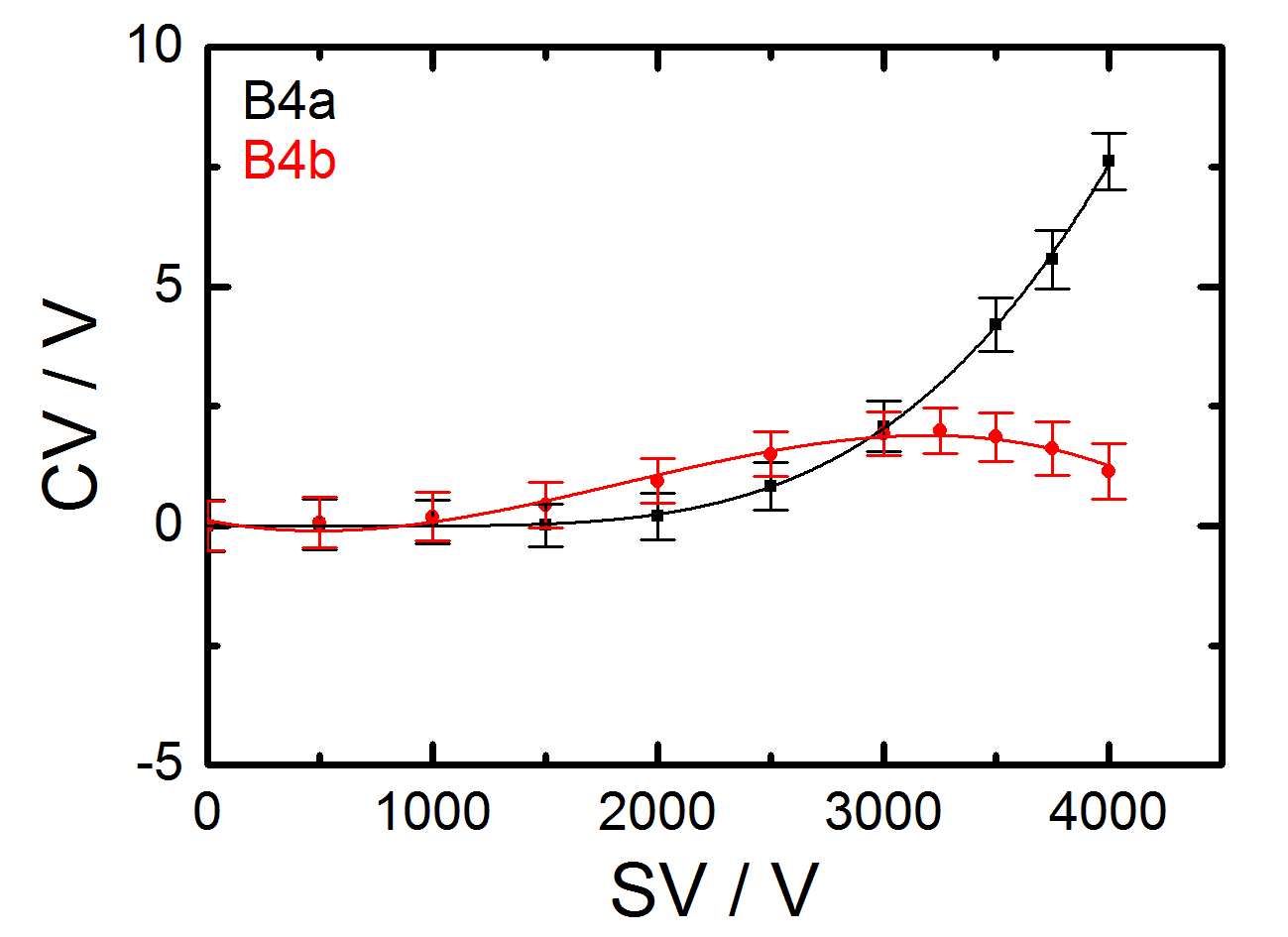


**Supplementary Figure 16.** DMS dispersion plots of molecule set **B4**. All data is recorded in an N_2_ environment seeded with methanol vapor (1.5% v/v; *ca*. 13 mbar) at 300 °C. Molecule **B4a** (black trace) exhibits an IMHB whereas molecule **B4b** (red trace) does not. the case of **B4b** we observe a more exotic clustering behavior, suggesting dissociation of a strongly bound ion-solvent cluster at *ca*. SV = 3000 V


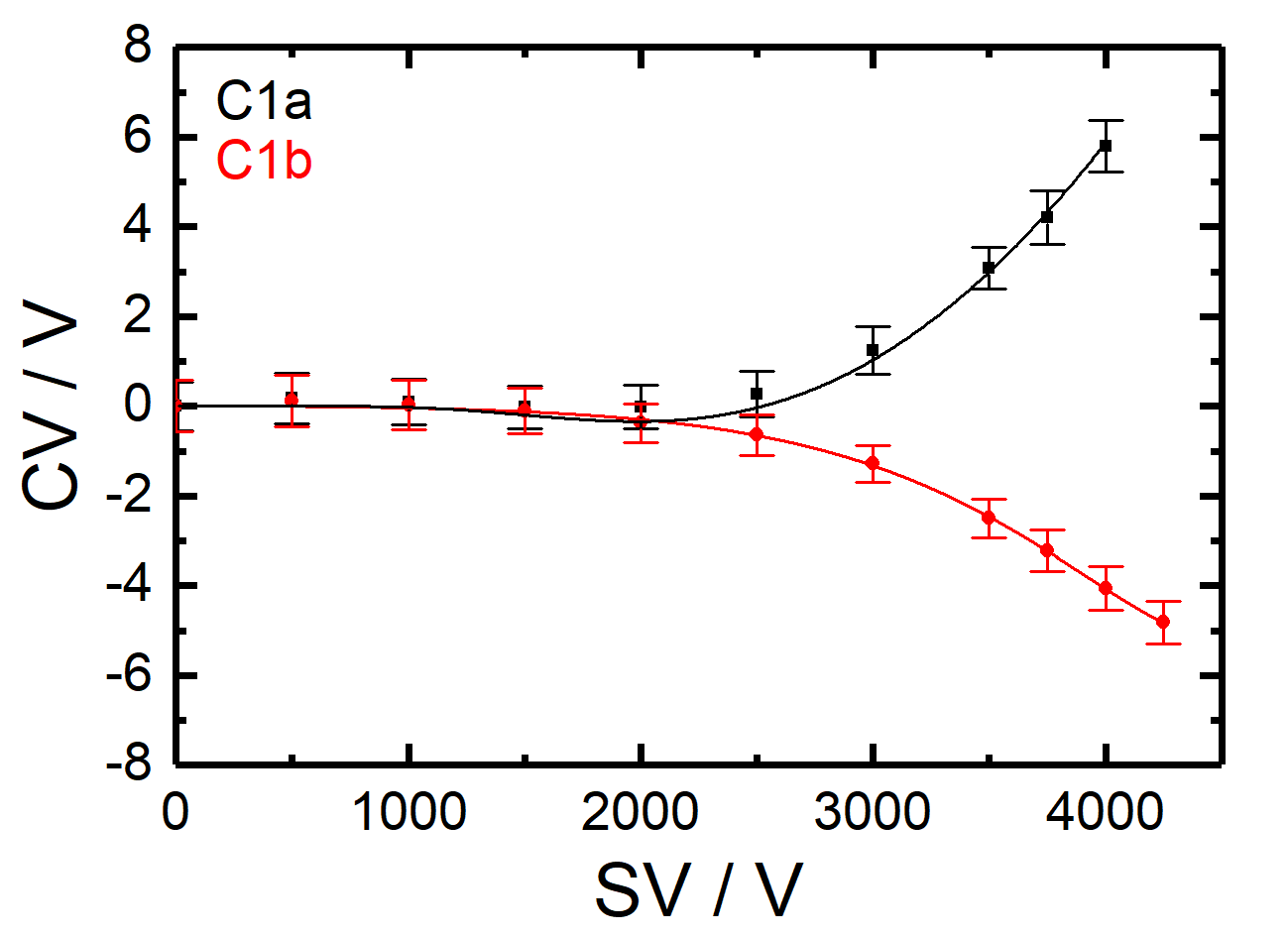


**Supplementary Figure 17.** DMS dispersion plots of molecule set **C1**. All data is recorded in a N_2_ environment seeded with methanol vapor (1.5% v/v; *ca*. 13 mbar) at 300 °C. Molecule **C1a** (black trace) exhibits an IMHB whereas molecule **C1b** (red trace) does not.


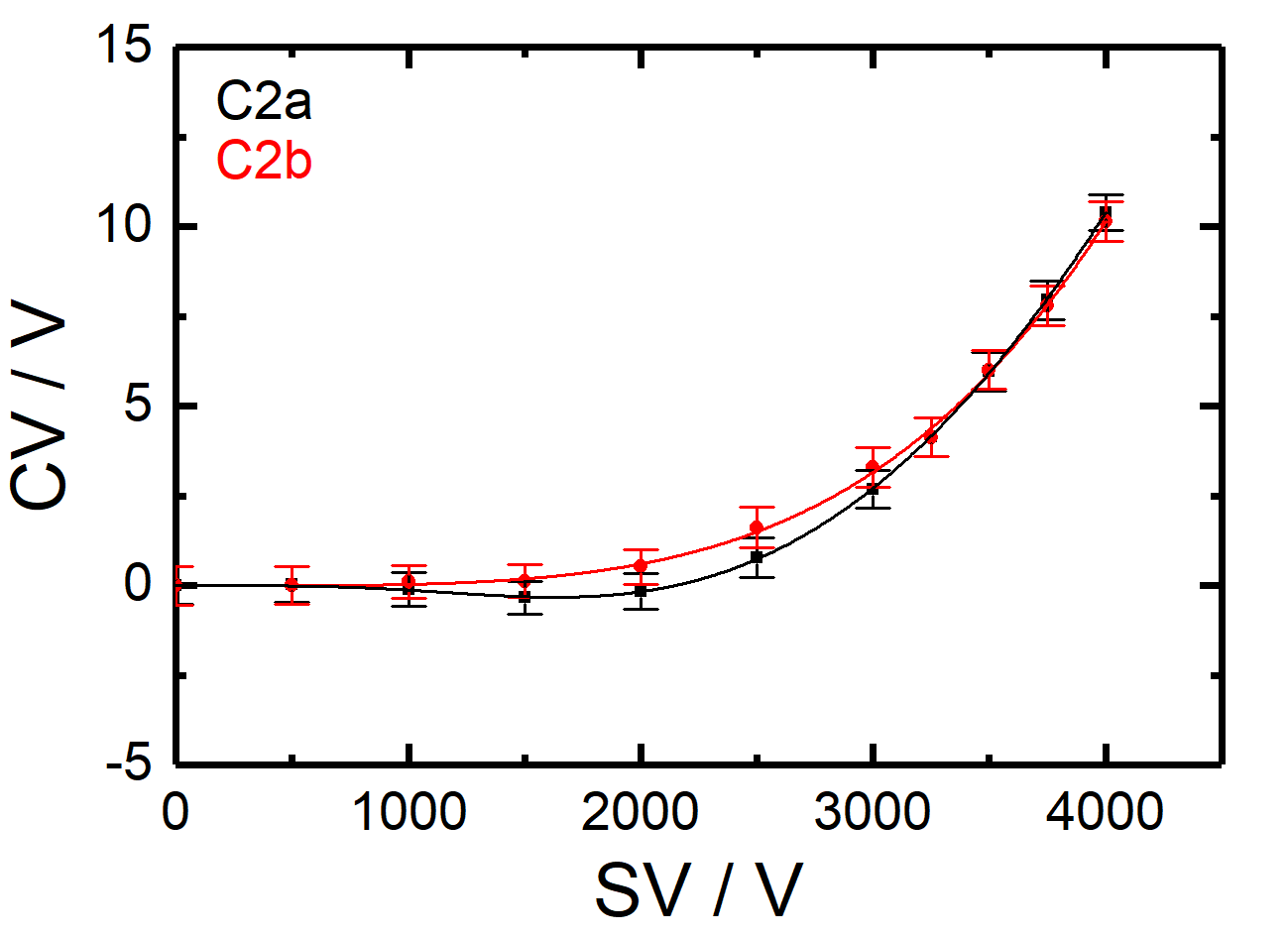


**Supplementary Figure 18.** DMS dispersion plots of molecule set **C2**. DMS dispersion plots of molecule set **C2** recorded in a N_2_ environment seeded with methanol vapor (1.5% v/v; *ca*. 13 mbar) at 300 °C. Molecule **C2a** (black trace) exhibits an IMHB whereas molecule **C2b** (red trace) does not.


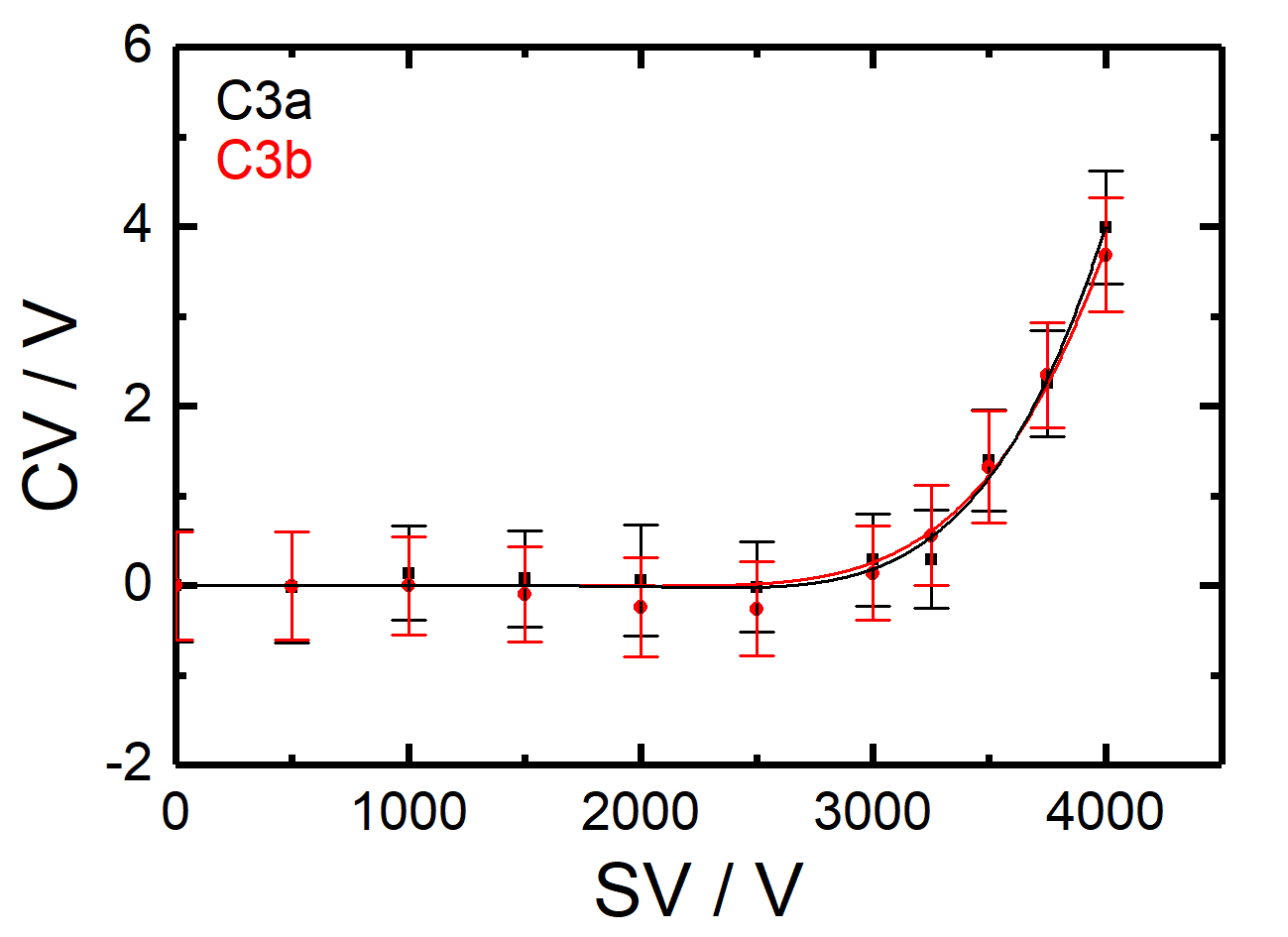


**Supplementary Figure 19.** DMS dispersion plots of molecule set C3. All data is recorded in an N_2_ environment seeded with methanol vapor (1.5% v/v; *ca*. 13 mbar) at 300 °C. Molecule **C3a** (black trace) exhibits an IMHB whereas molecule **C3b** (red trace) does not.


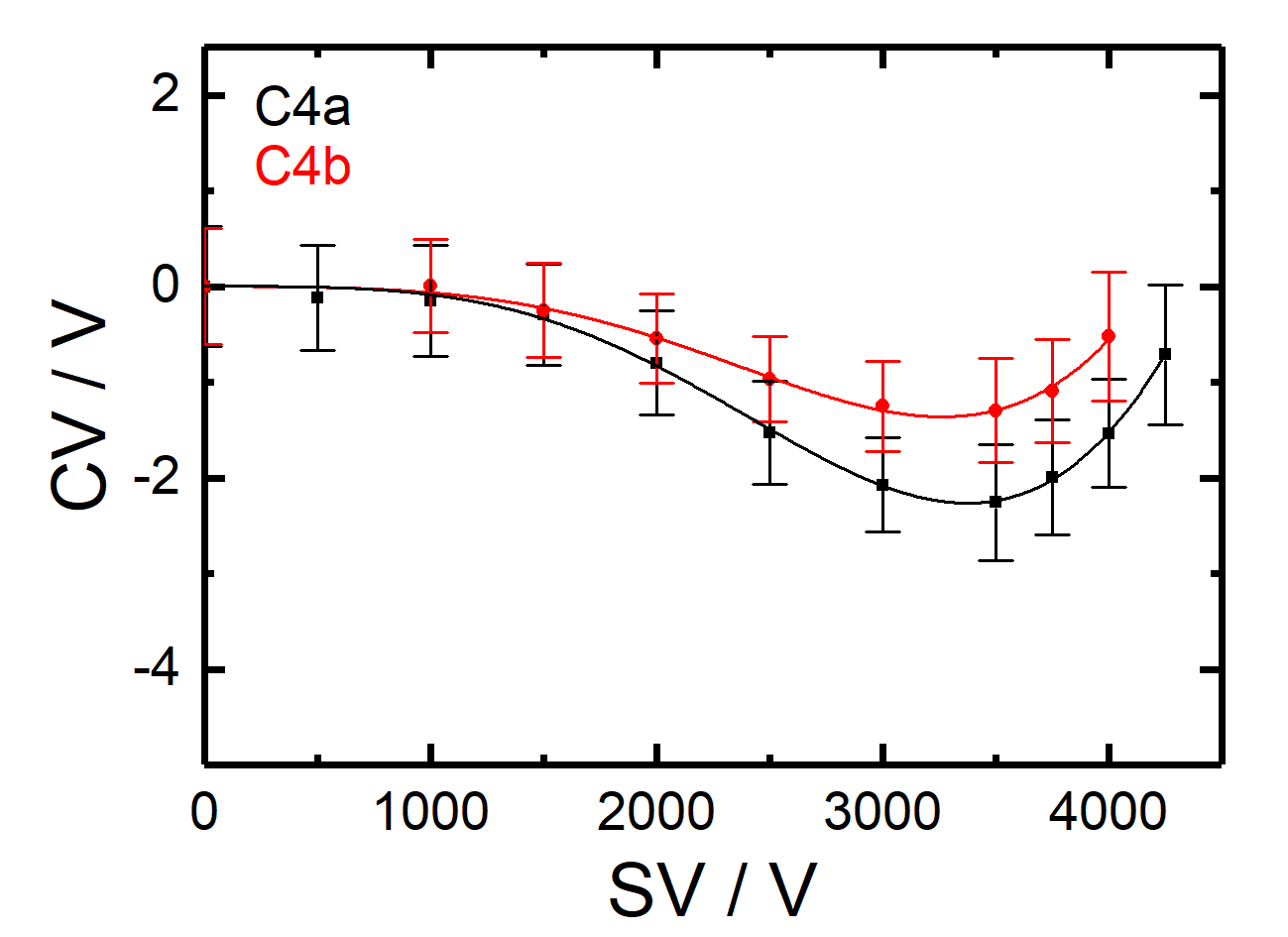


**Supplementary Figure 20.** DMS dispersion plots of molecule set C4. All data is recorded in an N_2_ environment seeded with methanol vapor (1.5% v/v; *ca*. 13 mbar) at 300 °C. Molecule **C4a** (black trace) exhibits an IMHB whereas molecule **C4b** (red trace) does not.


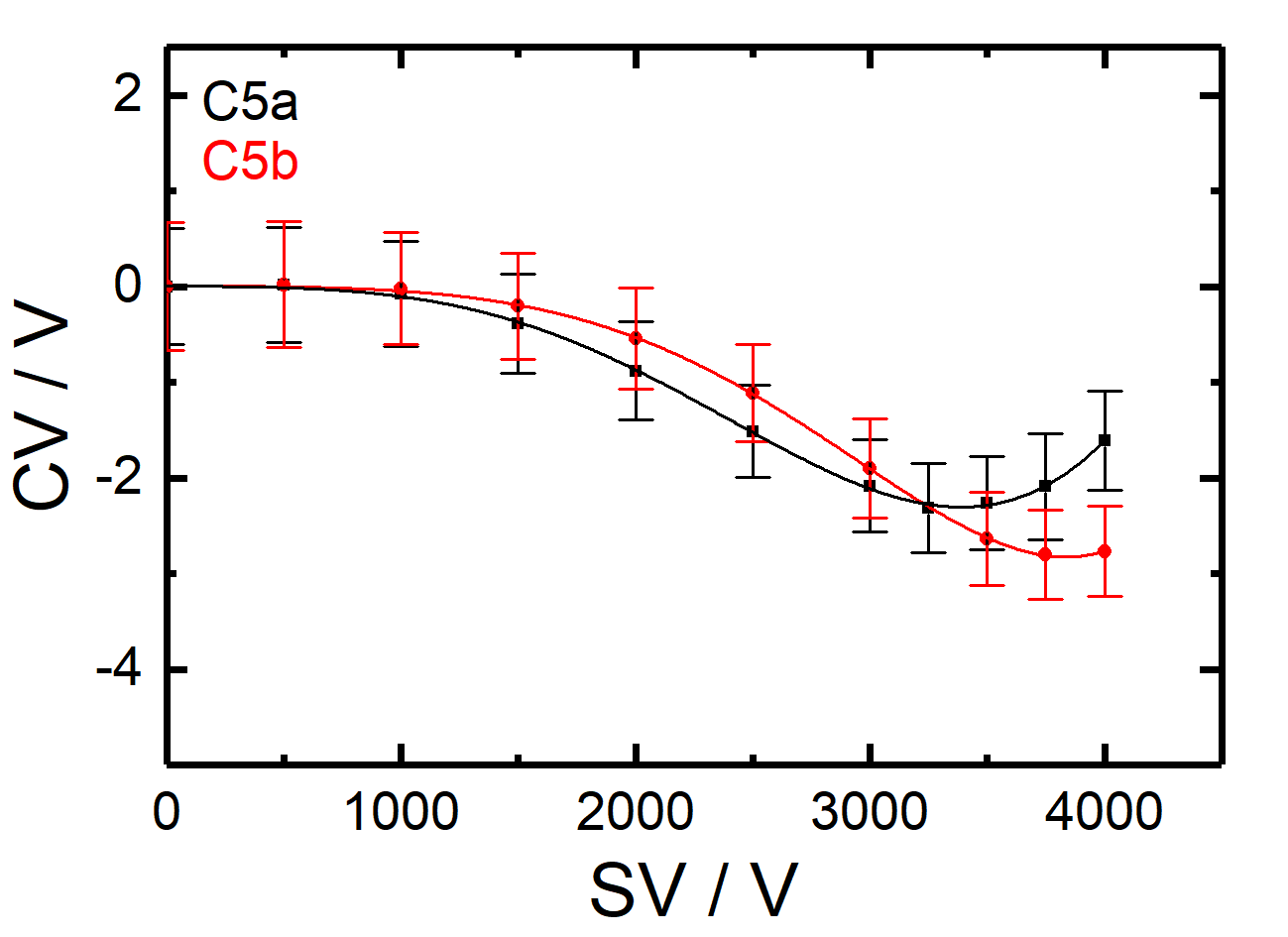


**Supplementary Figure 21.** DMS dispersion plots of molecule set C5. DMS dispersion plots of molecule set **C5** recorded in a N_2_ environment seeded with methanol vapor (1.5% v/v; *ca*. 13 mbar) at 300 °C. Molecule **C5a** (black trace) exhibits an IMHB whereas molecule **C5b** (red trace) does not.


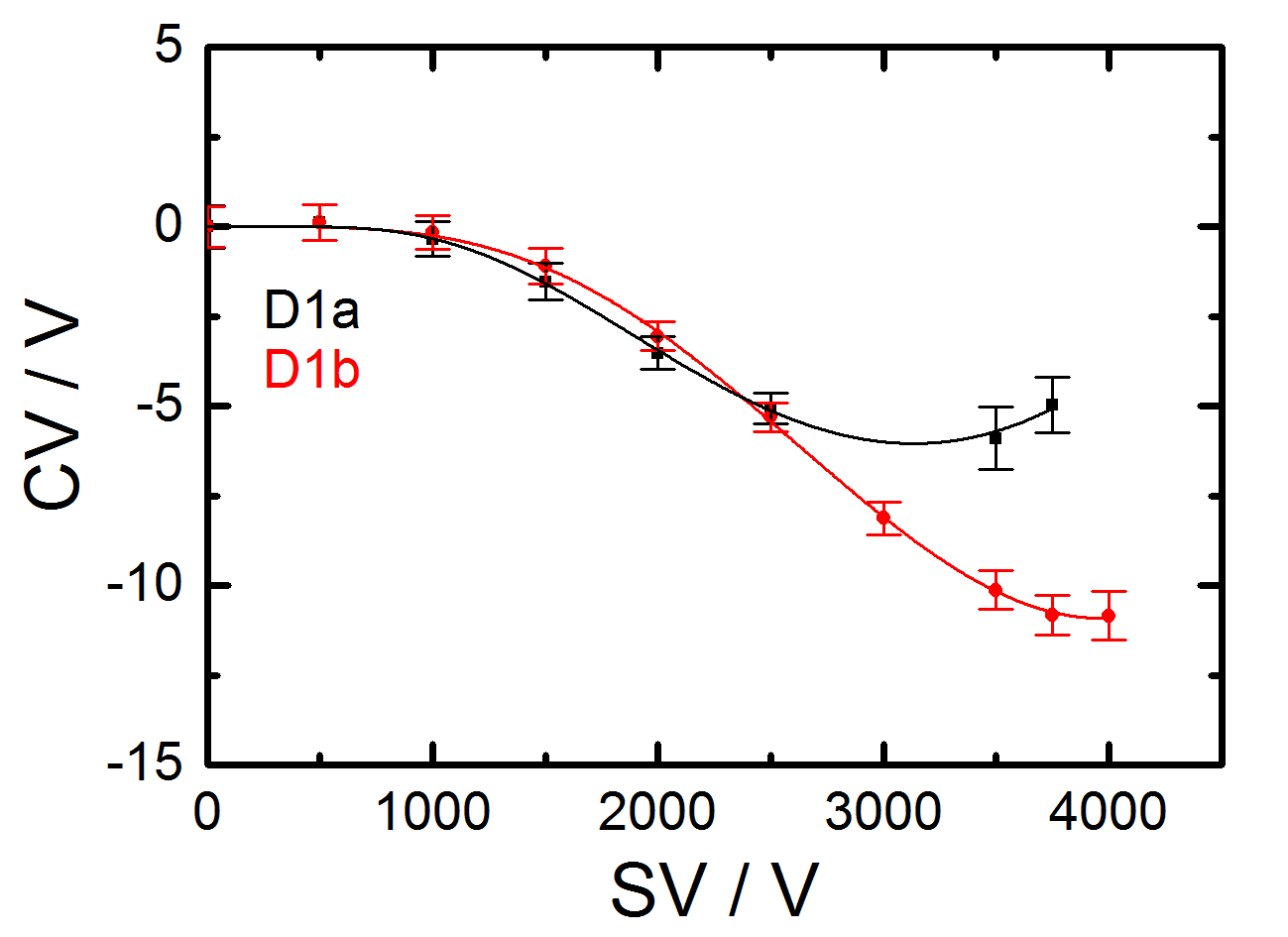


**Supplementary Figure 22.** DMS dispersion plots of molecule set D1. All data is recorded in an N_2_ environment seeded with methanol vapor (1.5% v/v; *ca*. 13 mbar) at 300 °C. Molecule **D1a** (black trace) exhibits an IMHB whereas molecule **D1b** (red trace) does not.


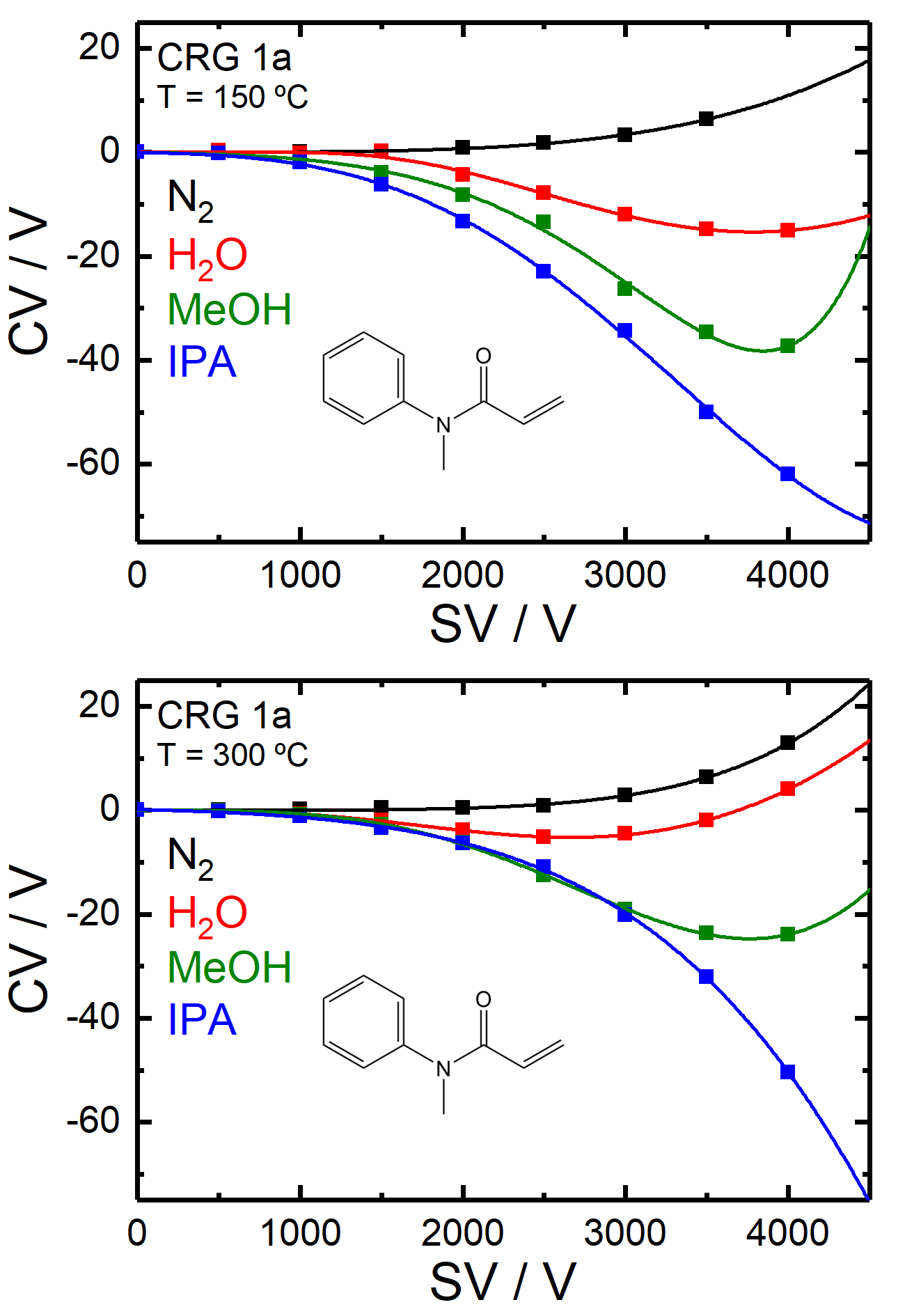


**Supplementary Figure 23.** Dispersion plots for CRG 1a. All data is acquired in (black) a pure N_2_ environment, and N_2_ environments seeded with 1.5 % (v/v) (red) water, (green) methanol, and (blue) isopropanol vapor at temperatures of 150 °C and 300 °C.

**
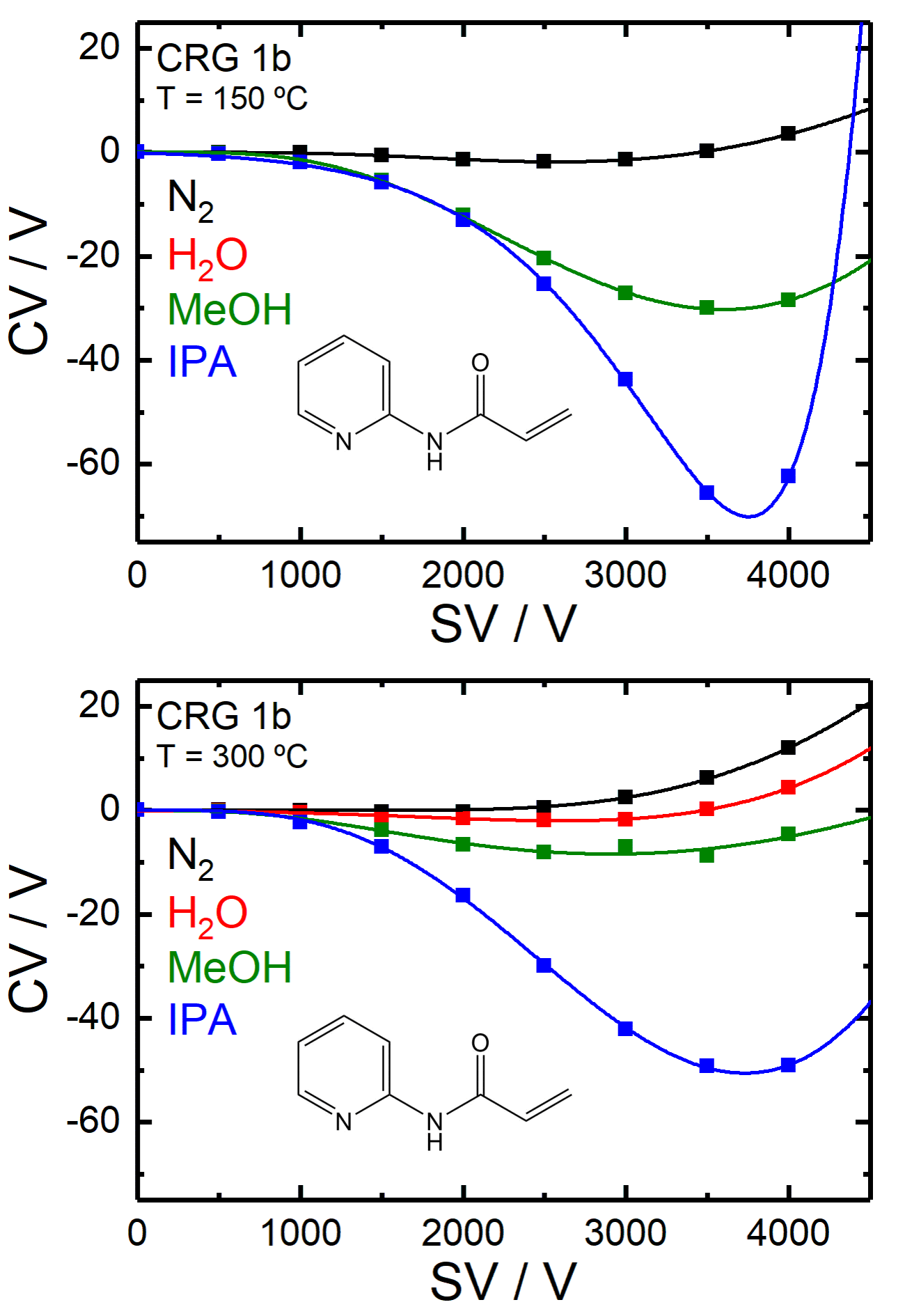
**

**Supplementary Figure 24.** Dispersion plots for CRG 1b. All data acquired in (black) a pure N_2_ environment, and N_2_ environments seeded with 1.5 % (v/v) (red) water, (green) methanol, and (blue) isopropanol vapor at temperatures of 150 °C and 300 °C. The ion signal for the T = 150 °C water-modified experiment was too low for accurate dispersion plot measurement.


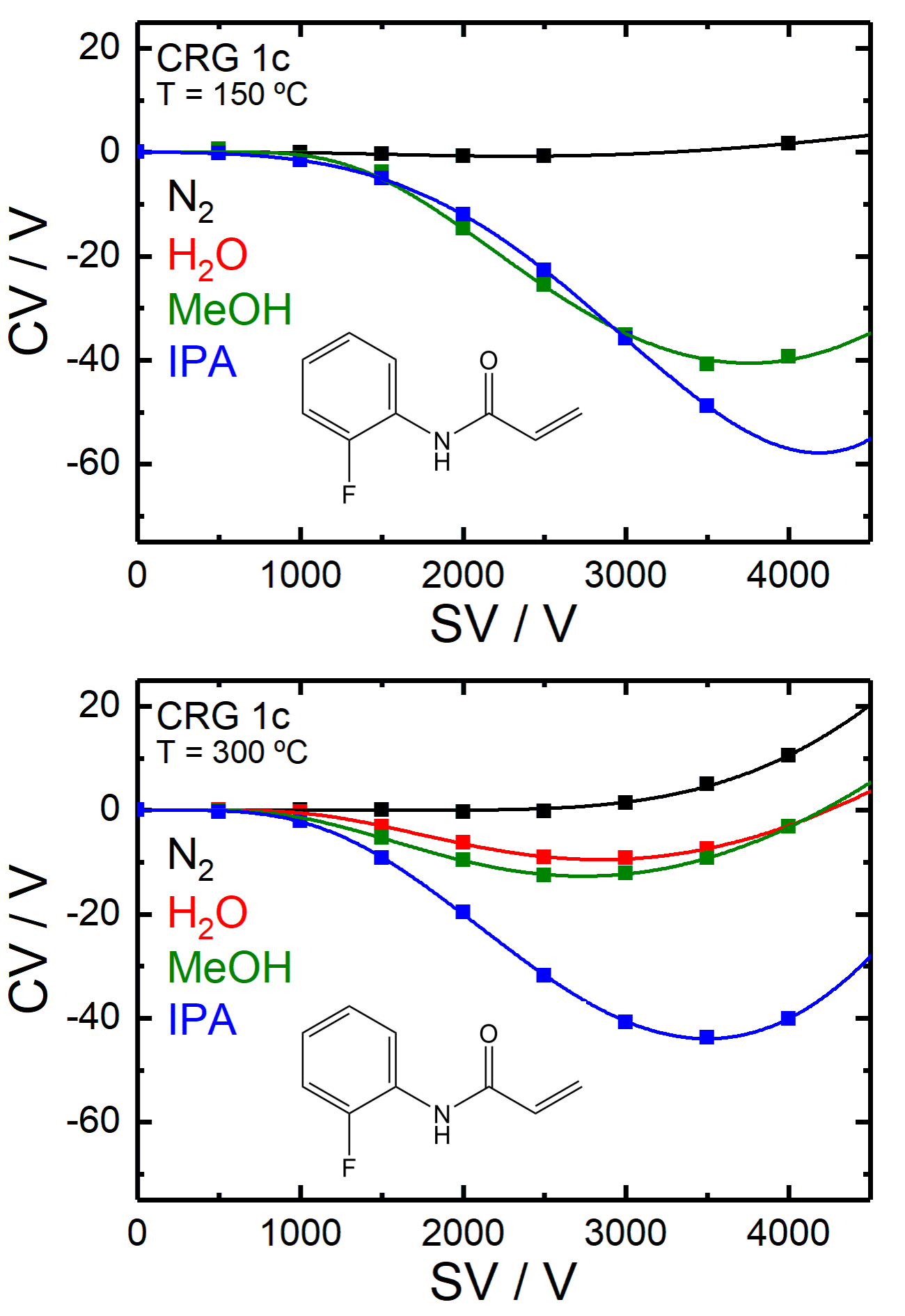


**Supplementary Figure 25.** Dispersion plots for CRG 1c. All data acquired in (black) a pure N_2_ environment, and N_2_ environments seeded with 1.5 % (v/v) (red) water, (green) methanol, and (blue) isopropanol vapor at temperatures of 150 °C and 300 °C. The ion signal for the T = 150 °C water-modified experiment was too low for accurate dispersion plot measurement.


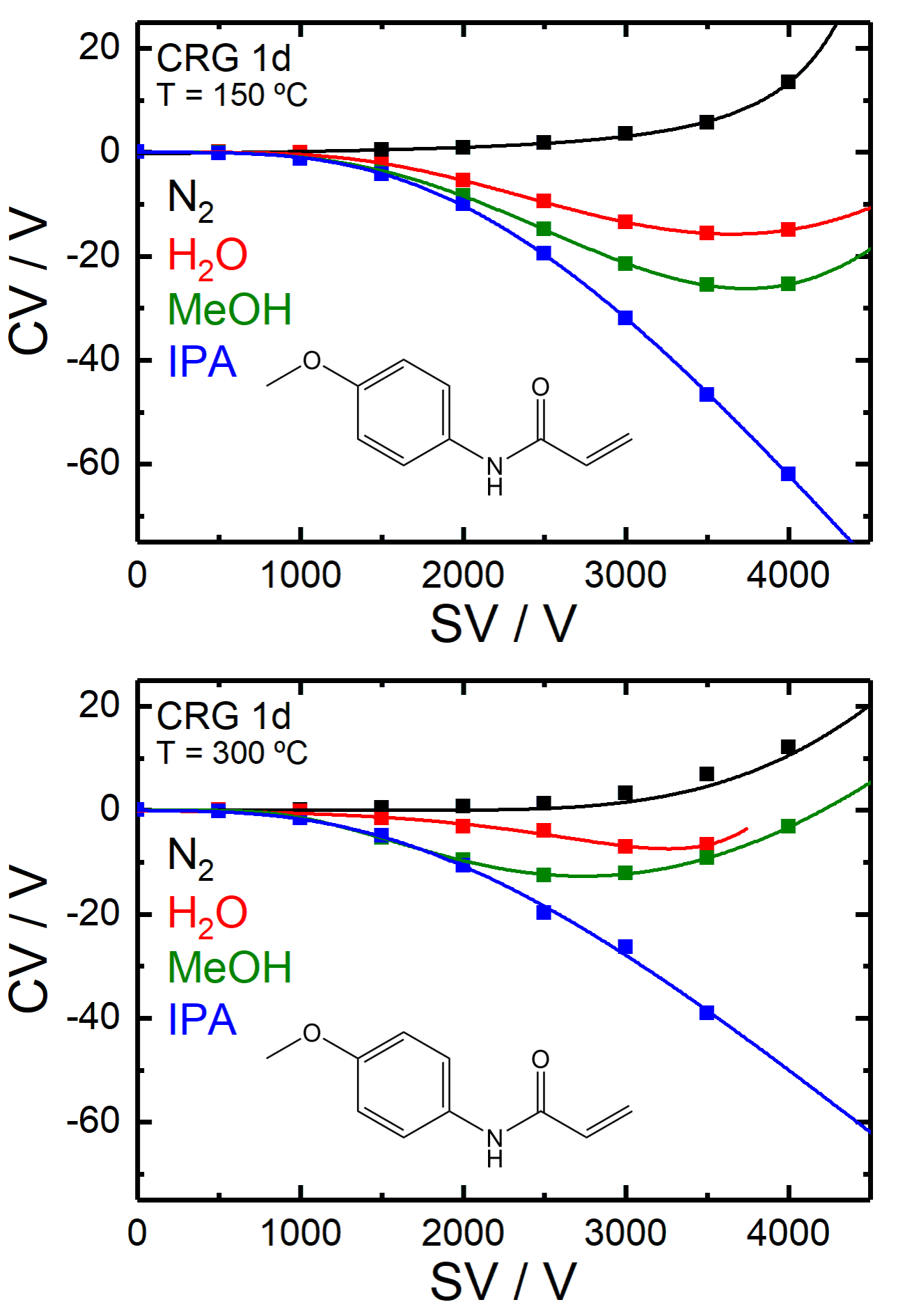


**Supplementary Figure 26.** Dispersion plots for CRG 1d. All data acquired in (black) a pure N_2_ environment, and N_2_ environments seeded with 1.5 % (v/v) (red) water, (green) methanol, and (blue) isopropanol vapor at temperatures of 150 °C and 300 °C.


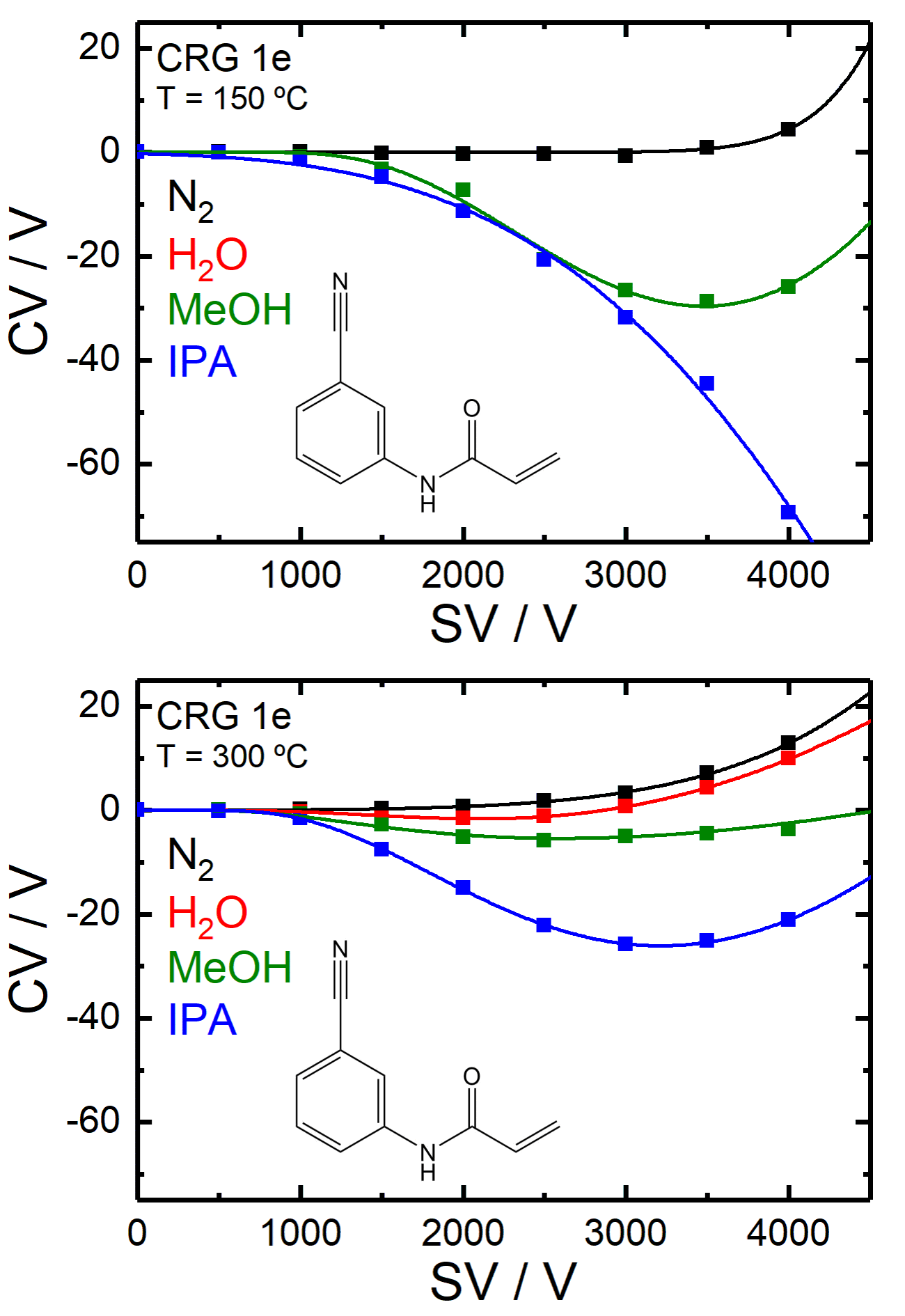


**Supplementary Figure 27.** Dispersion plots for CRG 1e. All data acquired in (black) a pure N_2_ environment, and N_2_ environments seeded with 1.5 % (v/v) (red) water, (green) methanol, and (blue) isopropanol vapor at temperatures of 150 °C and 300 °C. The ion signal for the T = 150 °C water-modified experiment was too low for accurate dispersion plot measurement.


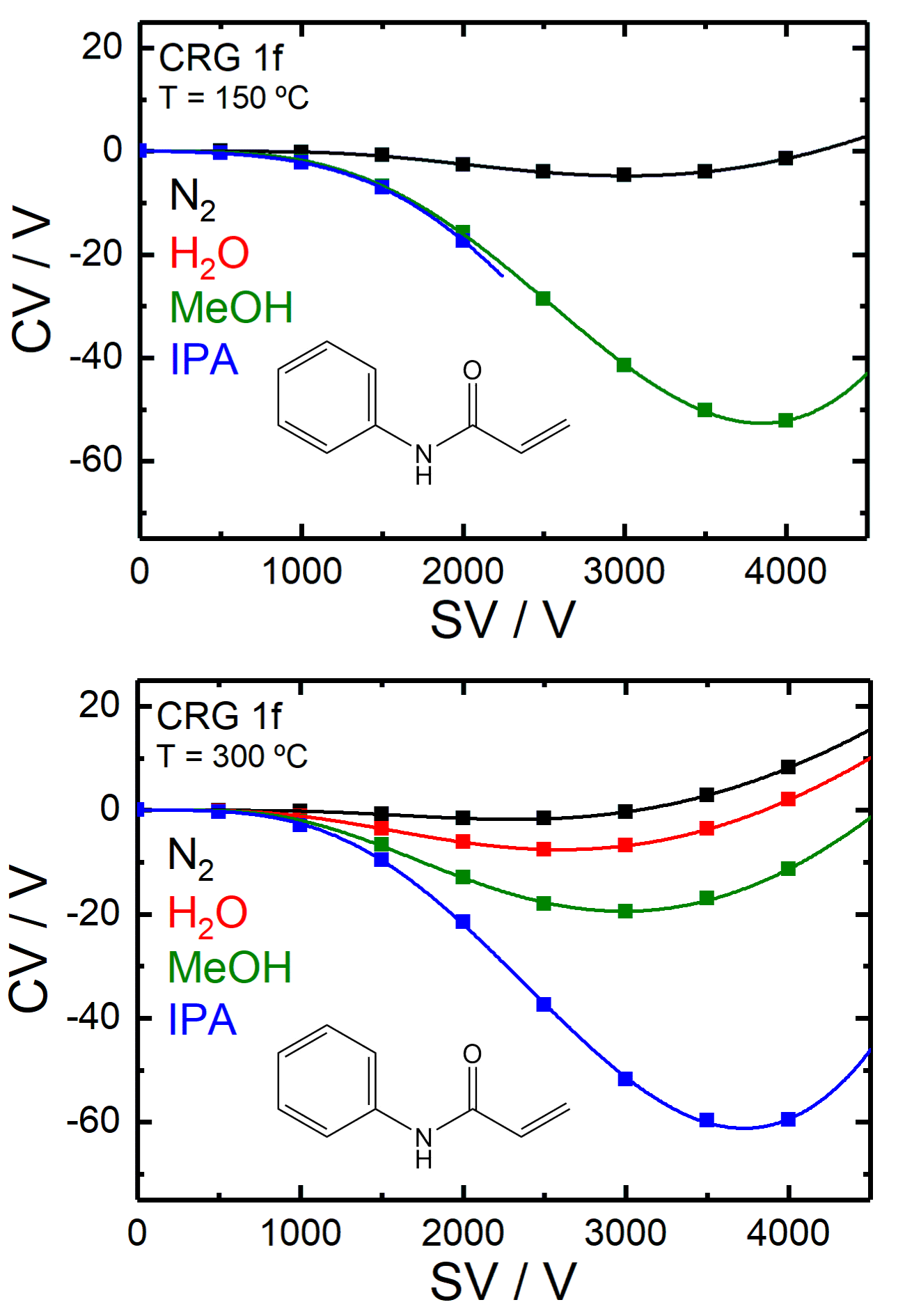


**Supplementary Figure 28.** Dispersion plots for CRG 1f. All data acquired in (black) a pure N_2_ environment, and N_2_ environments seeded with 1.5 % (v/v) (red) water, (green) methanol, and (blue) isopropanol vapor at temperatures of 150 °C and 300 °C. The ion signal for the T = 150 °C water-modified experiment was too low for accurate dispersion plot measurement.


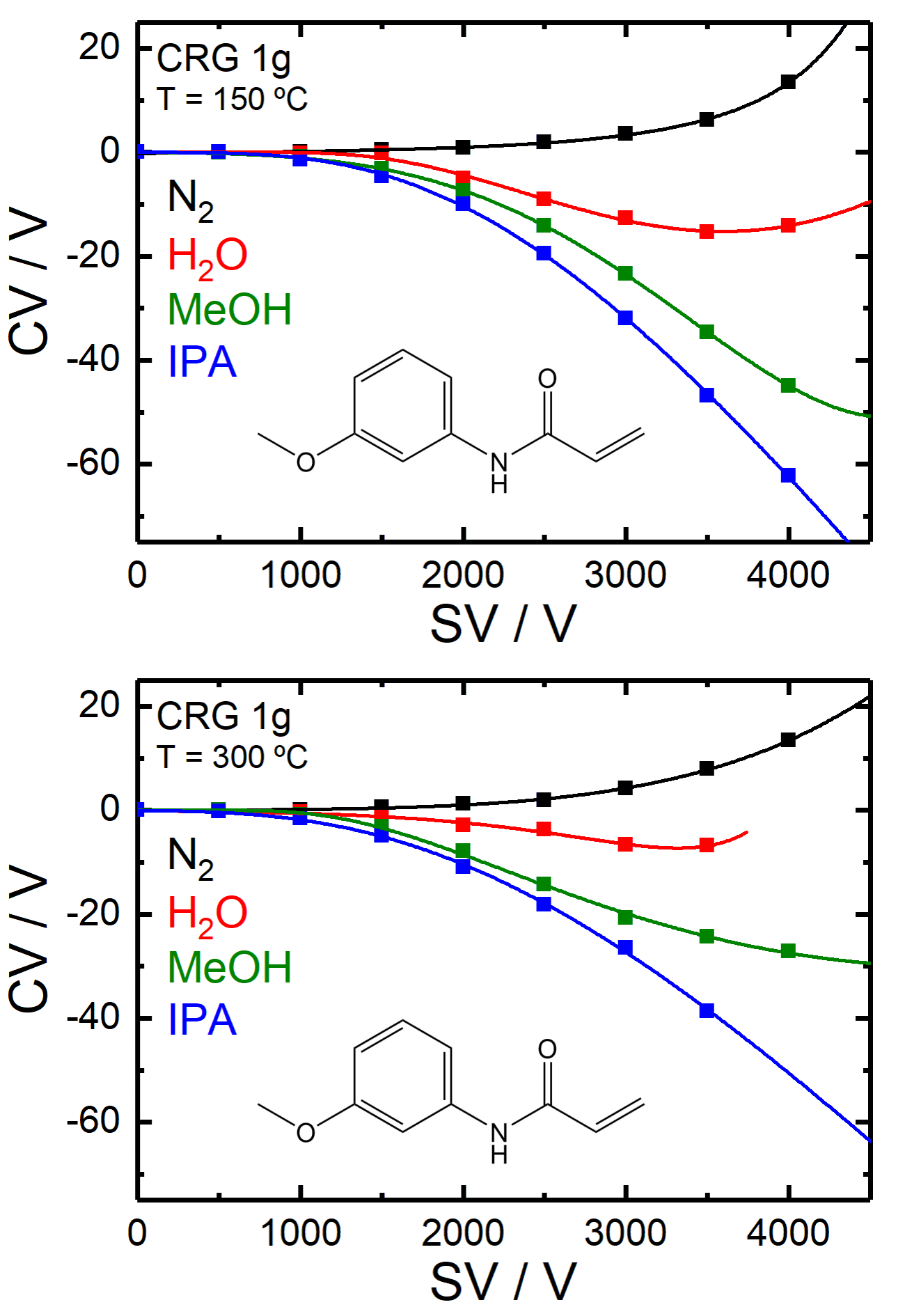


**Supplementary Figure 29.** Dispersion plots for CRG 1g. All data acquired in (black) a pure N_2_ environment, and N_2_ environments seeded with 1.5 % (v/v) (red) water, (green) methanol, and (blue) isopropanol vapor at temperatures of 150 °C and 300 °C.


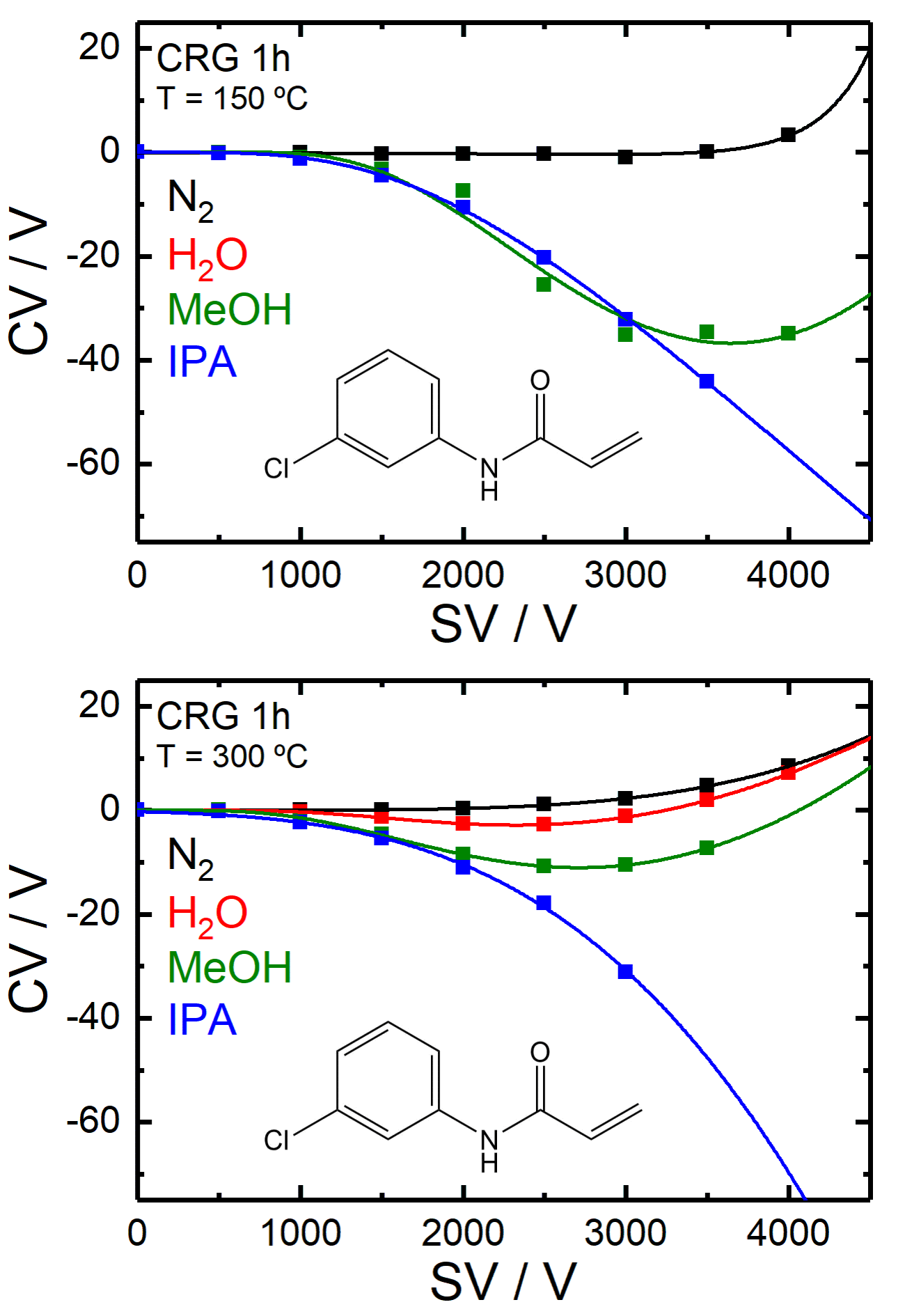


**Supplementary Figure 30.** Dispersion plots for CRG 1h. All data acquired in (black) a pure N_2_ environment, and N_2_ environments seeded with 1.5 % (v/v) (red) water, (green) methanol, and (blue) isopropanol vapor at temperatures of 150 °C and 300 °C. The ion signal for the T = 150 °C water-modified experiment was too low for accurate dispersion plot measurement.


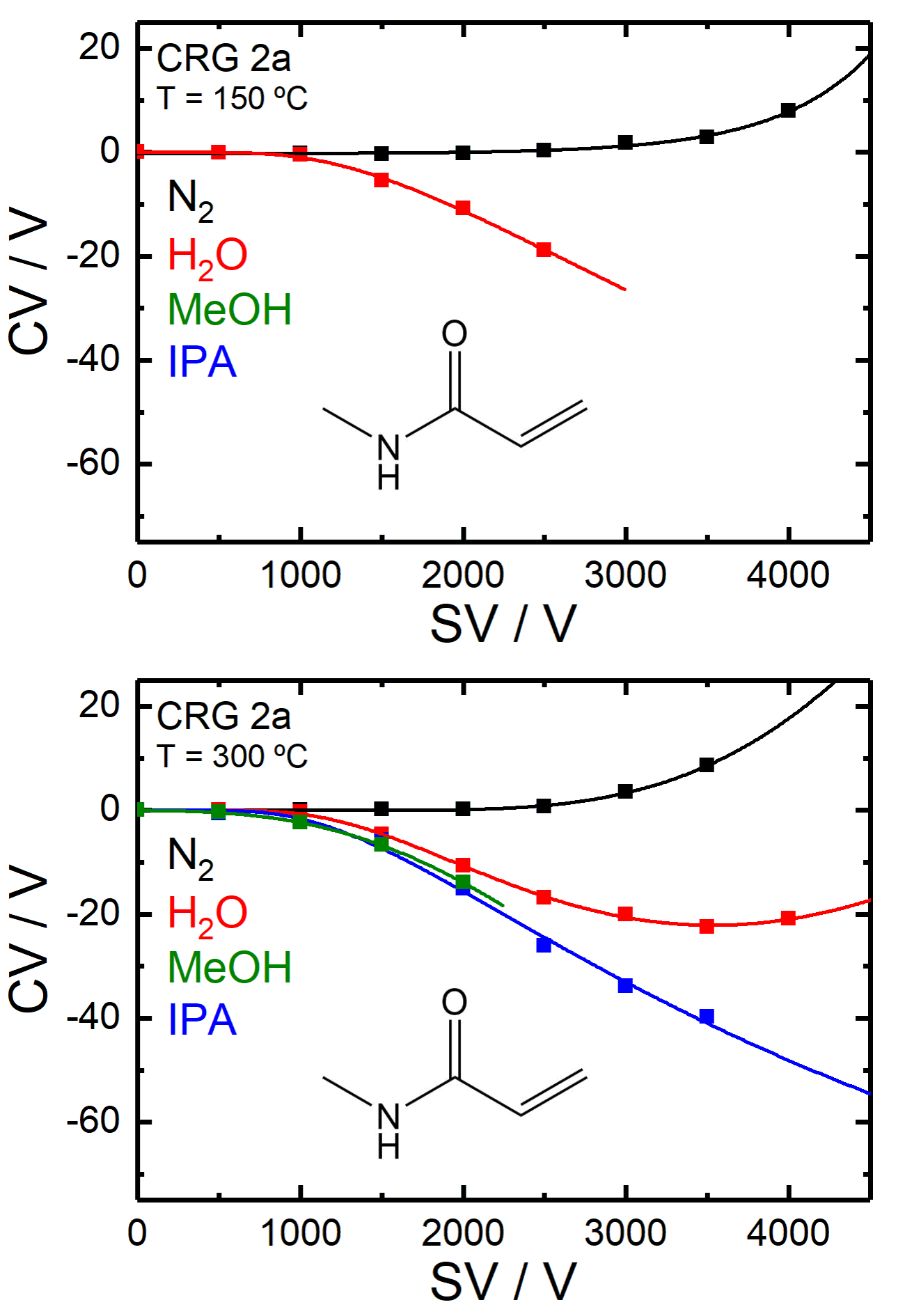


**Supplementary Figure 31.** Dispersion plots for CRG 2a. All data acquired in (black) a pure N_2_ environment, and N_2_ environments seeded with 1.5 % (v/v) (red) water, (green) methanol, and (blue) isopropanol vapor at temperatures of 150 °C and 300 °C. The ion signals for the T = 150 °C methanol-modified and IPA-modified experiments were too low for accurate dispersion plot measurement.


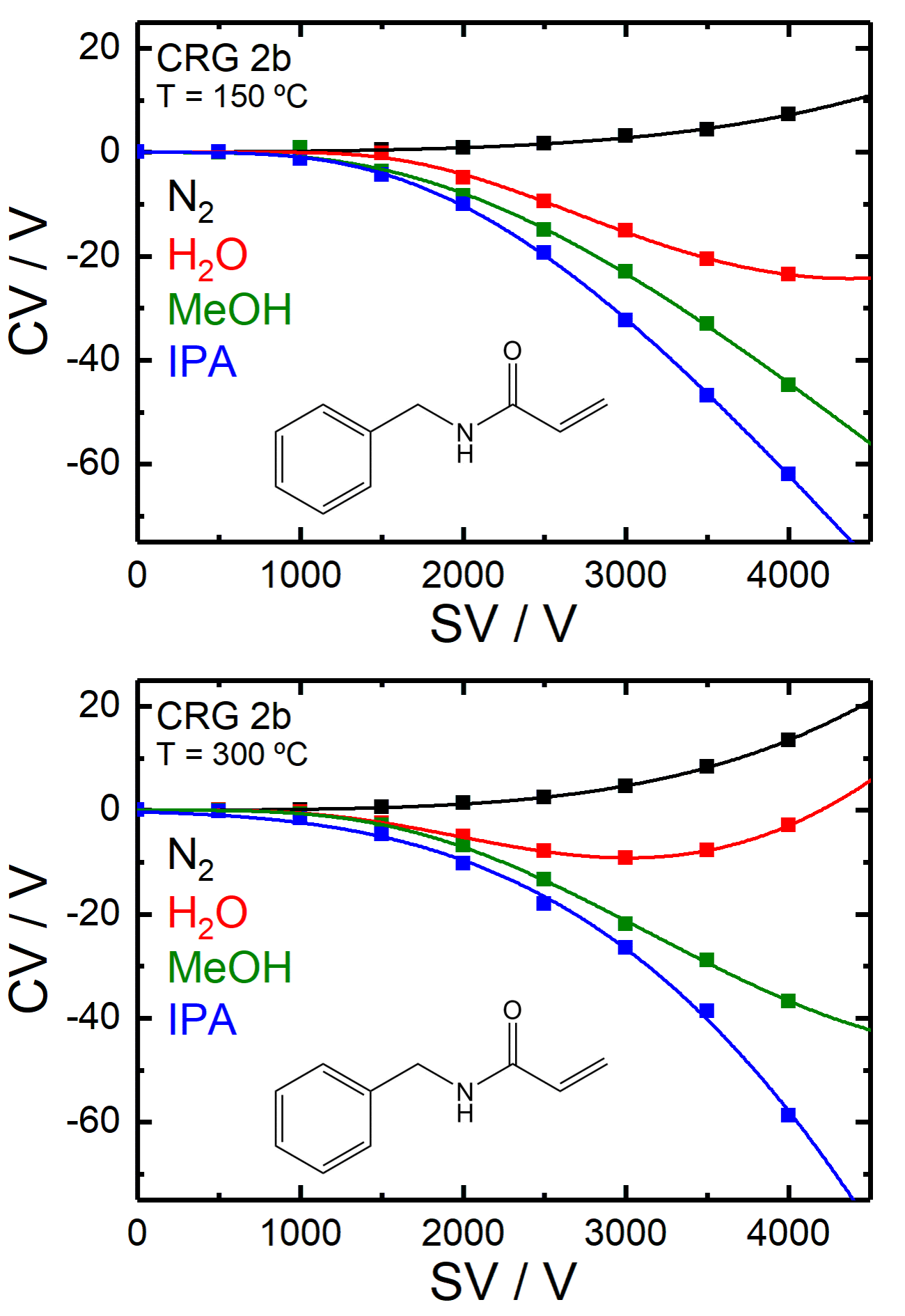


**Supplementary Figure 32.** Dispersion plots for CRG 2b. All data acquired in (black) a pure N_2_ environment, and N_2_ environments seeded with 1.5 % (v/v) (red) water, (green) methanol, and (blue) isopropanol vapor at temperatures of 150 °C and 300 °C.


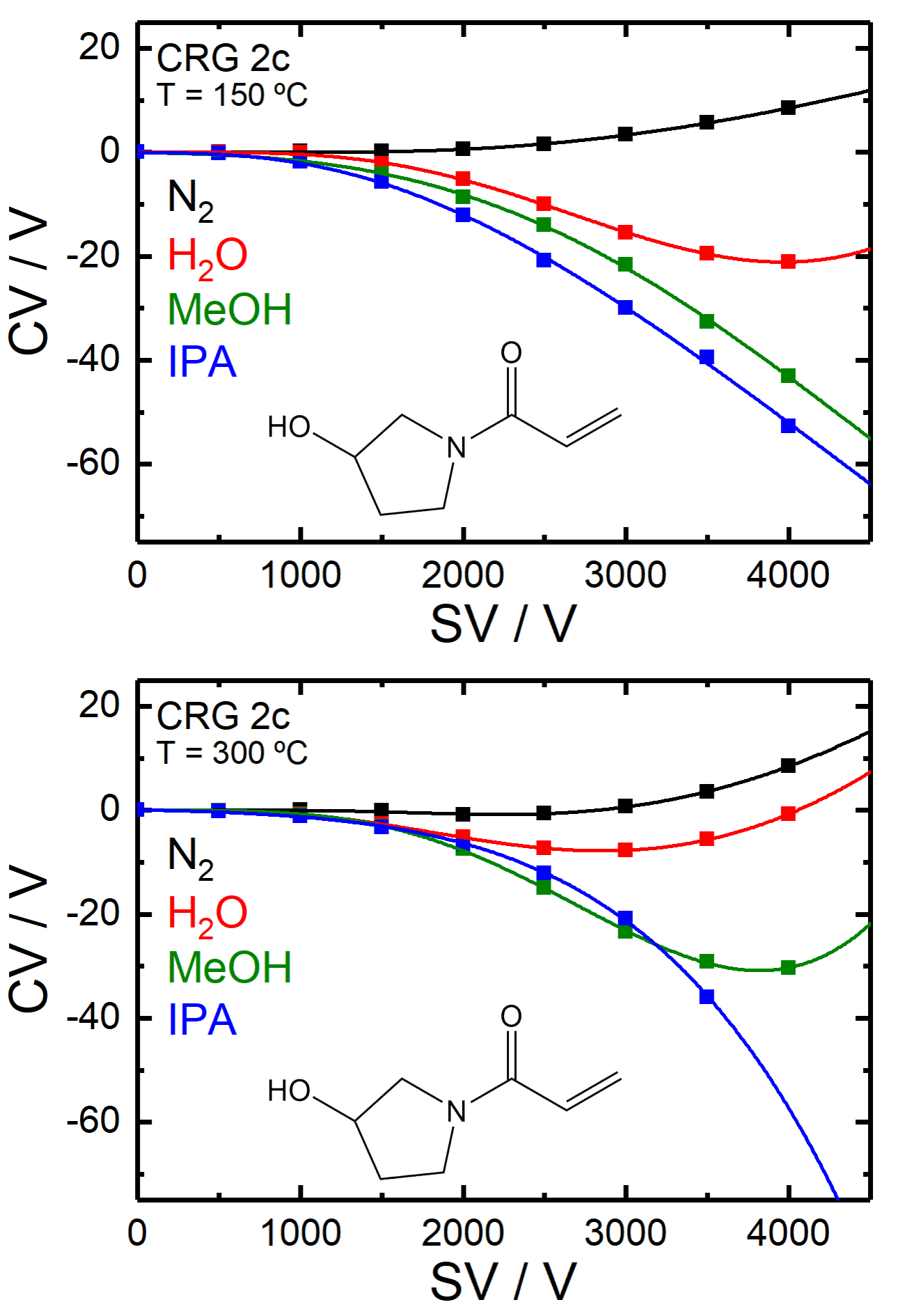


**Supplementary Figure 33.** Dispersion plots for CRG 2c. All data acquired in (black) a pure N_2_ environment, and N_2_ environments seeded with 1.5 % (v/v) (red) water, (green) methanol, and (blue) isopropanol vapor at temperatures of 150 °C and 300 °C.


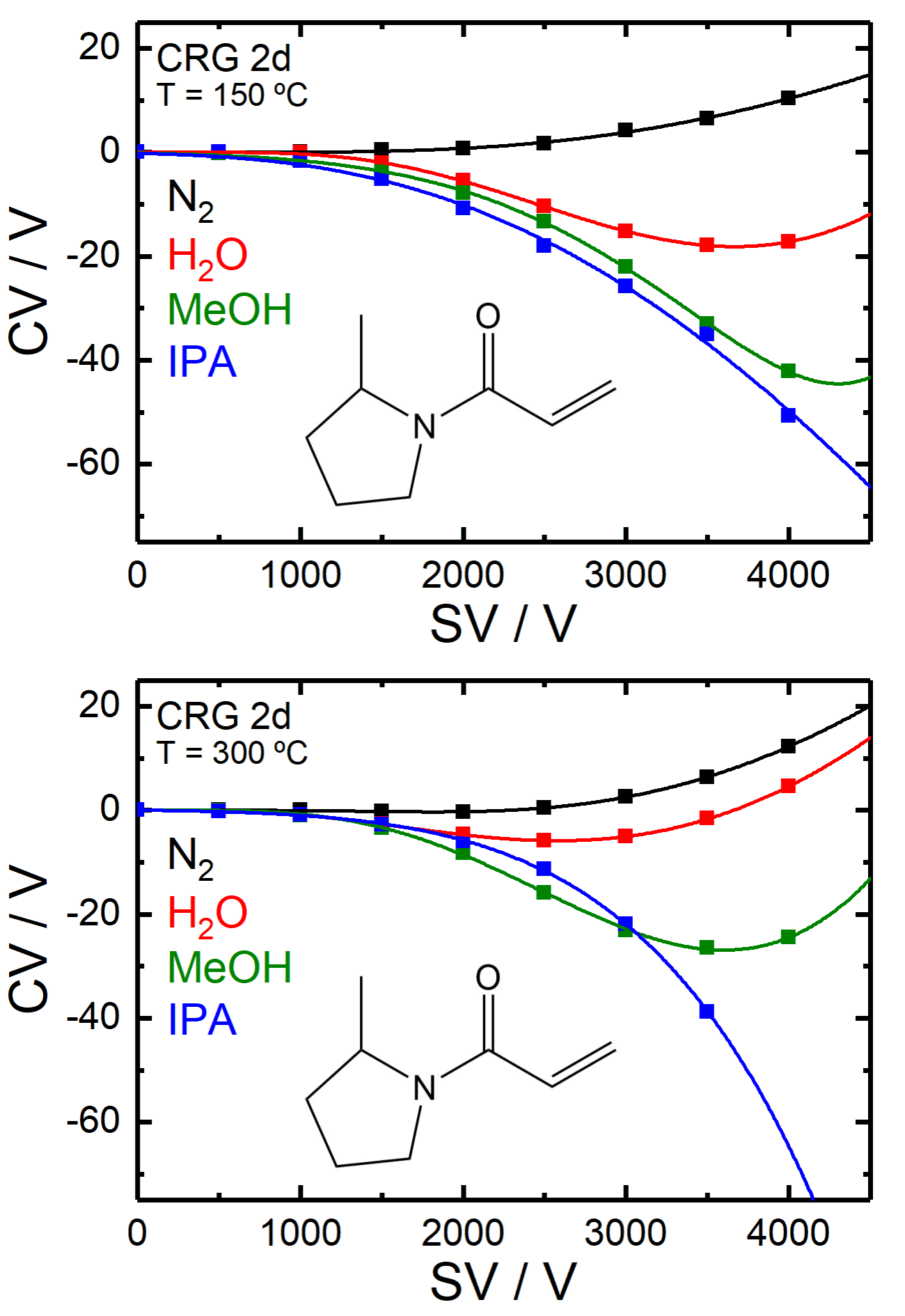


**Supplementary Figure 34.** Dispersion plots for CRG 2d. All data acquired in (black) a pure N_2_ environment, and N_2_ environments seeded with 1.5 % (v/v) (red) water, (green) methanol, and (blue) isopropanol vapor at temperatures of 150 °C and 300 °C.


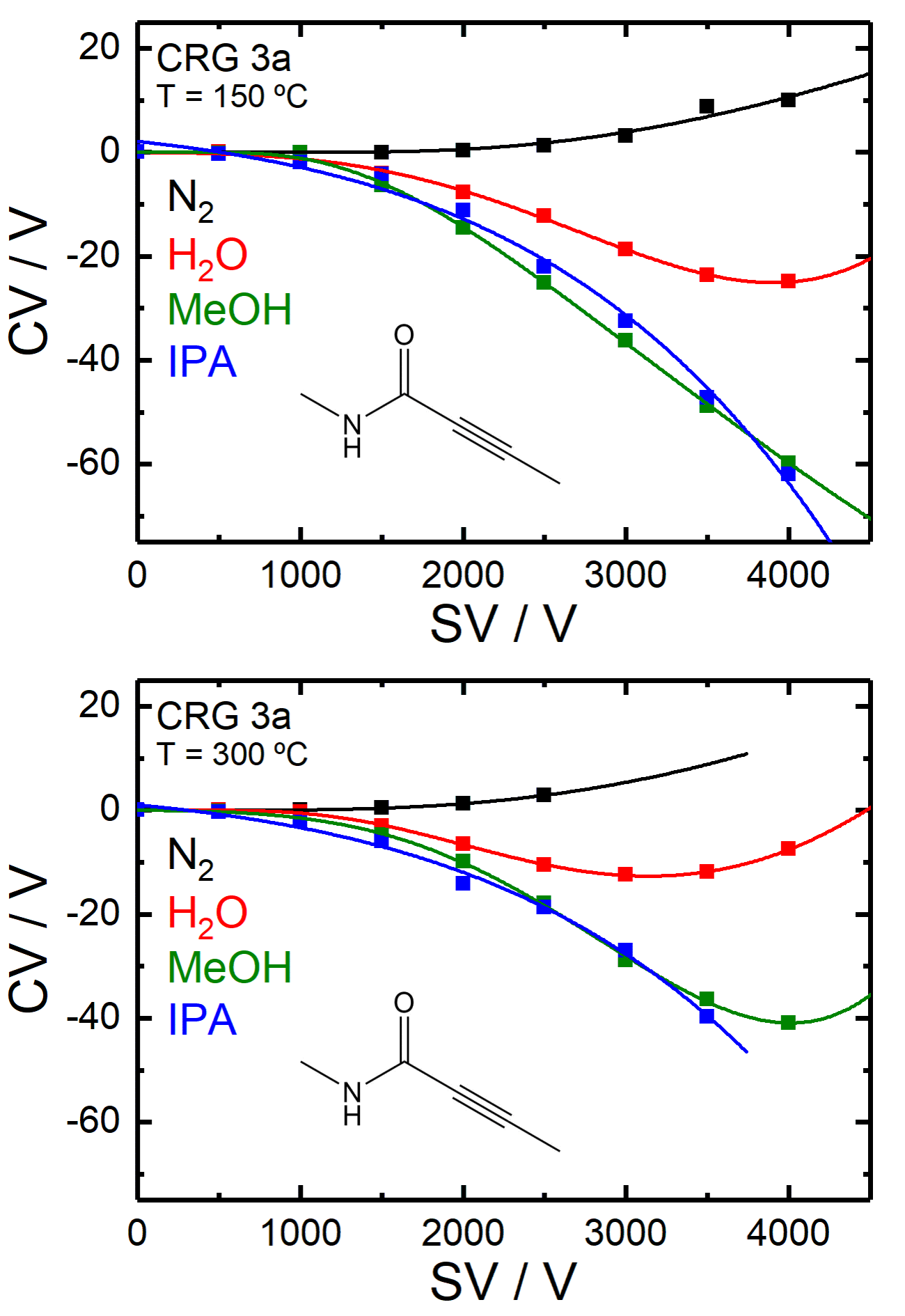


**Supplementary Figure 35.** Dispersion plots for CRG 3a. All data acquired in (black) a pure N_2_ environment, and N_2_ environments seeded with 1.5 % (v/v) (red) water, (green) methanol, and (blue) isopropanol vapor at temperatures of 150 °C and 300 °C.


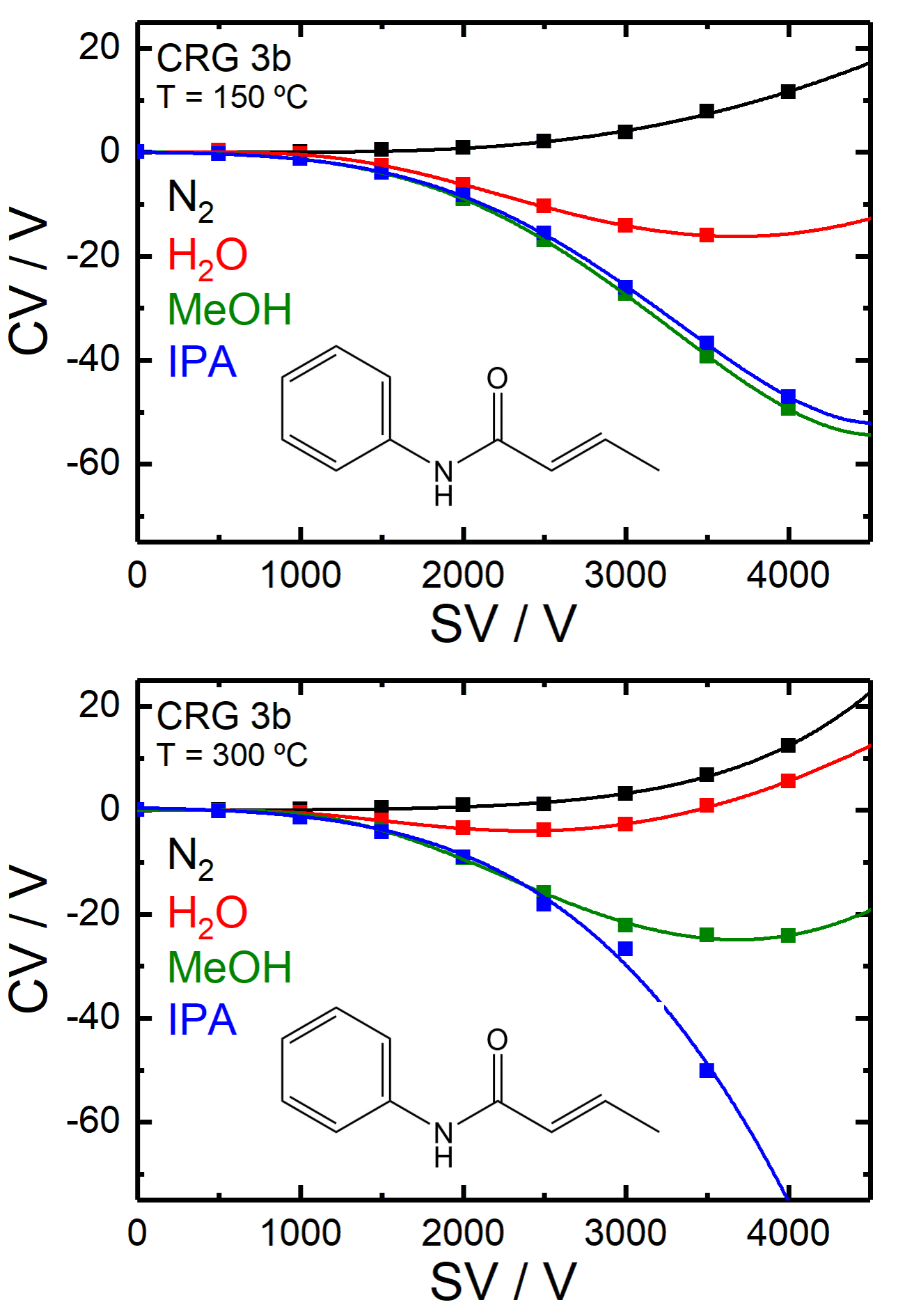


**Supplementary Figure 36.** Dispersion plots for CRG 3b. All data acquired in (black) a pure N_2_ environment, and N_2_ environments seeded with 1.5 % (v/v) (red) water, (green) methanol, and (blue) isopropanol vapor at temperatures of 150 °C and 300 °C.


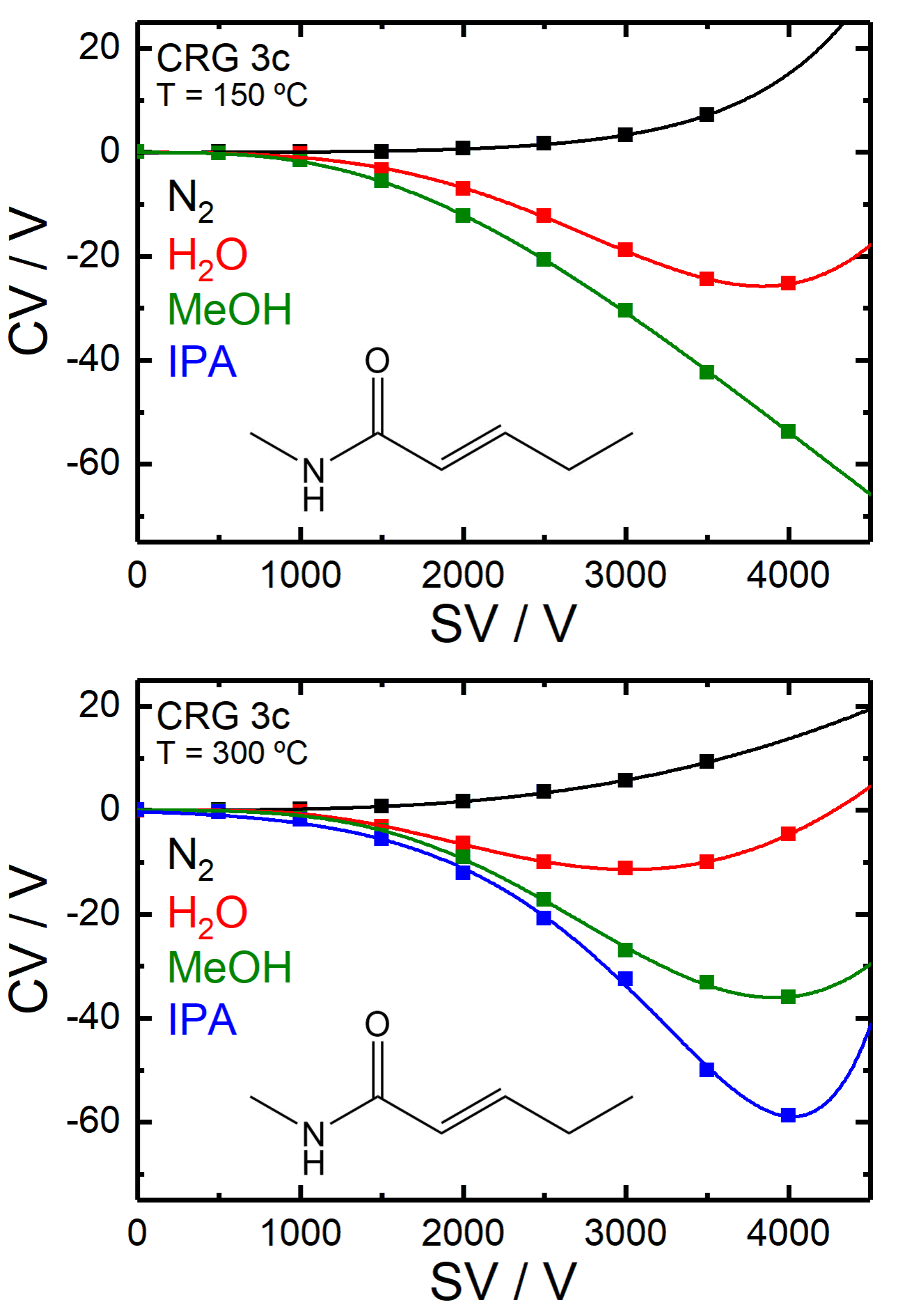


**Supplementary Figure 37.** Dispersion plots for CRG 3c. All data acquired in (black) a pure N_2_ environment, and N_2_ environments seeded with 1.5 % (v/v) (red) water, (green) methanol, and (blue) isopropanol vapor at temperatures of 150 °C and 300 °C. The ion signals for the T = 150 °C IPA-modified experiments were too low for accurate dispersion plot measurement.


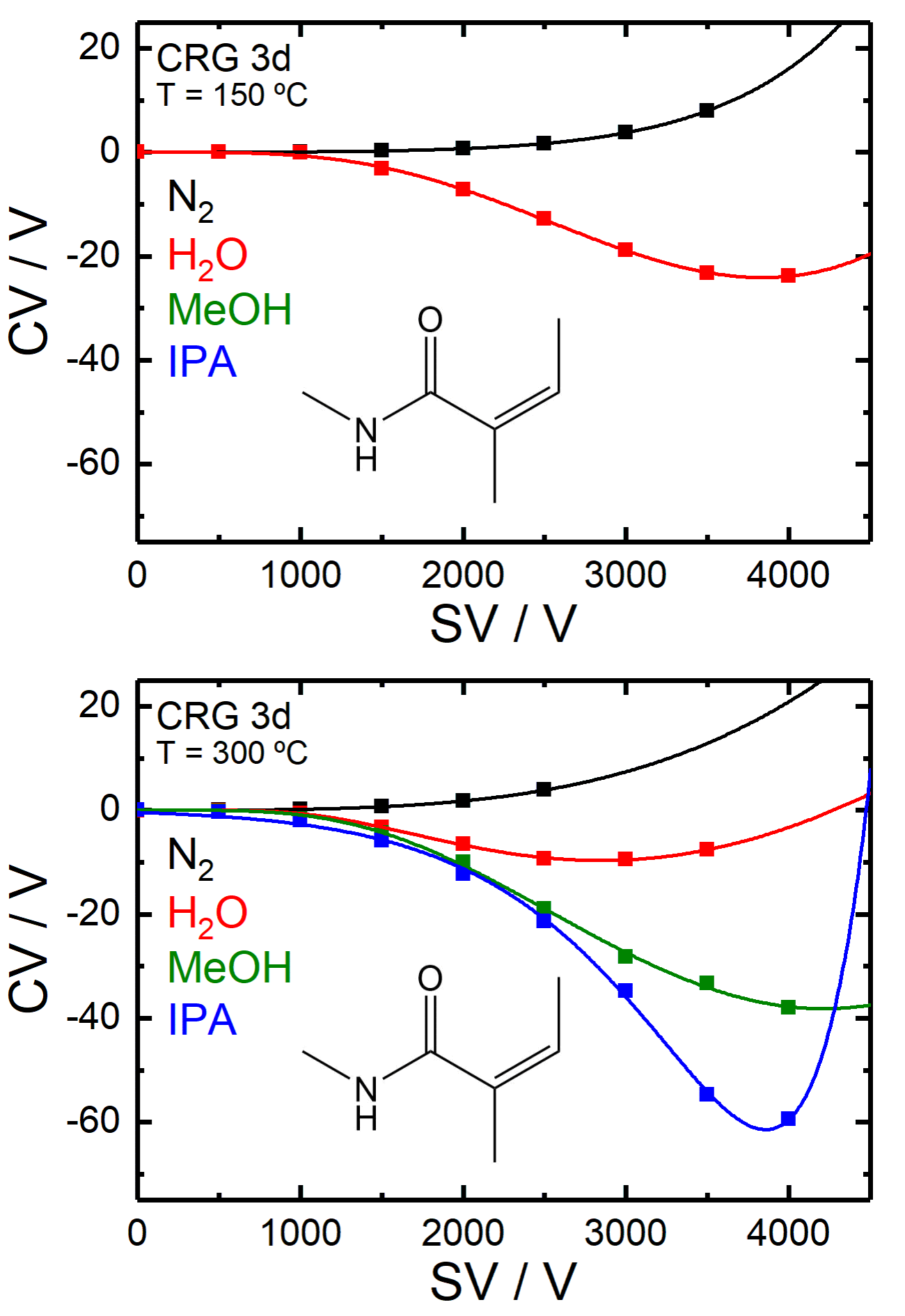


**Supplementary Figure 38.** Dispersion plots for CRG 3d. All data acquired in (black) a pure N_2_ environment, and N_2_ environments seeded with 1.5 % (v/v) (red) water, (green) methanol, and (blue) isopropanol vapor at temperatures of 150 °C and 300 °C. The ion signals for the T = 150 °C methanol-modified and IPA-modified experiments were too low for accurate dispersion plot measurement.


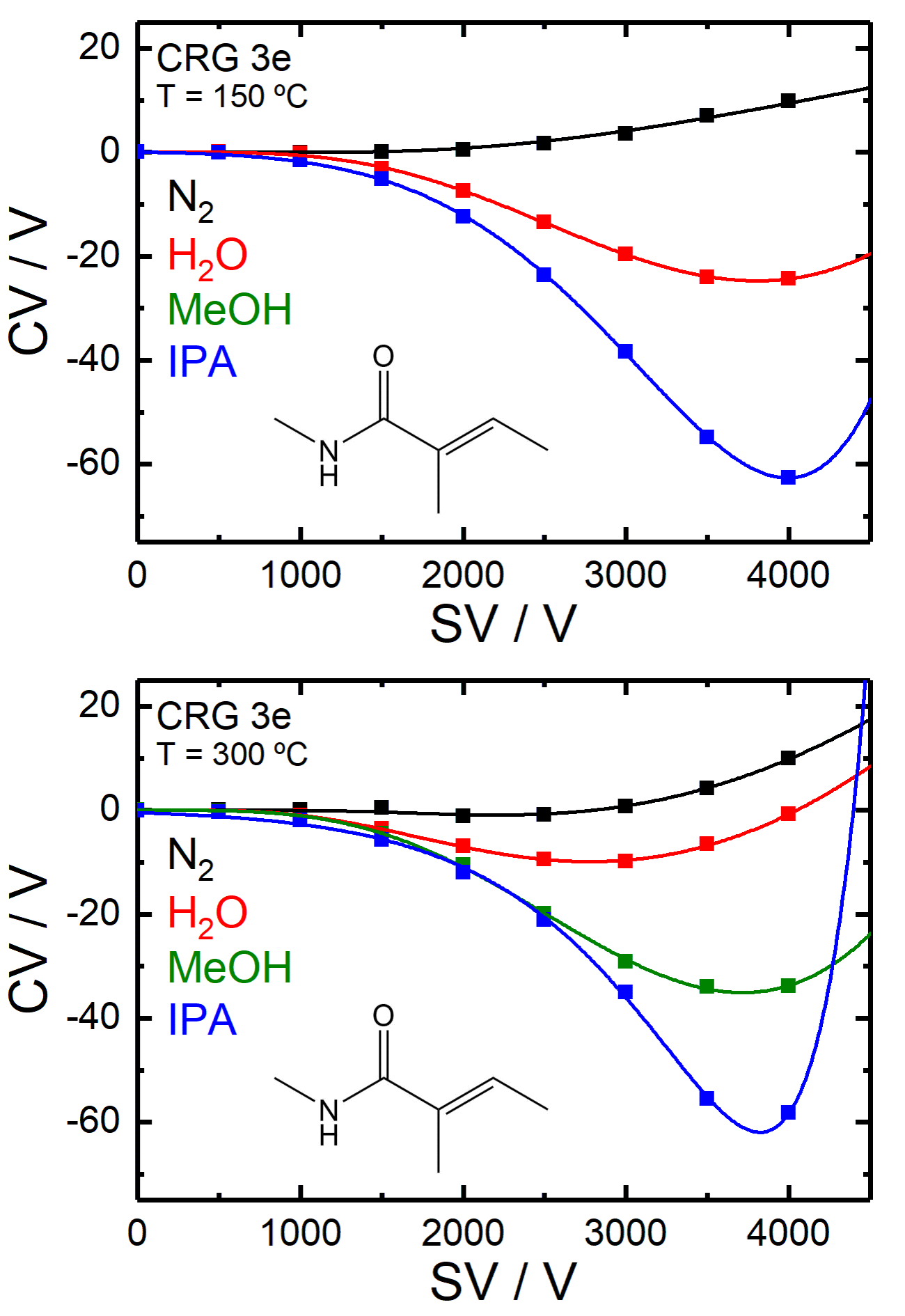


**Supplementary Figure 39.** Dispersion plots for CRG 3e. All data acquired in (black) a pure N_2_ environment, and N_2_ environments seeded with 1.5 % (v/v) (red) water, (green) methanol, and (blue) isopropanol vapor at temperatures of 150 °C and 300 °C. The ion signals for the T = 150 °C methanol-modified experiments were too low for accurate dispersion plot measurement.


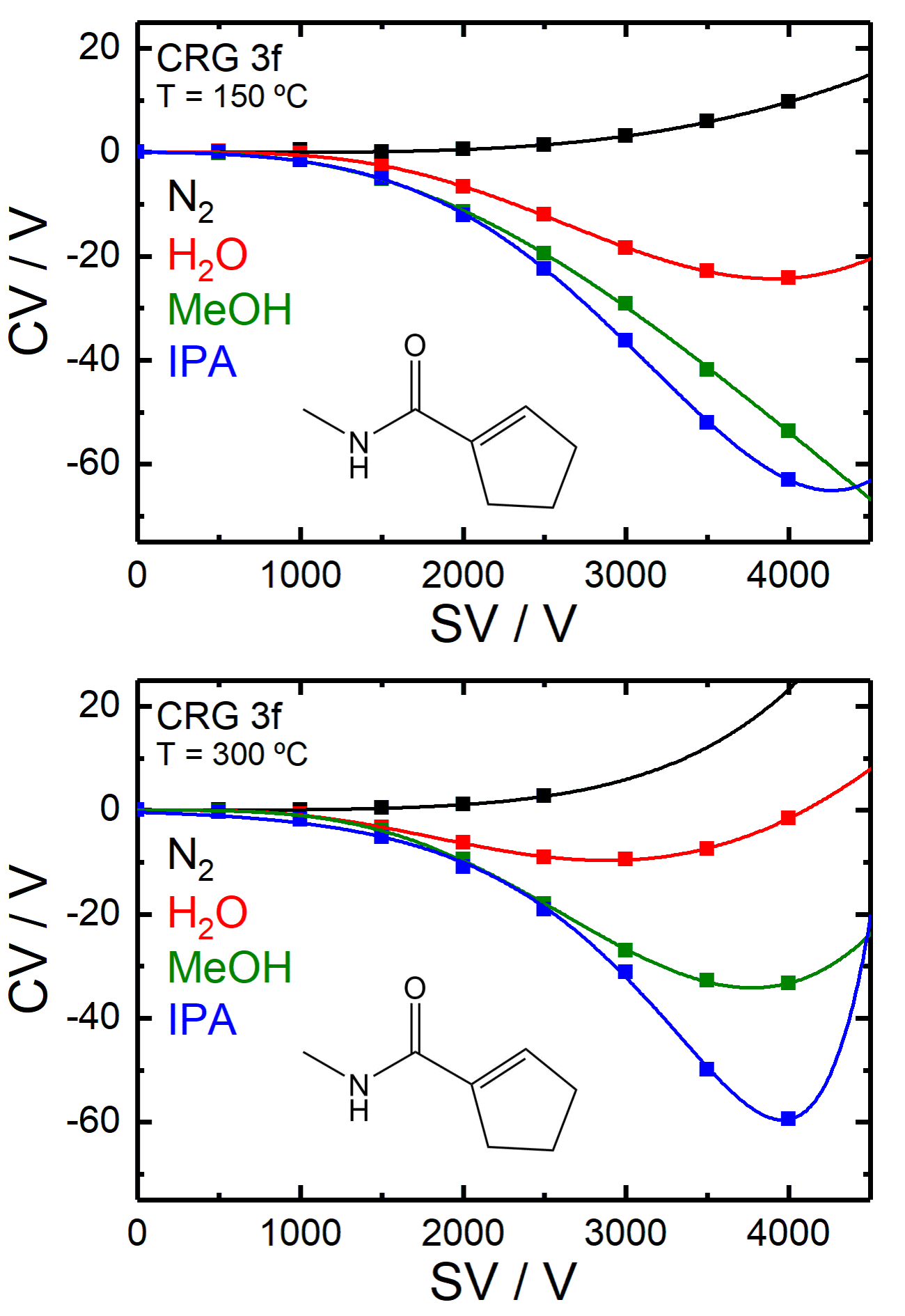


**Supplementary Figure 40.** Dispersion plots for CRG 3f. All data acquired in (black) a pure N_2_ environment, and N_2_ environments seeded with 1.5 % (v/v) (red) water, (green) methanol, and (blue) isopropanol vapor at temperatures of 150 °C and 300 °C.


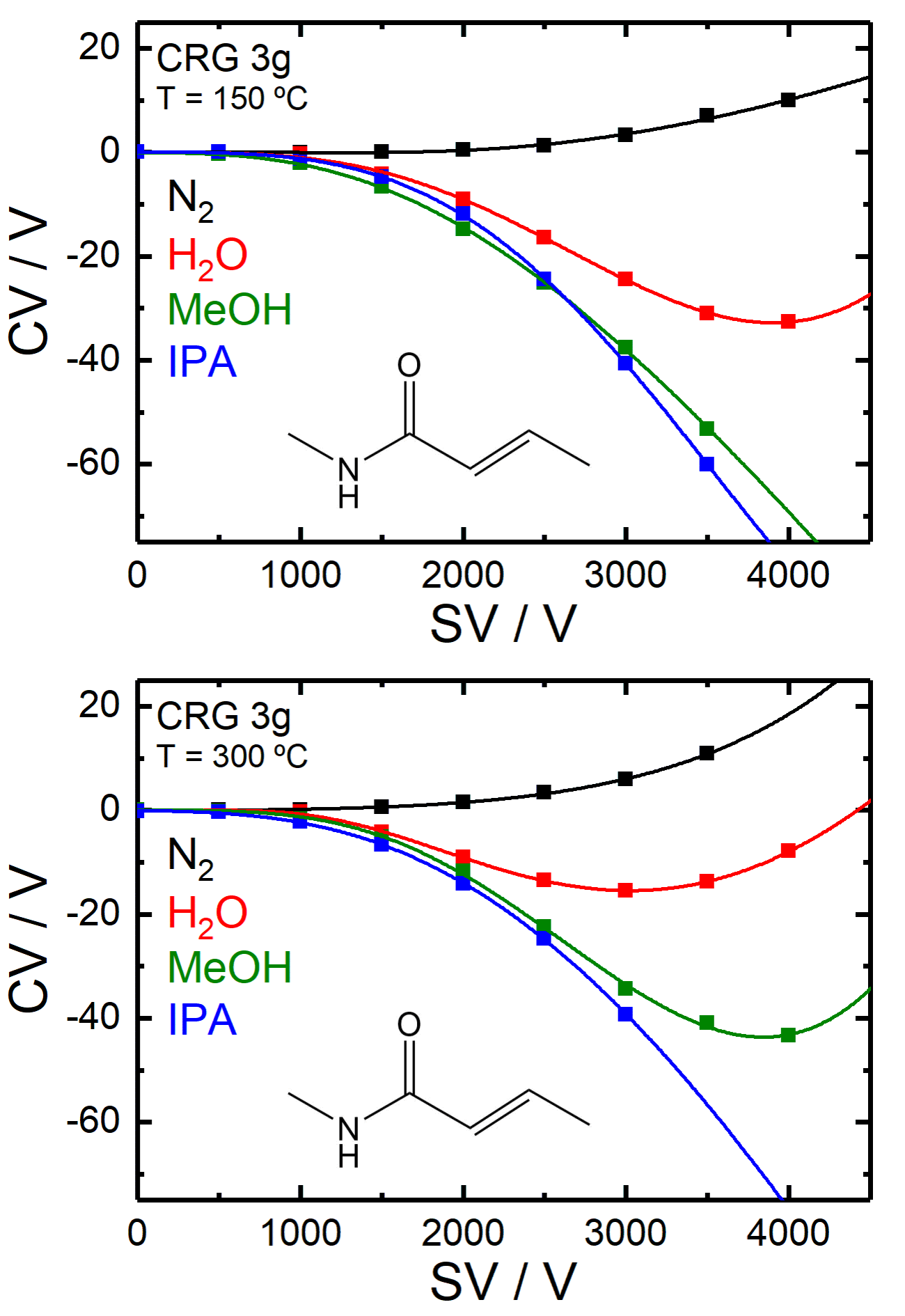


**Supplementary Figure 41.** Dispersion plots for CRG 3g. All data acquired in (black) a pure N_2_ environment, and N_2_ environments seeded with 1.5 % (v/v) (red) water, (green) methanol, and (blue) isopropanol vapor at temperatures of 150 °C and 300 °C.


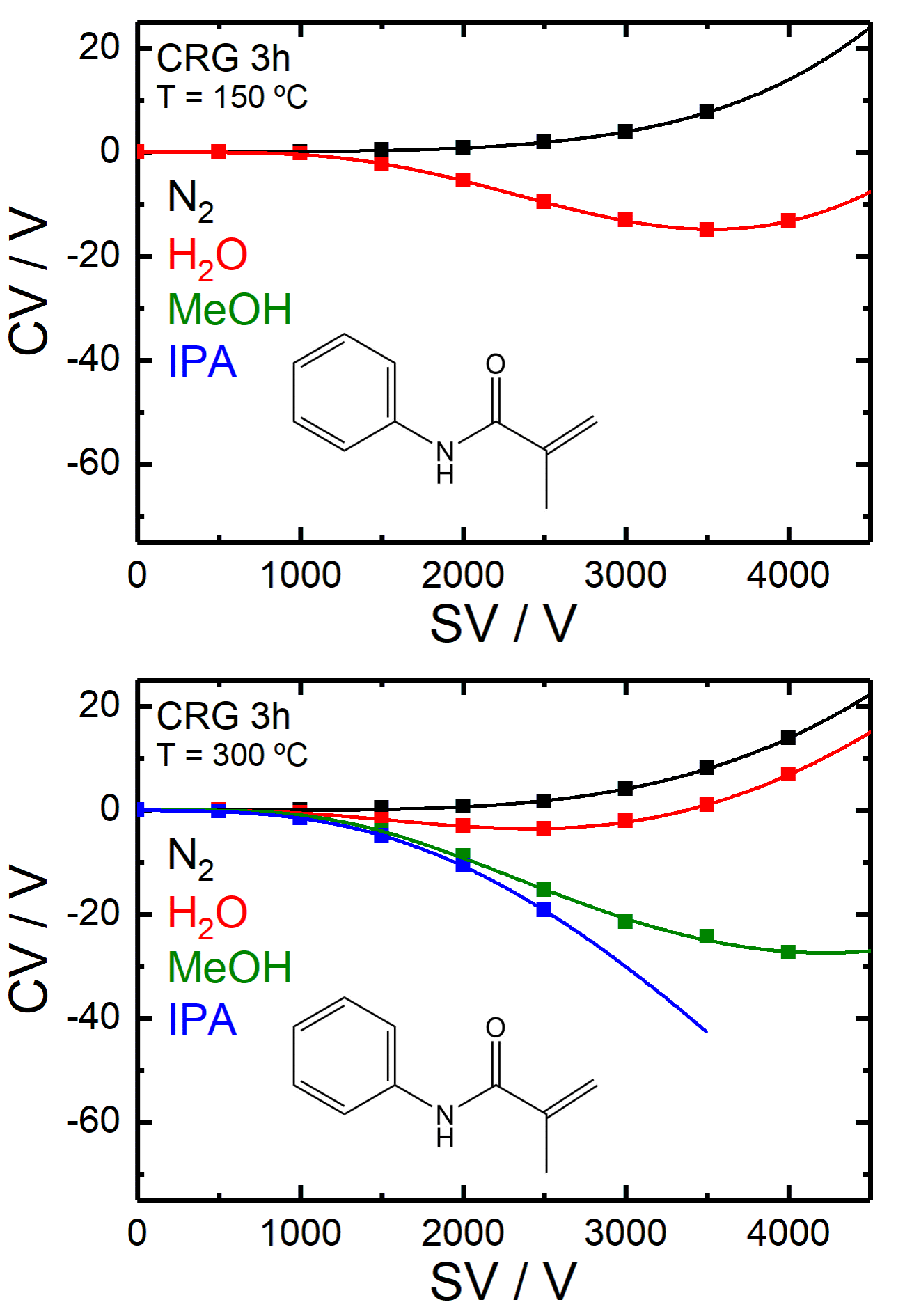


**Supplementary Figure 42.** Dispersion plots for CRG 3h. All data acquired in (black) a pure N_2_ environment, and N_2_ environments seeded with 1.5 % (v/v) (red) water, (green) methanol, and (blue) isopropanol vapor at temperatures of 150 °C and 300 °C. The ion signals for the T = 150 °C methanol-modified and IPA-modified experiments were too low for accurate dispersion plot measurement.


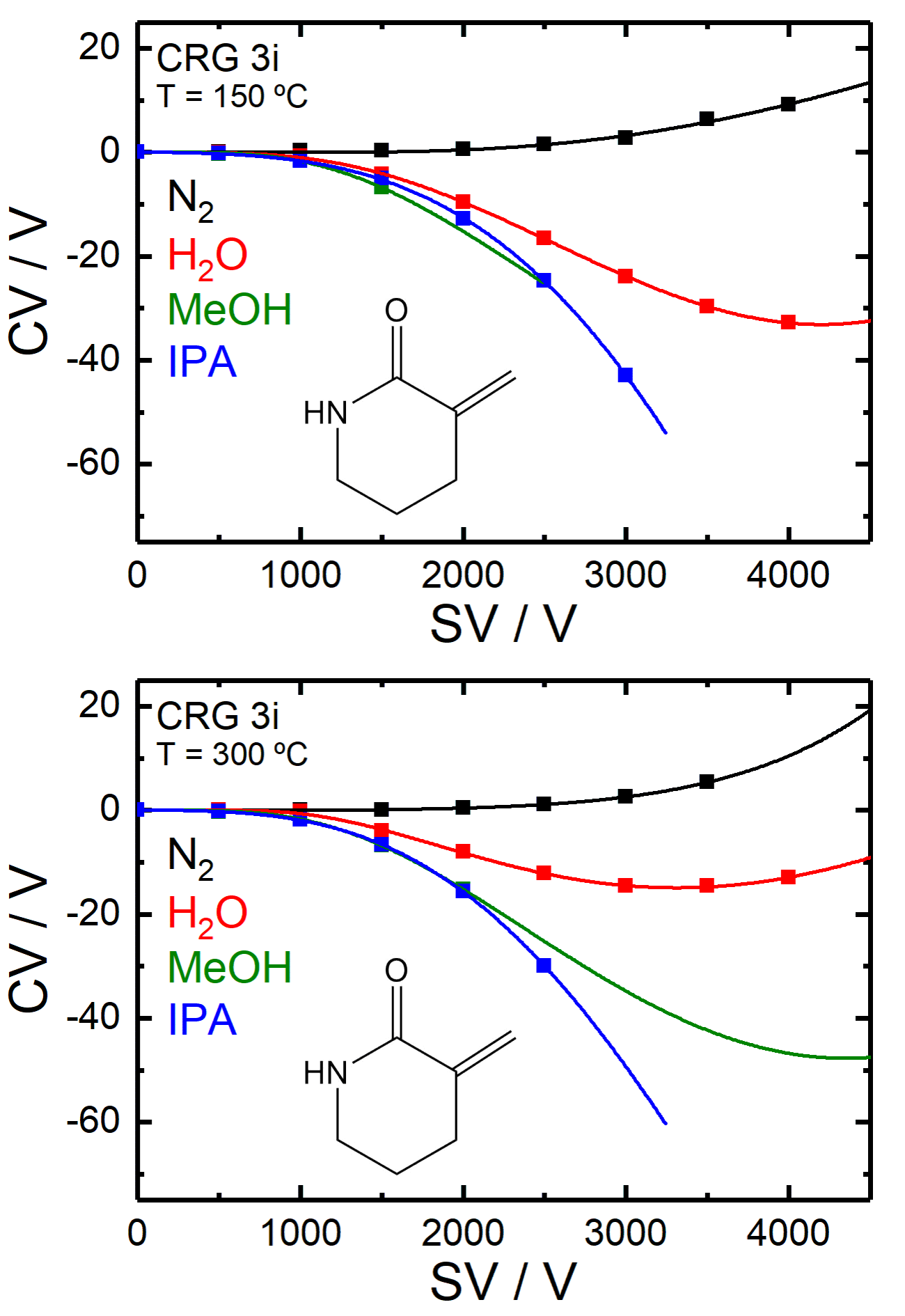


**Supplementary Figure 43.** Dispersion plots for CRG 3i. All data acquired in (black) a pure N_2_ environment, and N_2_ environments seeded with 1.5 % (v/v) (red) water, (green) methanol, and (blue) isopropanol vapor at temperatures of 150 °C and 300 °C.


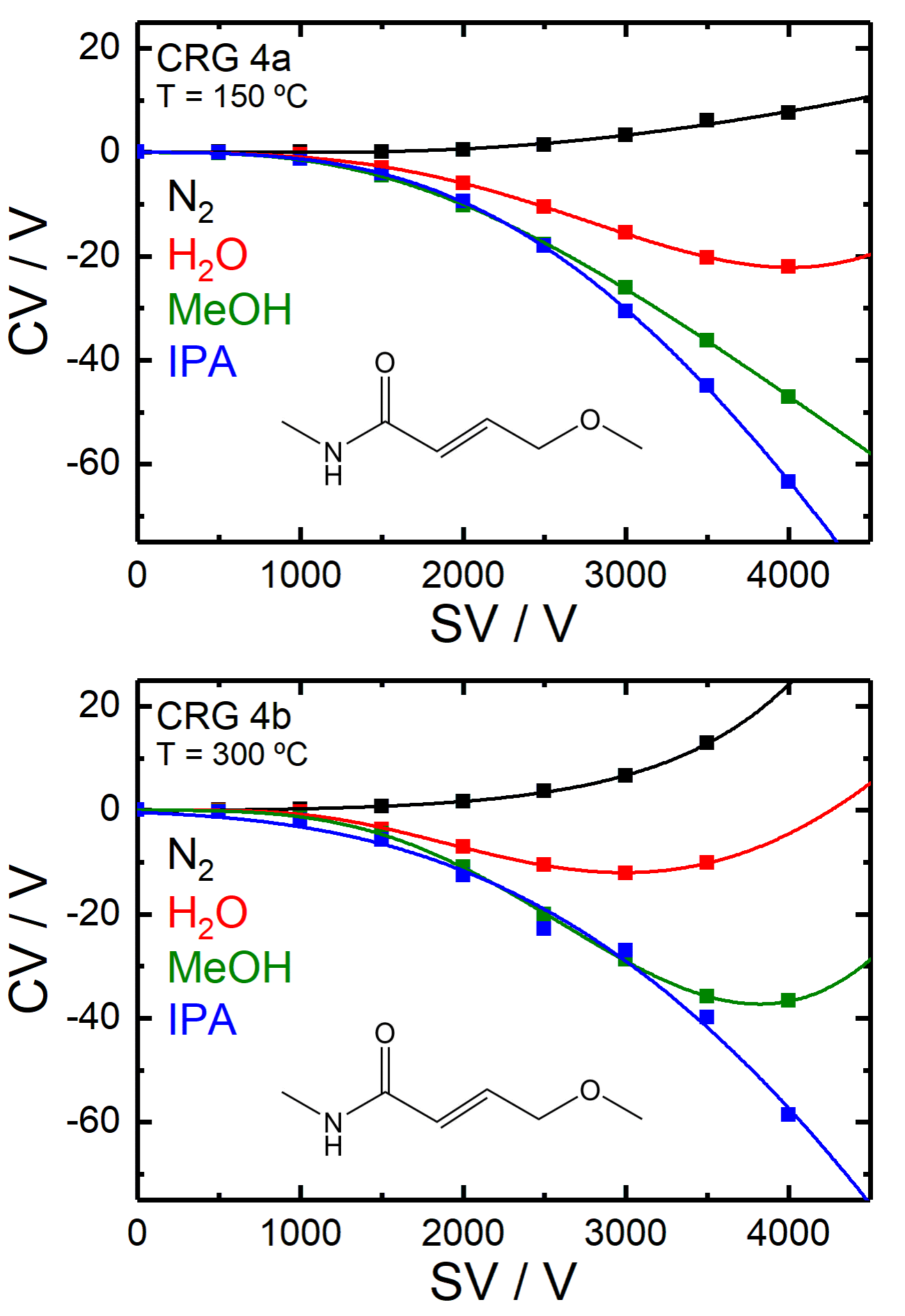


**Supplementary Figure 43.** Dispersion plots for CRG 4a. All data acquired in (black) a pure N_2_ environment, and N_2_ environments seeded with 1.5 % (v/v) (red) water, (green) methanol, and (blue) isopropanol vapor at temperatures of 150 °C and 300 °C.


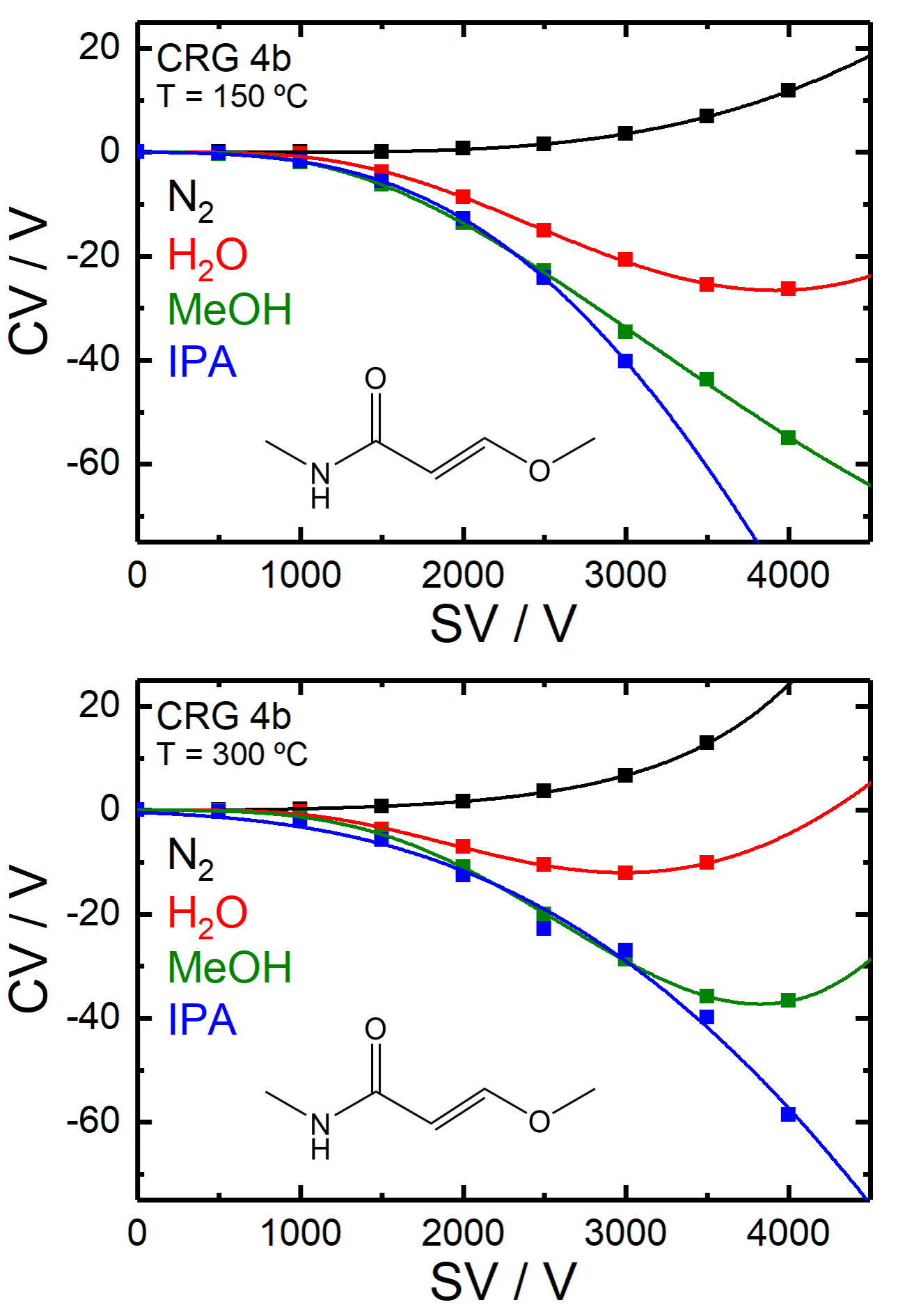


**Supplementary Figure 45.** Dispersion plots for CRG 4b. All data acquired in (black) a pure N_2_ environment, and N_2_ environments seeded with 1.5 % (v/v) (red) water, (green) methanol, and (blue) isopropanol vapor at temperatures of 150 °C and 300 °C.

**
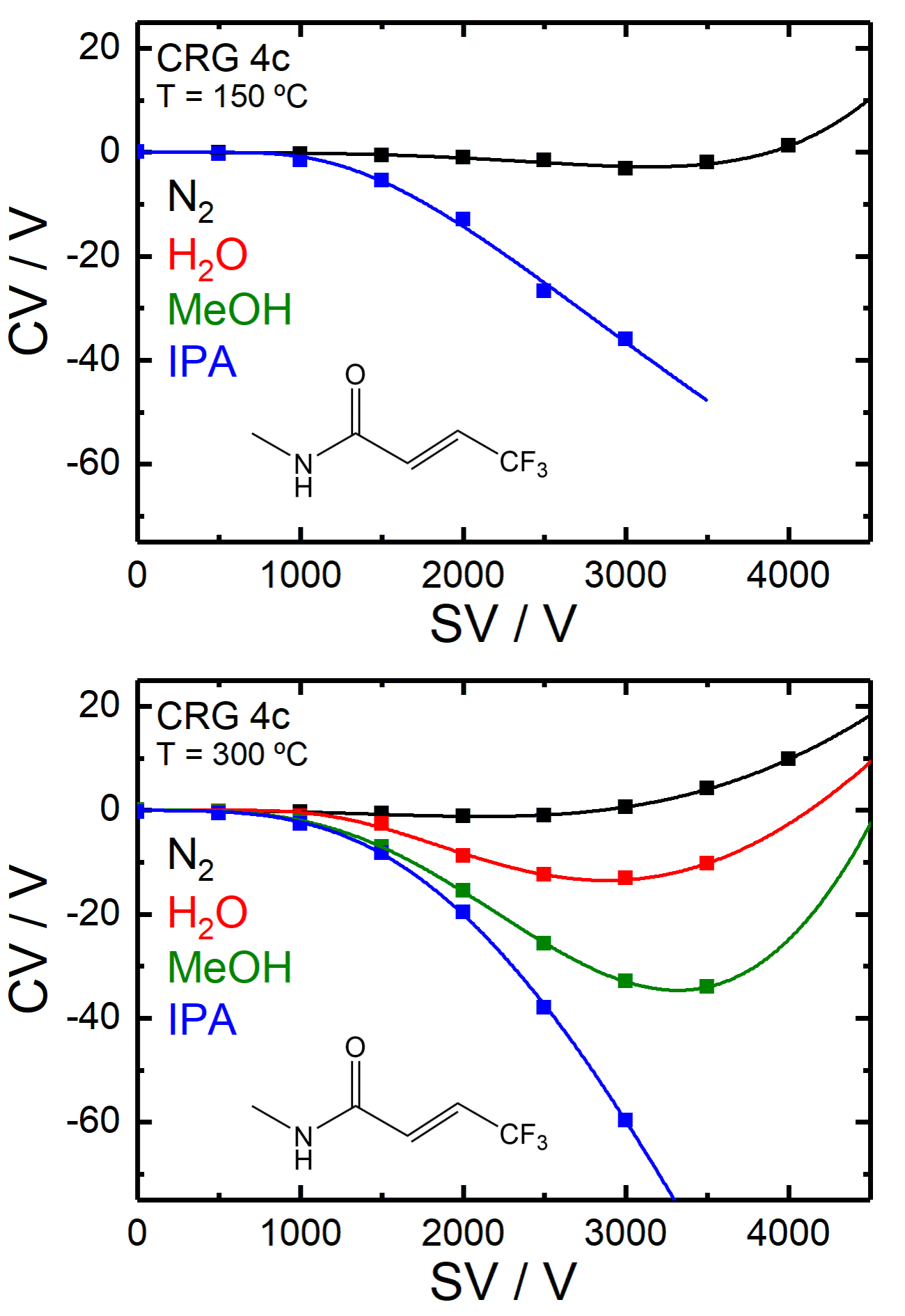
**

**Supplementary Figure 46.** Dispersion plots for CRG 4c. All data acquired in (black) a pure N_2_ environment, and N_2_ environments seeded with 1.5 % (v/v) (red) water, (green) methanol, and (blue) isopropanol vapor at temperatures of 150 °C and 300 °C. The ion signals for the T = 150 °C methanol-modified and water-modified experiments were too low for accurate dispersion plot measurement.

**Supplementary Methods – Computational Details**

To determine the most stable molecular geometries, a custom-written basin hopping (BH) search algorithm was used to map the potential energy surface (PES) of each drug candidate. The BH algorithm has been described in detail elsewhere.[^1^](#_ENREF_1)^,^ [^2^](#_ENREF_2) Briefly, we first identified the most likely site(s) of protonated for each molecule by generating all possible tautomers/protomers and optimizing them individually at the B3LYP/LANL2DZ level of theory. For the most stable structures, atomic partial charges were calculated using the ChelpG partition scheme.[^3^](#_ENREF_3) The cluster PES was then modeled using the Universal Force Field.[^4^](#_ENREF_4) To search the PES, the dihedral angles associated with single bonds were randomly distorted by −5° ≥ θ ≥ +5° at each iteration of the BH code. In total, approximately 20,000 geometries were sampled for each molecule. Unique structures were identified based on zero-point corrected energy and geometry. Unique structures were then carried forward for geometry optimization at the PM7 level of theory for all molecules. To verify that calculations at the PM7 level produced appropriate geometries, the following subset of molecules were also geometry optimized at the B3LYP/6-311++G(d,p) level of theory:

| - A1a - A1b - A4a - A4b - B1a - B1b | - C4a - C4b - C4c - D1a - D1b |
| --- | --- |

For all molecules in the above subset, PM7 calculations produced the same global minimum structure and the same relative energy order of tautomers/protomers as did the B3LYP/6-311++G(d,p) calculations. In addition to geometry optimizations, normal mode analyses were also conducted to ensure that each structure was a local minimum on the PES.

**Supplementary Figure 47.** Random Forest ML output for cell permeability using only CCS as a learner. Input data includes the IMHB dataset and the methylquinoline-8-ol dataset. R^2^ = 0.1285, Root Mean Square Error = 8.14 cm s^-1^, Mean Absolute Error = 5.80 cm s^-1^

**Supplementary Figure 48.** Random Forest ML for cell permeability using only a single SV/CV data point and a random number in place of the calculated molecular CCS. Input data includes the IMHB dataset and the methylquinoline-8-ol dataset. R^2^ = 0.497, Root Mean Square Error = 6.69 cm s^-1^, Mean Absolute Error = 4.94 cm s^-1^

**Supplementary Figure 49.** Random Forest ML output for cell permeability using only a single SV/CV data point and the calculated molecular CCS. Input data includes the IMHB dataset and the methylquinoline-8-ol dataset. R^2^ = 0.4405**,** Root Mean Square Error = 7.11 cm s^-1^**,** Mean Absolute Error = 5.34 cm s^-1^

**Supplementary Figure 50.** Random Forest ML output for cell permeability when using two SV/CV data points (SV = 3750 V, 4000 V) and the calculated molecular CCS. Input data includes the IMHB dataset and the methylquinoline-8-ol dataset. R^2^ = 0.8046, Root Mean Square Error = 4.12 cm s^-1^, Mean Absolute Error = 3.09 cm s^-1^


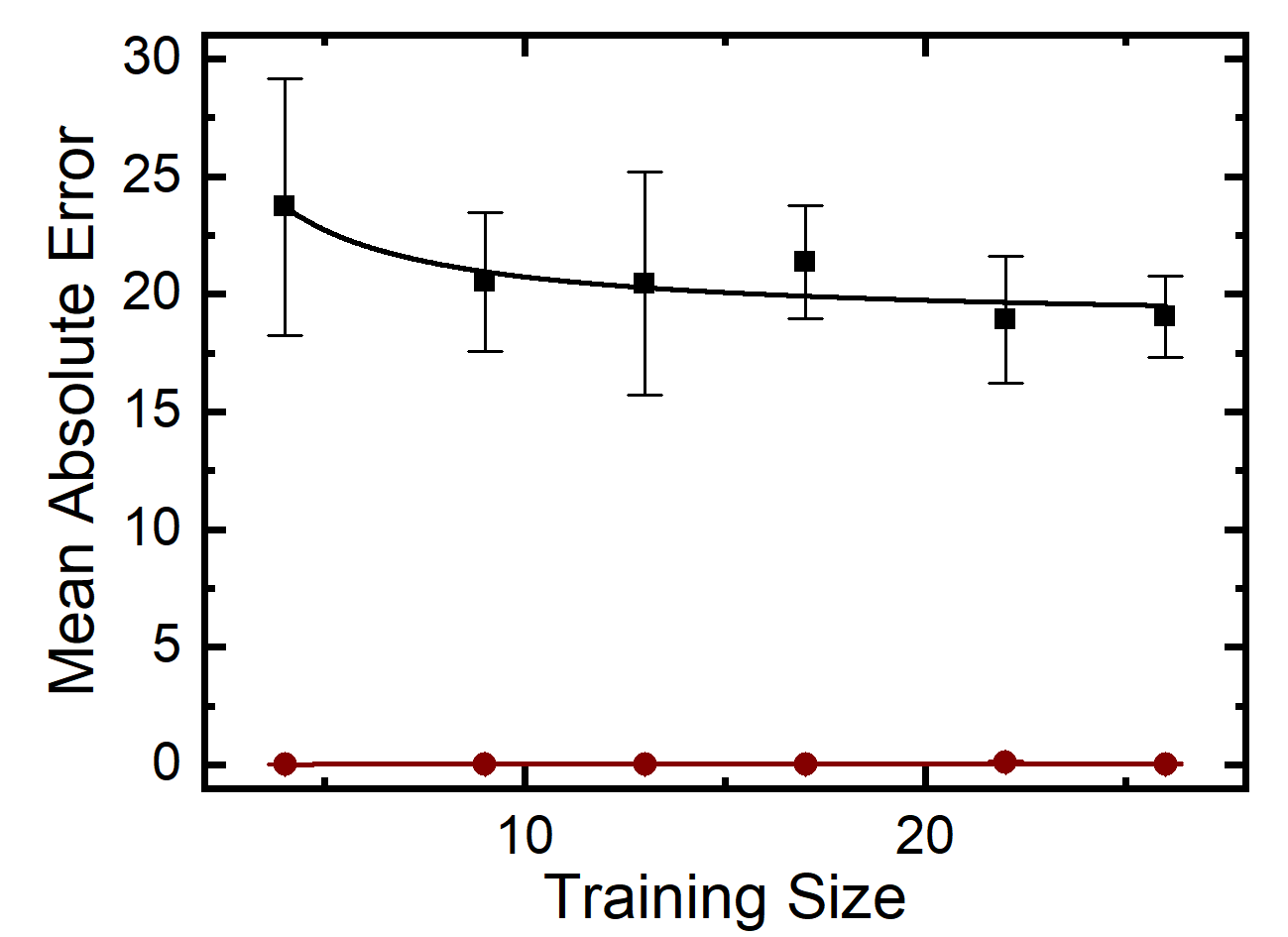


**Supplementary Figure 51.** Learning curve for EPSA ML fits. ML fits conducted as described in the text. Number of molecules refers to the size of the training set. In total the database contains measured EPSA values for 43 molecules. Error bars are calculated as 1 standard deviation of the average mean absolute error.


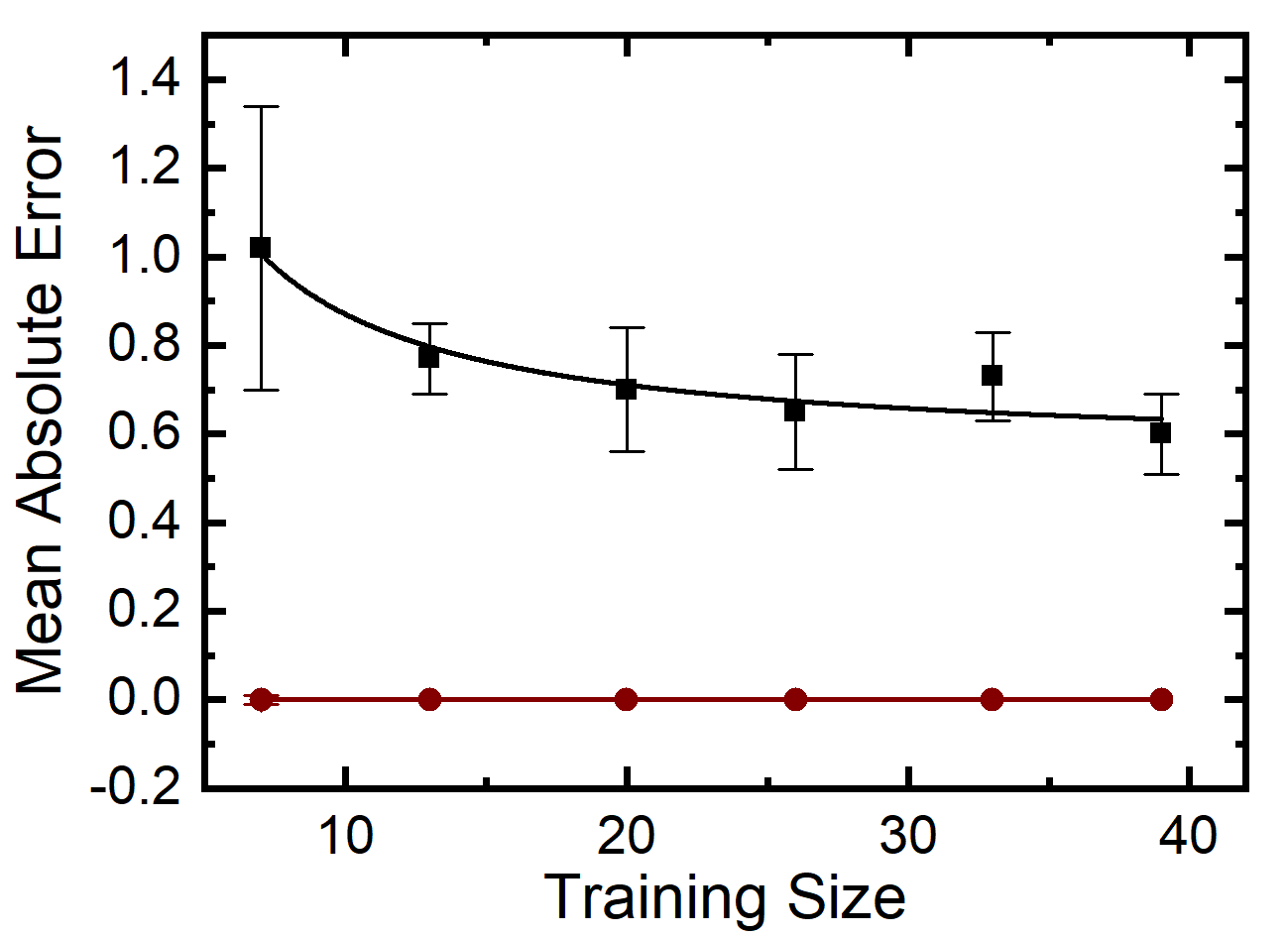


**Supplementary Figure 52**. Learning curve for LogD ML fits. ML fits conducted as described in the text. Number of molecules refers to the size of the training set. In total the database contains measured LogD values for 65 molecules. Error bars are calculated as 1 standard deviation of the average mean absolute error.


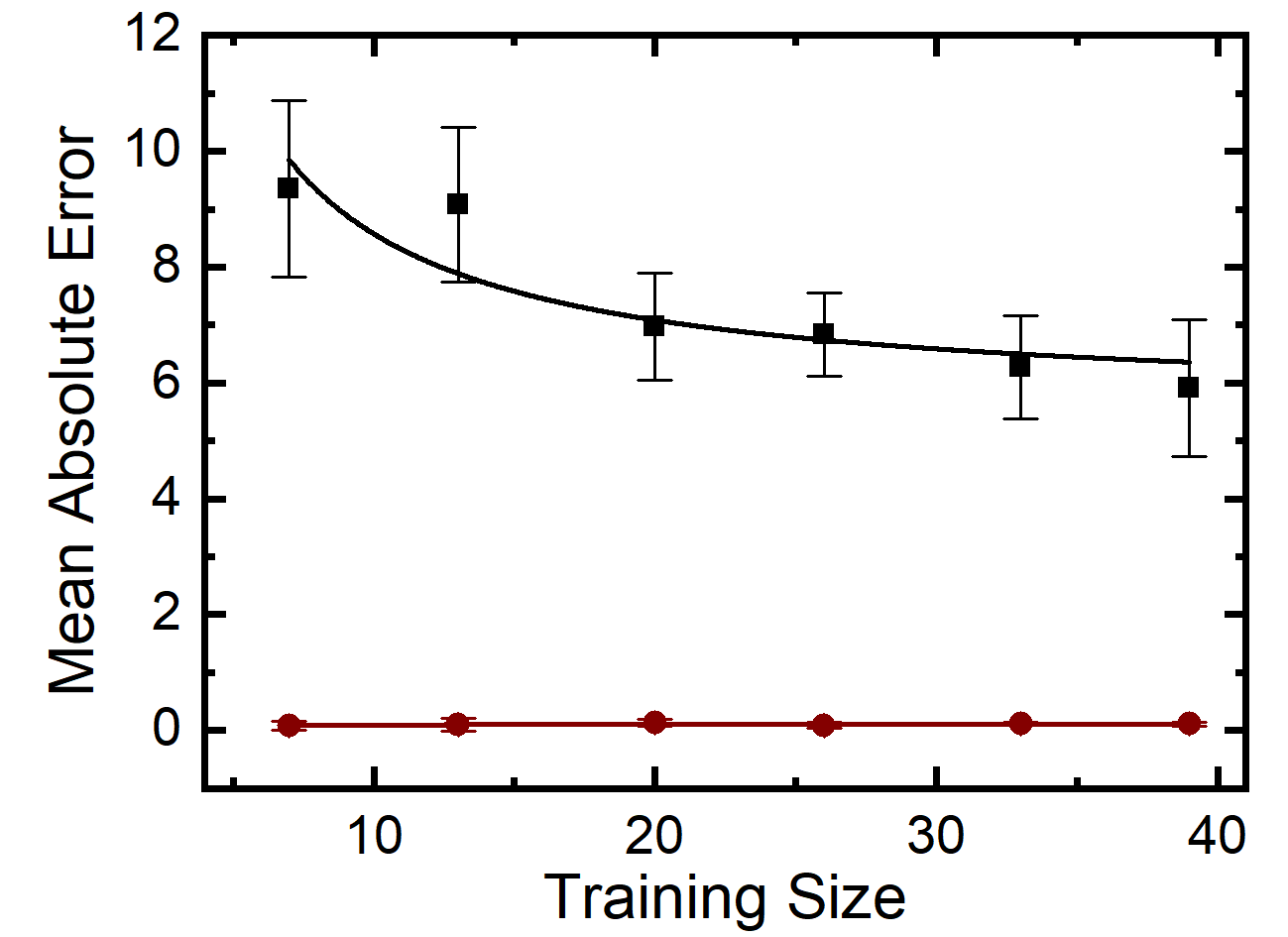


**Supplementary Figure 53.** Learning curve for cell permeability ML fits. ML fits conducted as described in the text. Number of molecules refers to the size of the training set. In total the database contains measured cell permeabilities for 63 molecules. Error bars are calculated as 1 standard deviation of the average mean absolute error.


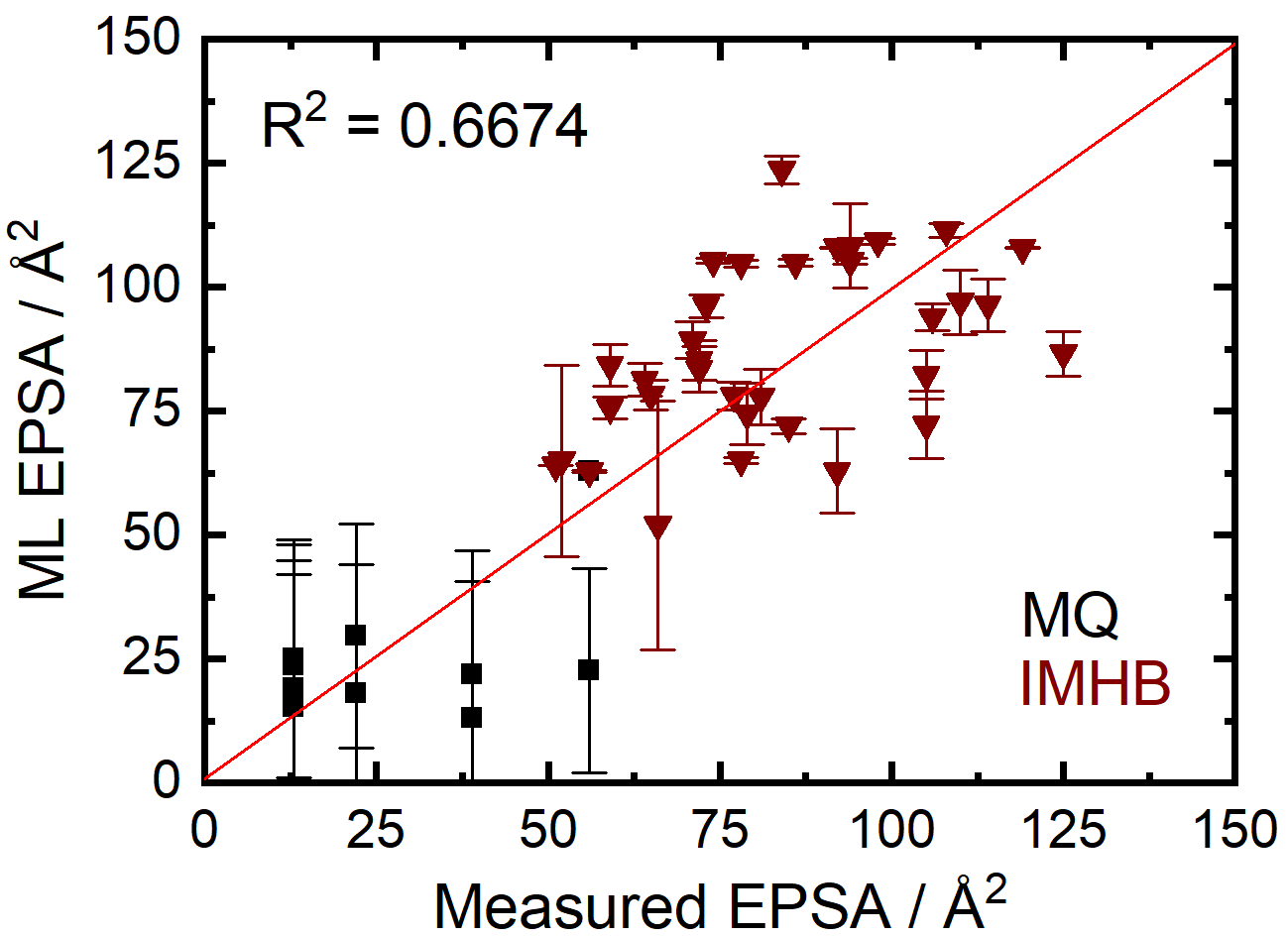


**Supplementary Figure 54.** Leave-one-out ML fit for measured EPSA. Correlation of the Random Forest model output against measured EPSA. Correlation is quite high (R^2^ = 0.65674), RMSE = 19.1, MAE = 16.7 with our current database size of measured EPSA values, 43. Error bars are calculated as 1 standard deviation of the average mean absolute error.


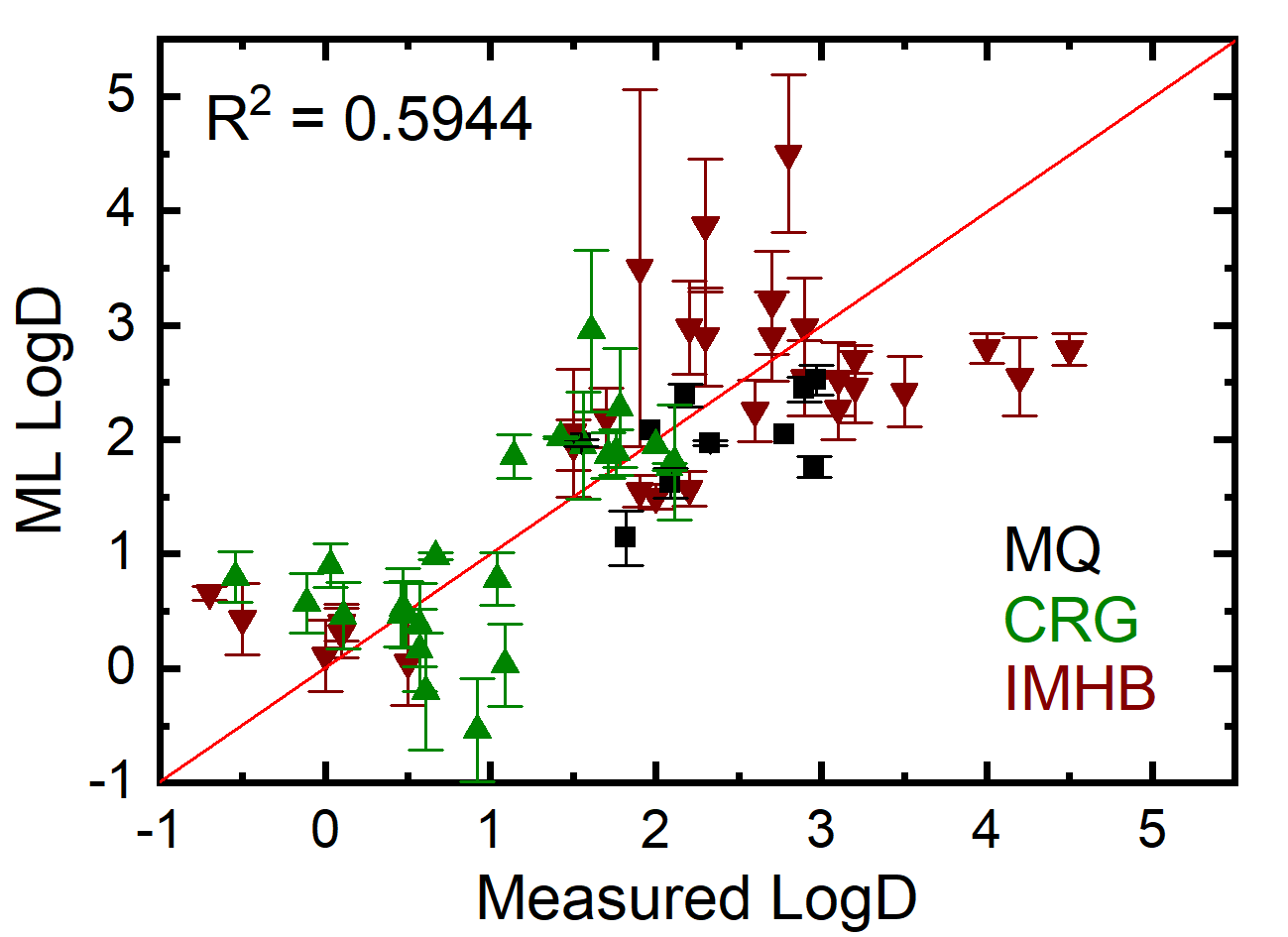


**Supplementary Figure 55.** Leave-one-out ML fit for measured LogD. Correlation of the Random Forest model output against measured LogD. Correlation is quite high (R^2^ = 0.5944), RMSE = 0.63, MAE = 0.47 with our current database size of measured LogD values, 65. Error bars are calculated as 1 standard deviation of the average mean absolute error.


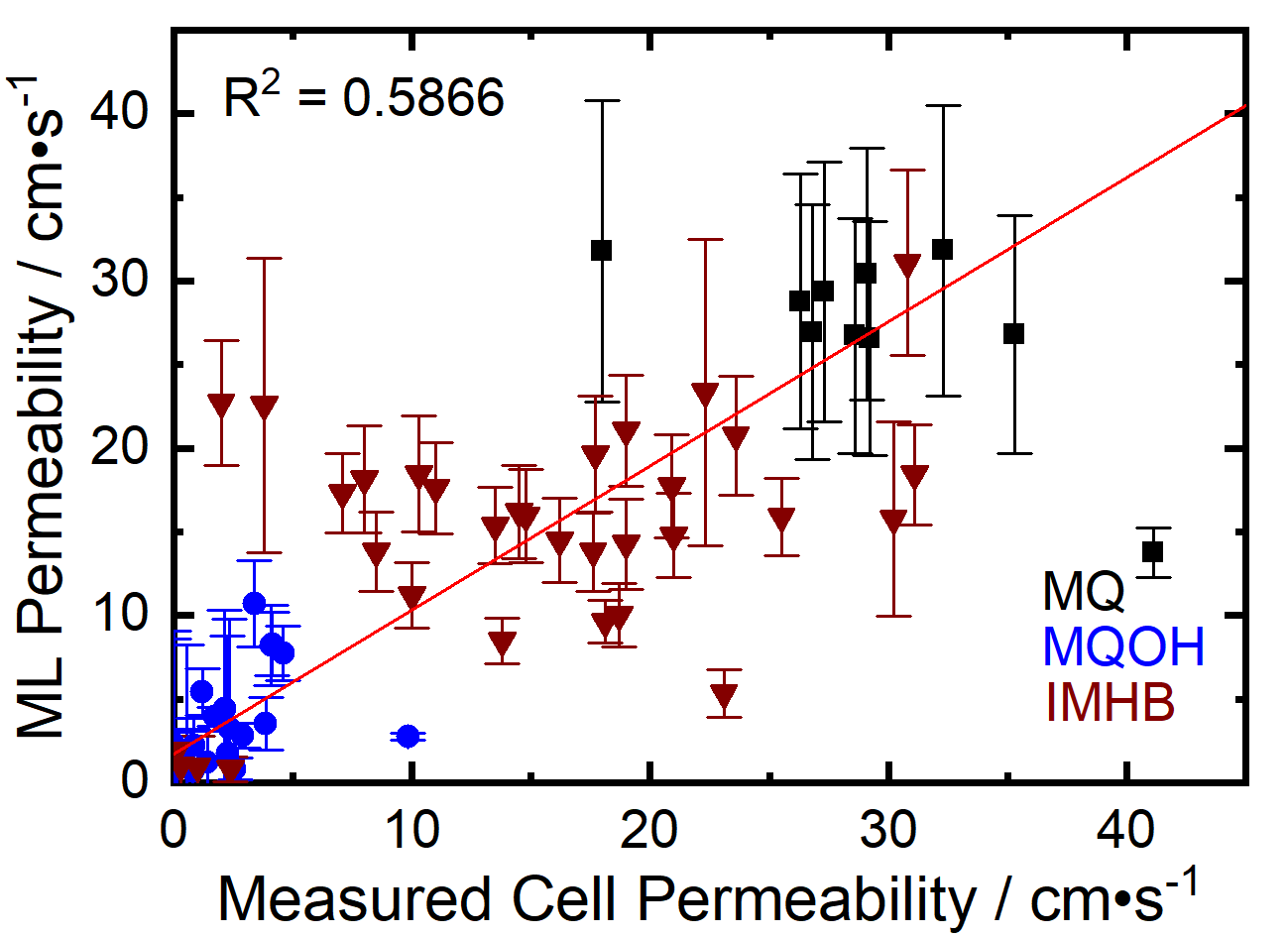


**Supplementary Figure 56.** Leave-one-out ML fit for measured cell permeability. Correlation is quite high (R^2^ = 0.5866), RMSE = 7.1, MAE = 4.6 with our current database size of measured cell permeability values, 63. Error bars are calculated as 1 standard deviation of the average mean absolute error.


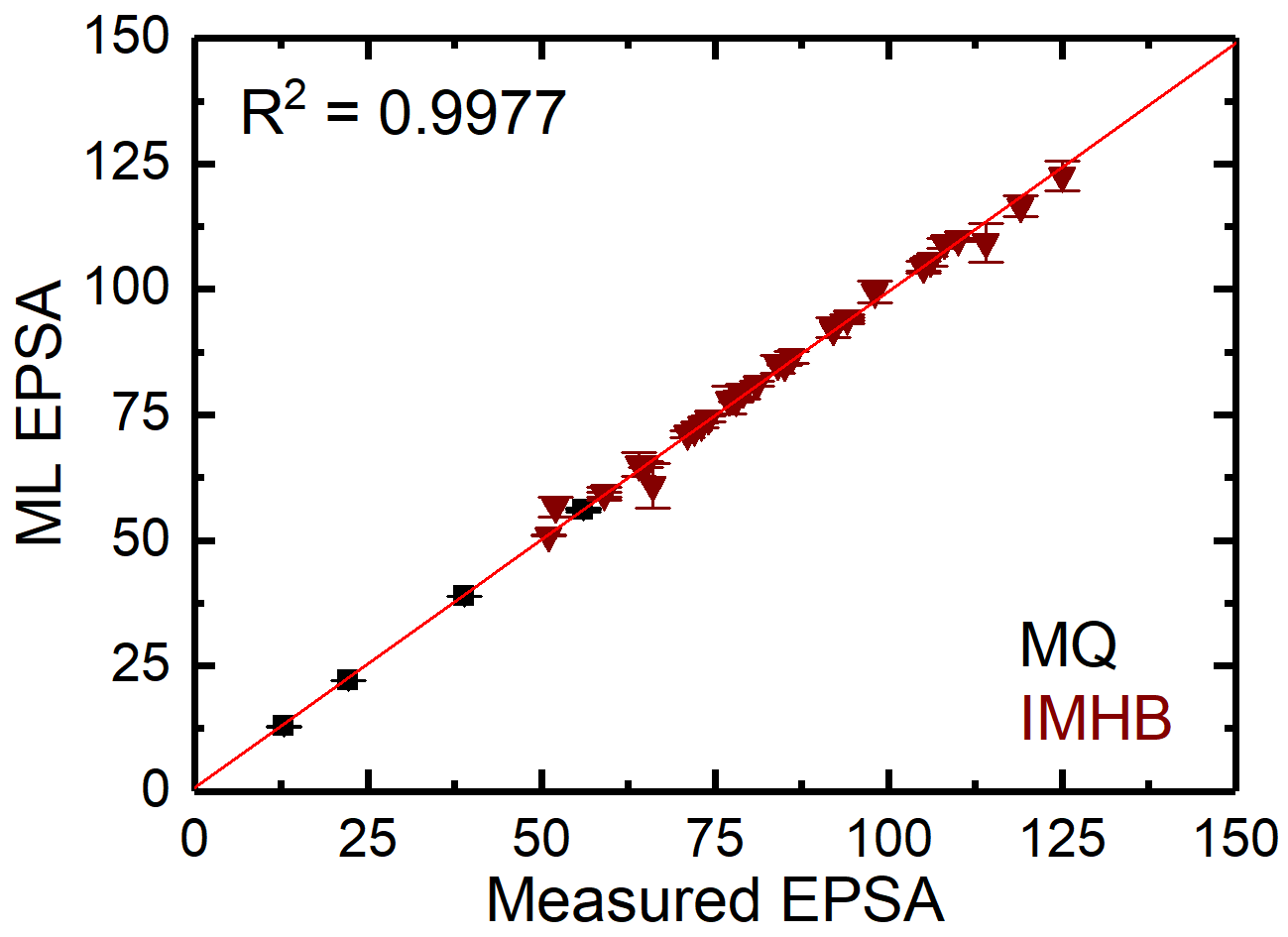


**Supplementary Figure 57.** ML training set fit of measured EPSA.

Correlation of the Random Forest model output against measured EPSA values. Error bars are calculated as 1 standard deviation of the average mean absolute error.


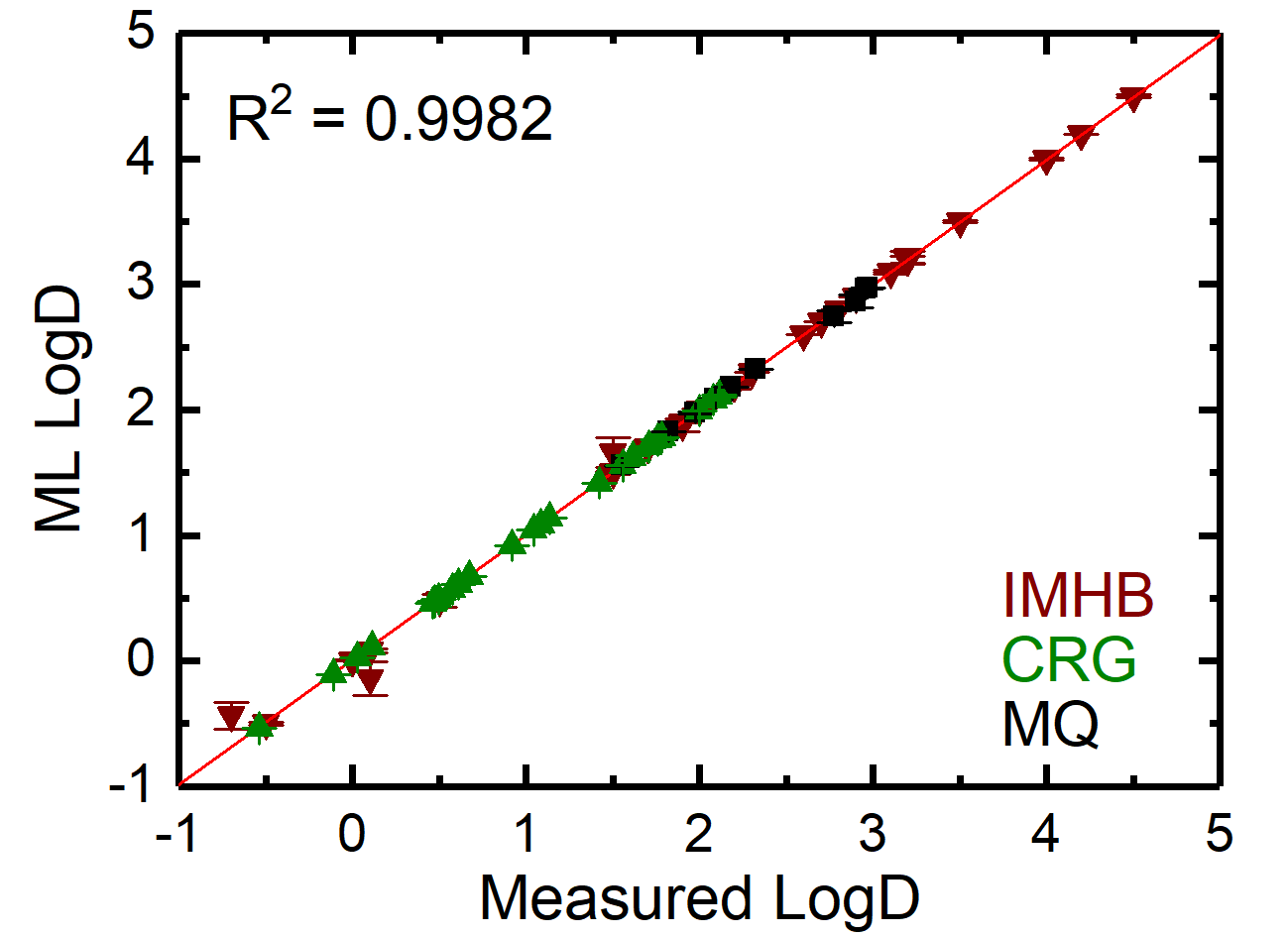


**Supplementary Figure 58.** ML training set fit of measured LogD. Correlation of the Random Forest model output against measured LogD values. Error bars are calculated as 1 standard deviation of the average mean absolute error.


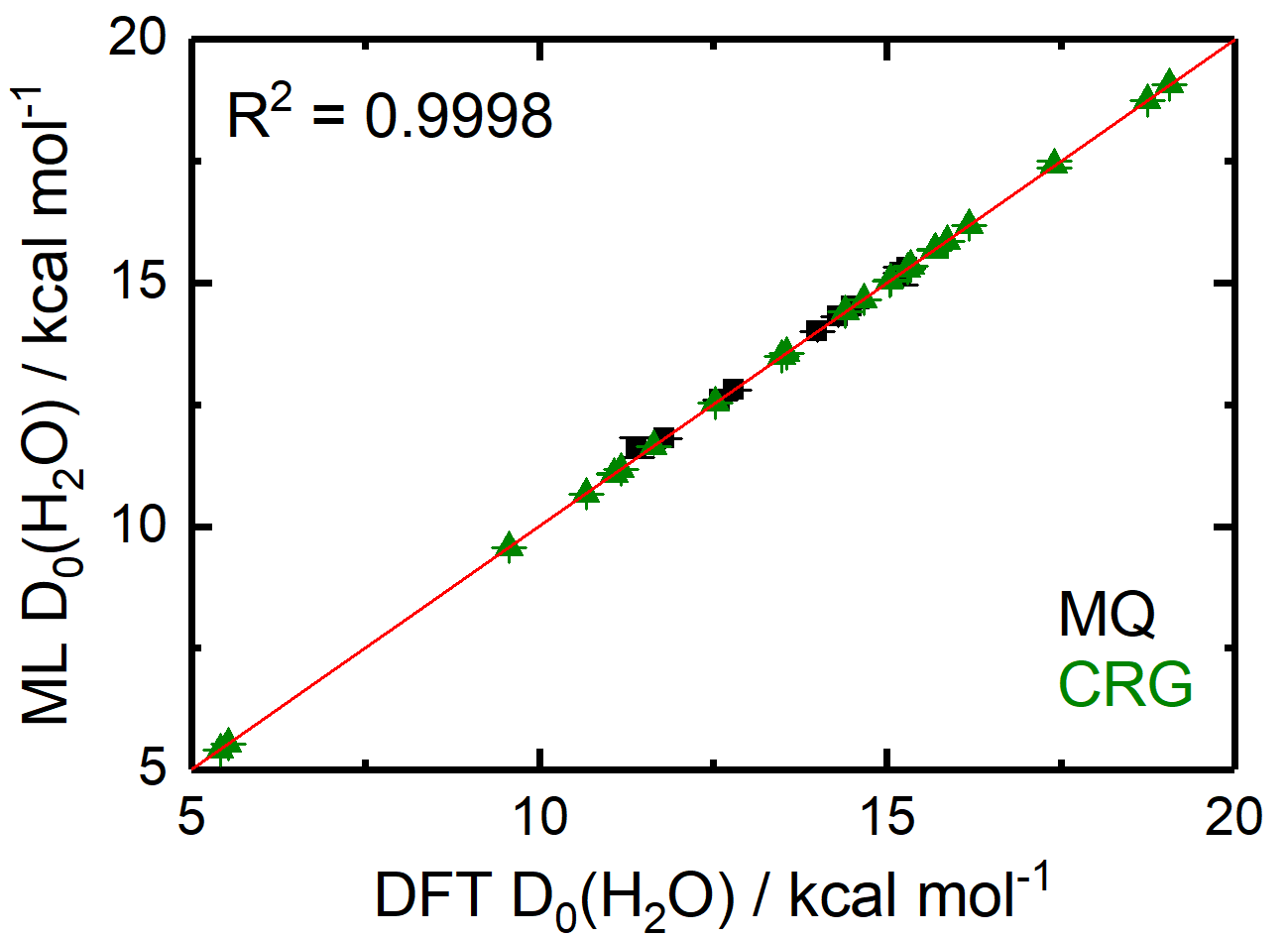


**Supplementary Figure 59.** ML training set fit of Ion-Water binding energies calculated by DFT. Correlation of the Random Forest model output against the ion-water binding energies for the 2-methylquinoline derivatives and CRG molecules as calculated at the B3LYP/6-311++G(d,p) level of theory. Error bars are calculated as 1 standard deviation of the average mean absolute error.


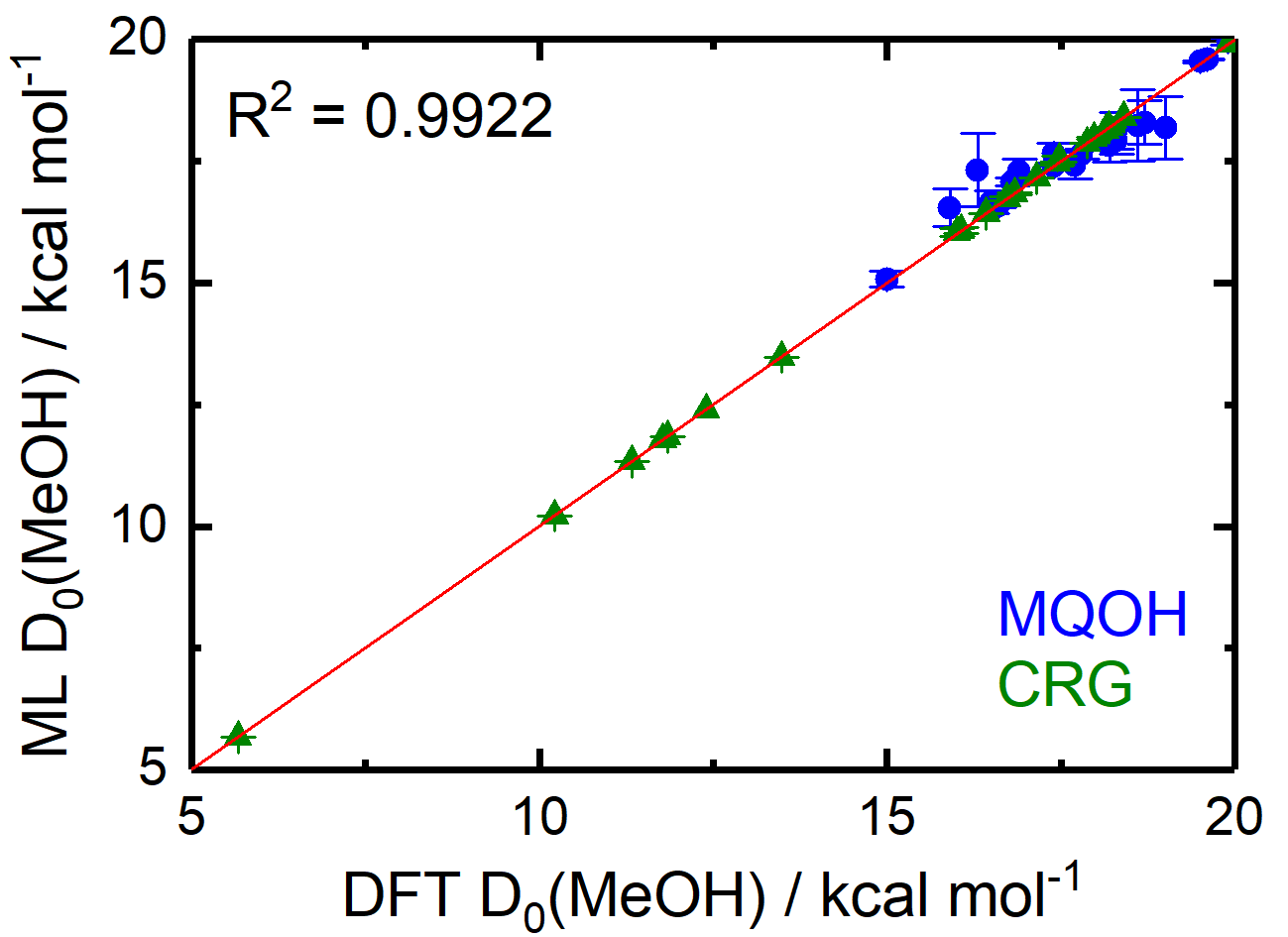


**Supplementary Figure 60.** ML training set fit of Ion-Methanol binding energies calculated by DFT. Correlation of the Random Forest model output against the ion-methanol binding energies for the 2-methylquinolin-8-ol derivatives and CRG molecules as calculated at the B3LYP/6-311++G(d,p) level of theory. Error bars are calculated as 1 standard deviation of the average mean absolute error.

**
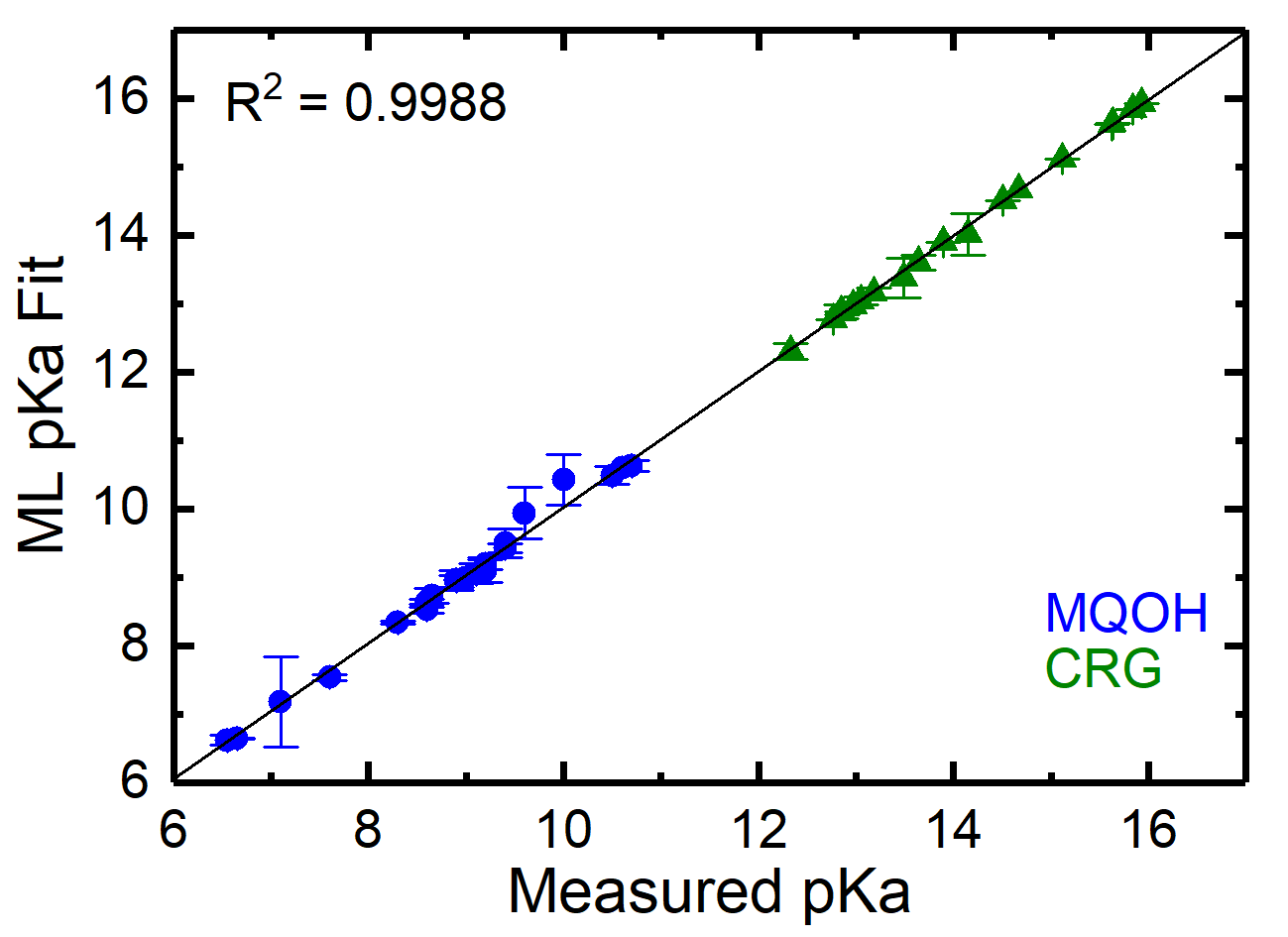
**

**Supplementary Figure 61.** ML training set fit of drug pKa values. Correlation of the Random Forest model output against the measured pKa values of the 2-methylquinolin-8-ol derivatives and CRG molecules. Error bars are calculated as 1 standard deviation of the average mean absolute error.


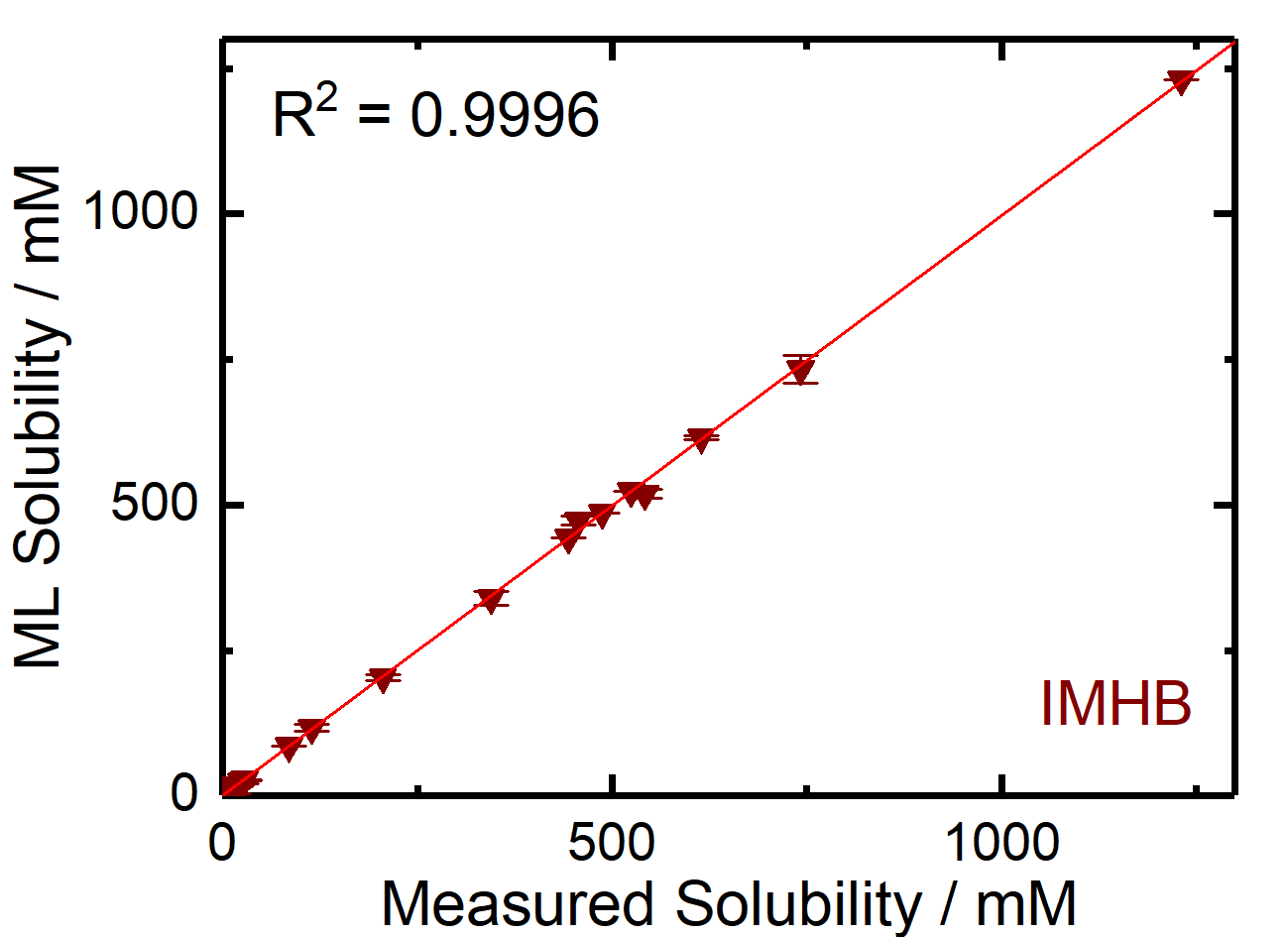


**Supplementary Figure 62.** ML training set fit of solubility for IMHB data set. Correlation of the Random Forest model output against the measured solubilities for the IMHB molecules. Error bars are calculated as 1 standard deviation of the average mean absolute error.


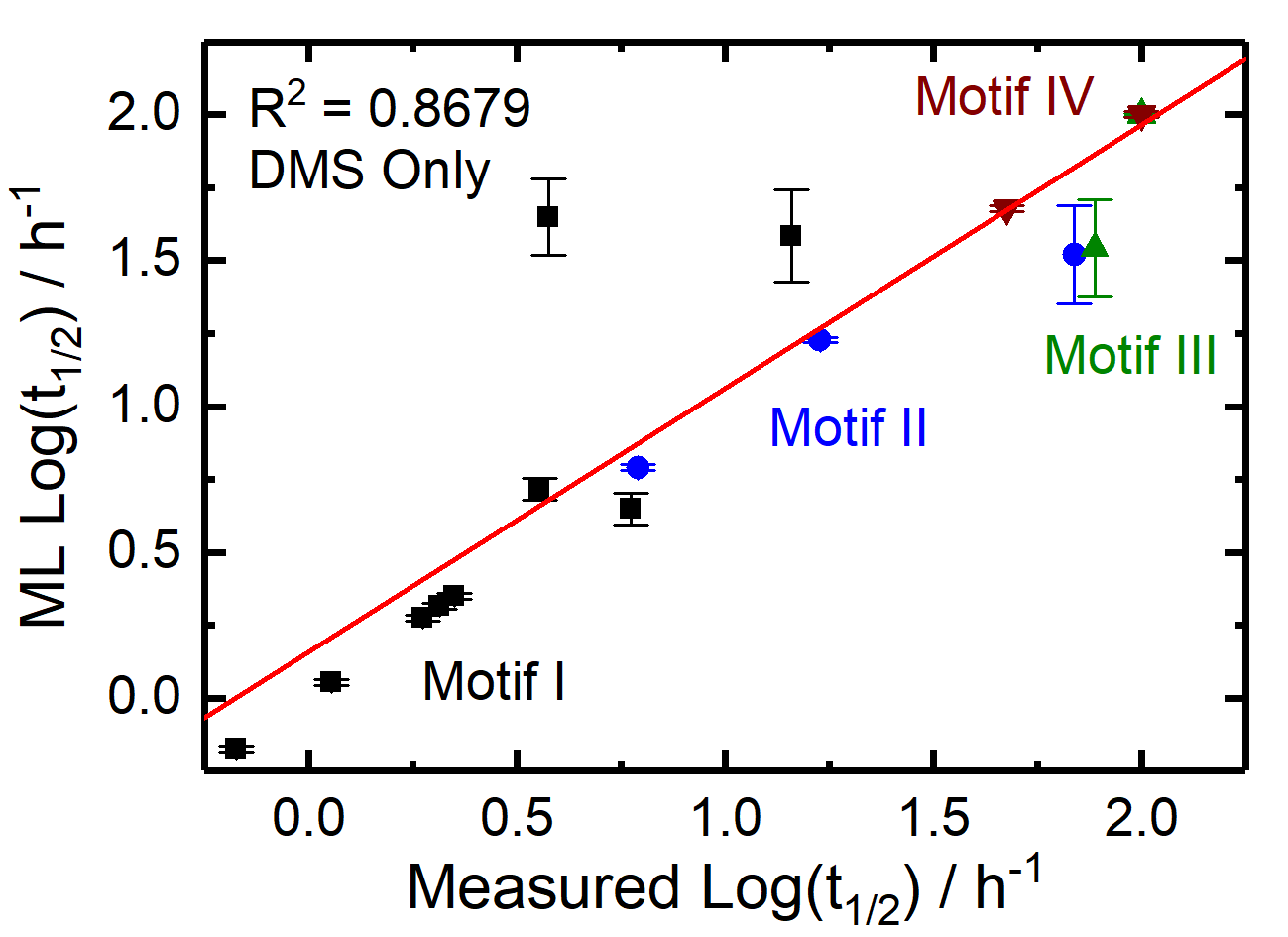


**Supplementary Figure 63.** ML training set fit of reaction half-lives for the CRG molecules reacting with GSH. Correlation of the Random Forest model output against the measured reaction rates of the CRG molecules reacting with GSH. This fit employs only the DMS data as the input. Error bars are calculated as 1 standard deviation of the average mean absolute error.


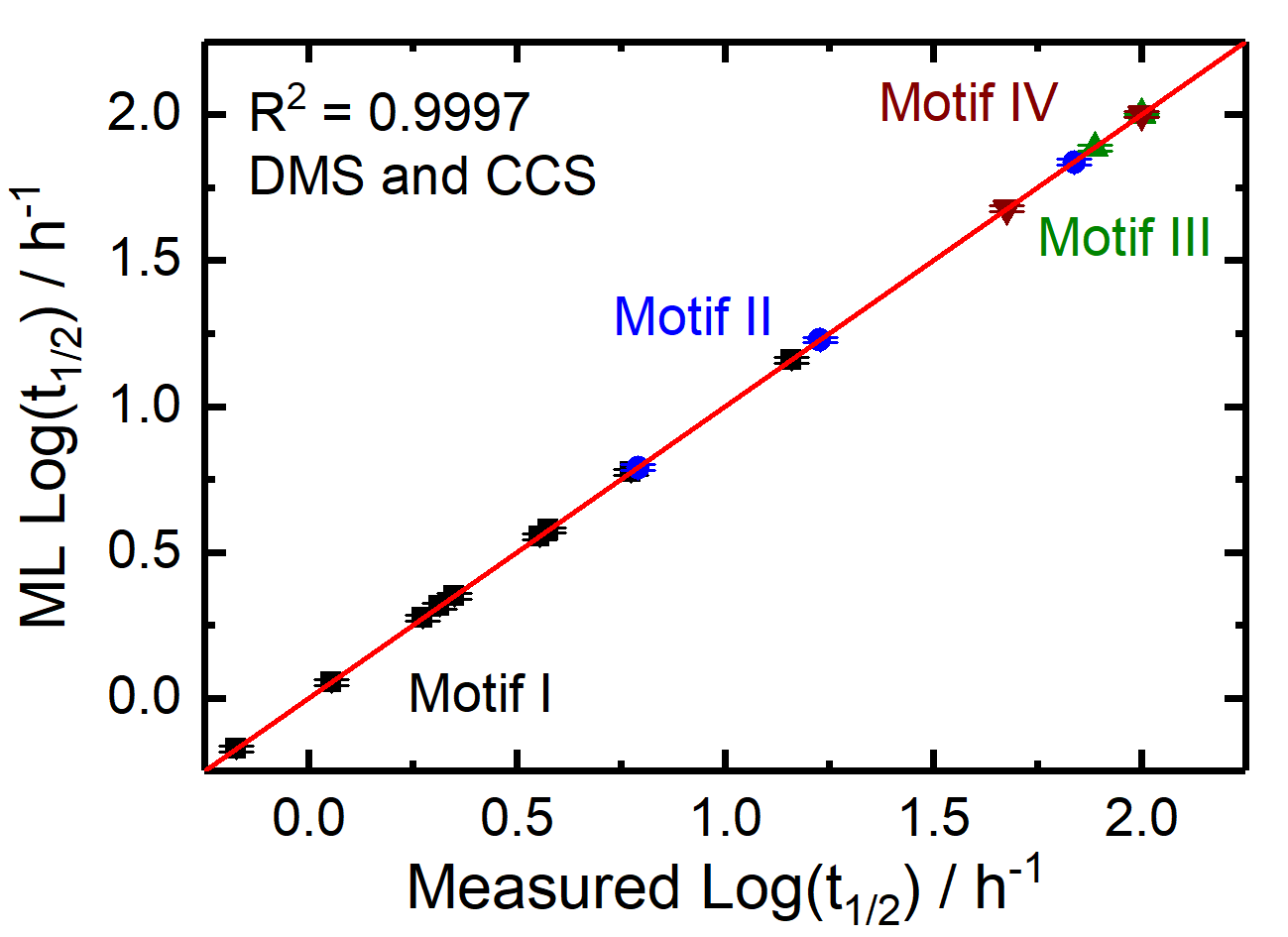


**Supplementary Figure 64.** ML training set fit of reaction half-lives for the CRG molecules reacting with GSH. Correlation of the Random Forest model output against the measured reaction rates of the CRG molecules reacting with GSH. This fit employs the DMS data and computed CCSs as inputs. Error bars are calculated as 1 standard deviation of the average mean absolute error.

Supplementary Methods - Model Testing

Random forest was chosen for the analysis in the manuscript due to data structure as well as low R^2^ in analysis. Adaboost and k nearest neighbor were also considered. Below are a series of leave-one-out correlation plots using these models with our database. Fitting errors, as described by R^2^, are similar in either of these cases to our random forest analysis, and therefore Adaboost or k-nearest neighbor could be used to reproduce patterns in our data effectively. To continue our analysis, we choose random forest not only due to low R^2^ but also due to data treatment in random forest.

**Supplementary Figure 65.** Leave one out correlation plot for Calculated CCS (Adaboost). The ML model was trained from the data as in Figure 4(B) but with Adaptive Boosting. RMSE = 3.6 Å^2^, MAE = 2.7 Å^2^

**Supplementary Figure 66.** Leave one out correlation plot for calculated CCS (kNN)

The ML model was trained from the data as in Figure 4(B) but with k-Nearest Neighbors. RMSE = 4.1 Å^2^, MAE = 2.8 Å^2^

**Supplementary Figure 67.** Leave one out correlation plot for measured EPSA (Adaboost). The ML model was trained from the data as in Figure S50 but with Adaptive Boosting. RMSE = 19.4, MAE = 16.4

**Supplementary Figure 68.** Leave one out correlation plot for measured EPSA values (kNN). The ML model was trained from the data as in Figure S50 but with k-nearest neighbors. RMSE = 18.7, MAE = 15.8

**Supplementary Figure 69.** Leave one out correlation plot for measured logD values (Adaboost). The ML model was trained from the data as in Figure S51 but with Adaptive Boosting. RMSE = 0.72, MAE = 0.53

**Supplementary Figure 70.** Leave one out correlation plot for measured logD values (kNN). The ML model was trained from the data as in Figure S51 but with k-nearest neighbors. RMSE = 0.78, MAE = 0.62

**Supplementary Figure 71.** Leave one out correlation plot for measured cell permeability (Adaboost). The ML model was trained from the data as in Figure S52 but with Adaptive Boosting. RMSE = 7.2, MAE = 5.2

**Supplementary Figure 72.** Leave one out correlation plot for measured cell permeability (kNN). The ML model was trained from the data as in Figure S52 but with k-nearest neighbors. RMSE = 7.9, MAE = 5.7

**Supplementary Figure 73.** Leave one out correlation plot for measured pKb values (Adaboost). The ML model was trained from the data as in Figure 4(D) but with Adaptive Boosting. RMSE = 1.6, MAE = 1.3

**Supplementary Figure 74.** Leave one out correlation plot for measured pKb values (kNN). The ML model was trained from the data as in Figure 4(D) but with k-nearest neighbors. RMSE = 1.3, MAE = 1.0

Supplementary Discussion - Calculation of Molecular Properties

In the manuscript we explicitly show comparison with calculated CCS values as well as measured pKb values. The expected error for our computed CCS values is < 3 % as compared with experimentally determined CCS values. Thus the error of our ML treatment is within the expected error of the CCS calculations.

Attempts to calculate pKa use either first principle quantum mechanics, chemoinformatics, or a combination of the two. In a recent study on a large data set (> 1000 pKa values) using first principle QM calculations and fitting experimental values,[^5^](#_ENREF_5) pKa was predicted for most compounds to within a mean unsigned error of < 1.0 for molecules with pKa’s in the range of 6 - 12. The error we obtain on our pKa determinations is on the order of 0.93 for 64 compounds which possess pKa values between 7.5 and 16. Admittedly, comparing our somewhat limited test set (64 compounds) with the Bochevarov study (> 1000 compounds) is optimistic, but our work does indicate the potential viability of our approach.

ML fits were also performed for logD, EPSA, and cell permeability (see Figures S50 – S52). For all molecules studied, we used measured values for these quantities which covered a range of -0.54 – 2.97 for logD, 51 – 125 for EPSA, and 0 – 41.1 for cell permeability. The mean absolute errors for the fits on each of these properties are 0.47, 17, and 4.6 respectively. EPSA[^6^](#_ENREF_6) and logD are experimental measurements devised in order to estimate cell permeability (through polarity and lipophilicity measurement, respectively). Permeability is known to strongly depend on molecular size, polarity, and lipophilicity.[^7^](#_ENREF_7) Therefore, EPSA and logD can provide qualitative measures of molecular permeability. As far as we are aware, theoretical calculations of EPSA have not yet been devised, but logD (or equivalently logP) calculations are possible. Calculation of logP follows a group based summation method[^8^](#_ENREF_8) which performs exceedingly well on the 1663 compounds tested. Standard errors were 0.58 for a logP values ranging from ‒4 to +7. This is superior to the performance of our DMS-ML database; however, we would like to (again) highlight the fact that to this point our method only employs a test set of 66 molecules. The learning curves that we calculate suggest that the performance of our method should improve with the addition of new compounds to our training set.

Direct prediction of cell permeability is much more difficult but is possible using molecular dynamics.[^9^](#_ENREF_9) In their approach the authors compare against 18 compounds for which they can provide a qualitative measure of permeability (*i.e.*, impermeable, low permeability, or high permeability). Qualitatively, the method of Bennion performs well (only 2 of 18 molecules are misclassified), but quantitative accuracy is poor. Our ability to determine cell permeability to within ca. 10 % for most molecules tested is certainly significant, especially given our relatively small test/training set.

Supplementary References

1. Hopkins WS, Marta RA, McMahon TB. Proton-Bound 3-Cyanophenylalanine Trimethylamine Clusters: Isomer-Specific Fragmentation Pathways and Evidence of Gas-Phase Zwitterions. *Journal of Physical Chemistry A* **117**, 10714-10718 (2013).

2. Campbell JL, Zhu M, Hopkins WS. Ion-Molecule Clustering in Differential Mobility Spectrometry: Lessons Learned from Tetraalkylammonium Cations and their Isomers. *J Am Soc Mass Spectrom* **25**, 1583-1591 (2014).

3. Wiberg KB, Rablen PR. COMPARISON OF ATOMIC CHARGES DERIVED VIA DIFFERENT PROCEDURES. *Journal of Computational Chemistry* **14**, 1504-1518 (1993).

4. Rappe AK, Casewit CJ, Colwell KS, Goddard WA, Skiff WM. UFF, a full periodic table force field for molecular mechanics and molecular dynamics simulations. *Journal of the American Chemical Society* **114**, 10024-10035 (1992).

5. Bochevarov AD, Watson MA, Greenwood JR, Philipp DM. Multiconformation, Density Functional Theory-Based pKa Prediction in Application to Large, Flexible Organic Molecules with Diverse Functional Groups. *Journal of Chemical Theory and Computation* **12**, 6001-6019 (2016).

6. Goetz GH*, et al.* High Throughput Method for the Indirect Detection of Intramolecular Hydrogen Bonding. *Journal of Medicinal Chemistry* **57**, 2920-2929 (2014).

7. Guimarães CRW, Mathiowetz AM, Shalaeva M, Goetz G, Liras S. Use of 3D Properties to Characterize Beyond Rule-of-5 Property Space for Passive Permeation. *Journal of Chemical Information and Modeling* **52**, 882-890 (2012).

8. Klopman G, Li J-Y, Wang S, Dimayuga M. Computer Automated log P Calculations Based on an Extended Group Contribution Approach. *Journal of Chemical Information and Computer Sciences* **34**, 752-781 (1994).

9. Bennion BJ*, et al.* Predicting a Drug’s Membrane Permeability: A Computational Model Validated With in Vitro Permeability Assay Data. *The Journal of Physical Chemistry B* **121**, 5228-5237 (2017).
