# Supplementary material for: Determining molecular properties with differential mobility spectrometry and machine learning
Source: Nat Commun. 2018 Nov 30;9:5096. doi: 10.1038/s41467-018-07616-w (PMC6269546; doi:10.1038/s41467-018-07616-w)
Supplement: Supplementary file 3 — Description of Additional Supplementary Files [file 41467_2018_7616_MOESM3_ESM.pdf]

Description of Additional Supplementary Files

File Name: Supplementary Data 1

Description: **Motif** – An overarching classification for the group. CRG refers to covalent reactive groups, 2-MQ to methylquinolines substituted at the 2 position, 2-MQ-8-OH to methylquinoline-8-ols substituted at the 2 position, and IMHB to molecules which form intramolecular hydrogen bonds.

Sub-Motif – A subclassification of Motif. Further separates the above classification into similar molecular sets.

Molecule Name – Specific molecular identifier. Each molecule name refers to only one molecular identity.

m/z – mass to charge value for the molecule of interest

Charge State – (integer) Charge of the molecule

CCS_calc – Calculated collision cross section for the molecule from MOBCAL.

Dipole Moment – Calculated dipole moment for the molecule from Gaussian 09 software package.

Temp – Temperature used in the differential mobility experiments

Modifier – Solvent modifier used in the differential mobility experiments.

SV/CV – Specific SV/CV pairs at which the molecule of interest is observed to pass through the DMS under the specific temperature and modifier conditions.

BE_H2O (kcal/mol) – Binding energy for the molecule of interest with a single water molecule, in kcal/mol. Calculations of binding energy were performed using Gaussian 09 software package.

BE_MeOH (kcal/mol) – Binding energy for the molecule of interest with a single molecule of methanol, in kcal/mol. Calculations of binding energy were performed using Gaussian 09 software package.

BE_EtOH (kcal/mol) – Binding energy for the molecule of interest with a single molecule of ethanol, in kcal/mol. Calculations of binding energy were performed using Gaussian 09 software package.

BE_IPA (kcal/mol) – Binding energy for the molecule of interest with a single molecule of isopropanol, in kcal/mol. Calculations of binding energy were performed using Gaussian 09 software package.

BE_ACE (kcal/mol) – Binding energy for the molecule of interest with a single molecule of acetone, in kcal/mol. Calculations of binding energy were performed using Gaussian 09 software package.

BE_ACN (kcal/mol) – Binding energy for the molecule of interest with a single molecule of acetonitrile, in kcal/mol. Calculations of binding energy were performed using Gaussian 09 software package.

pKa – Measured pKa values for the molecule of interest.

pKb – Measured pKb values for the molecule of interest.

Solubility – Measured Solubility for the molecule of interest

elogD – Measured octanol-water logarithmic distribution coefficients

SFlogD – Measured octanol-water logarithmic distribution coefficients by shake flask

cPFlogD – Combined approach for measuring octanol-water logarithmic distribution coefficients. Uses shake flask as well as a chromatographic approach to measure logD.

Cell Permeability – Measured cell permeability

G48105 log(t1/2) – Measured drug half-life
G47689 log(t1/2) – Measured drug half-life
Polar Surface Area – Experimental polar surface area (EPSA) measurements
Use of database for machine learning:
The database can be read directly using the Orange program but will omit entries in which all data columns are not filled. The database is currently in its infancy and so only those columns which have all molecular entries filled are used for machine learning. i.e. for CCS learning only CCS_calc, m/z, Charge State and the DMS information is included (Modifier, Temp, SV/CV). For the manuscript the Orange program is *not* used directly but instead custom written Python programs which call the relevant machine learning algorithms included in the Orange package. This is an important distinction as to apply machine learning properly to our database it needs to be divided properly. To be clear all machine learning for the accompanying manuscript was conducted by first subdividing our database in to test and training sets based upon Molecule name. This is important due to repetitions in our database which yield false learning opportunities for the ML algorithms.

File Name: Supplementary Data 2
Description: This data file contains gaussian optimized geometries for all drug candidates studied. The format for all geometries is:

ATOM TYPE X Y Z
