# Supplementary material for: Determining molecular properties with differential mobility spectrometry and machine learning
Source: Nat Commun. 2018 Nov 30;9:5096. doi: 10.1038/s41467-018-07616-w (PMC6269546; doi:10.1038/s41467-018-07616-w)
Supplement: Supplementary file 5 — Supplementary Dataset 2 [file 41467_2018_7616_MOESM5_ESM.docx]

Optimized IMHB Geometries

Molecular geometries are provided as: atomic number, X, Y, Z

Molecule: A1a


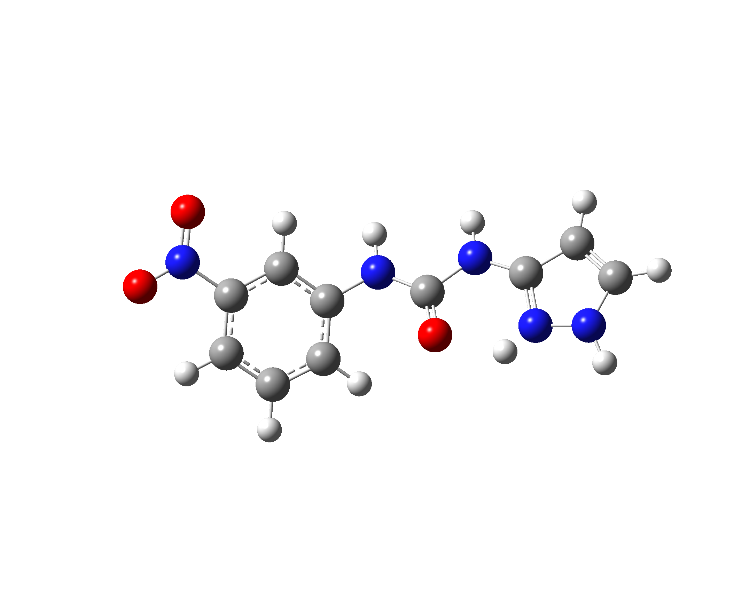


6 5.785626 0.429220 0.497719

6 4.864906 1.256829 -0.147872

6 3.635510 0.533529 -0.178524

7 5.143866 -0.720646 0.907147

1 5.528451 -1.621662 1.158879

1 6.836501 0.606353 0.724756

1 5.038614 2.247767 -0.531373

7 3.863490 -0.723993 0.373999

7 2.409368 0.903521 -0.664635

1 2.344859 1.754729 -1.222889

6 1.261246 0.084583 -0.378967

8 1.445173 -0.954243 0.233460

7 0.072111 0.556489 -0.846231

1 -0.011588 1.469766 -1.286714

6 -1.149595 -0.132404 -0.542509

6 -2.253133 0.647371 -0.184505

6 -1.241766 -1.525517 -0.637531

6 -3.459973 -0.001938 0.074939

1 -2.177947 1.736960 -0.103674

6 -2.459318 -2.144511 -0.365921

1 -0.375427 -2.126596 -0.921279

6 -3.576386 -1.390854 -0.008547

1 -2.539433 -3.233546 -0.436159

1 -4.534051 -1.886626 0.202664

7 -4.629204 0.800348 0.448451

8 -4.505370 2.012636 0.444593

8 -5.652045 0.211343 0.740562

1 3.127838 -1.352898 0.766060

Molecule: A1b


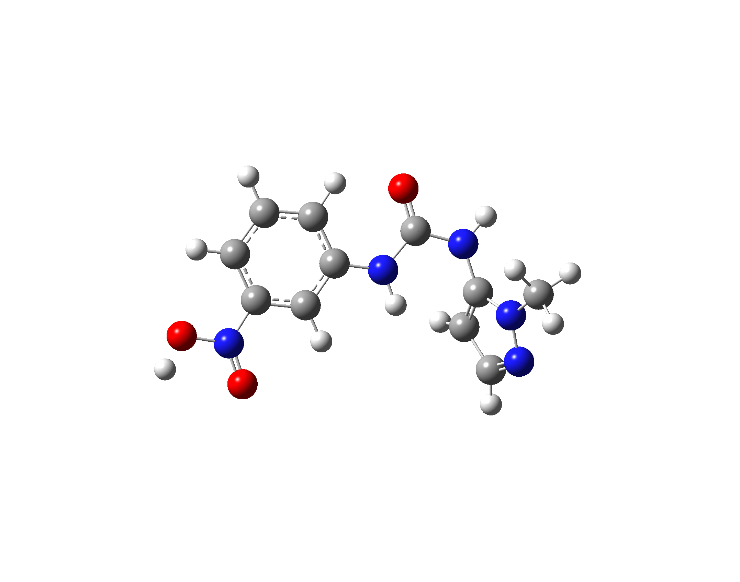


6 3.579697 -2.189113 -0.895139

6 2.926629 -1.038206 -1.409204

6 3.102616 -0.049001 -0.426622

1 3.683279 -3.160966 -1.350268

1 2.445983 -0.939378 -2.357157

7 4.119746 -1.917451 0.324198

7 3.819850 -0.637230 0.614802

6 4.344673 -0.013235 1.817557

1 3.597036 0.669742 2.259678

1 4.609030 -0.785890 2.572220

1 5.267887 0.558987 1.591003

7 2.663497 1.278692 -0.354609

1 3.331050 2.024376 -0.575919

6 1.322496 1.632784 -0.315153

8 0.983130 2.793384 -0.376984

7 0.398927 0.568263 -0.152390

1 0.758681 -0.390906 -0.148457

6 -0.976776 0.746896 -0.060761

6 -1.599189 2.024198 -0.004610

6 -1.779907 -0.399808 -0.013106

6 -2.977867 2.132032 0.089571

1 -0.986308 2.945210 -0.035251

6 -3.182047 -0.259876 0.086429

1 -1.323105 -1.392922 -0.055439

6 -3.794411 1.002942 0.136828

1 -3.438136 3.129681 0.128639

1 -4.879482 1.126890 0.211657

7 -3.936144 -1.442054 0.130617

8 -3.536699 -2.565627 0.095603

8 -5.270857 -1.242180 0.223667

1 -5.727122 -2.158650 0.247945

Molecule: A2a


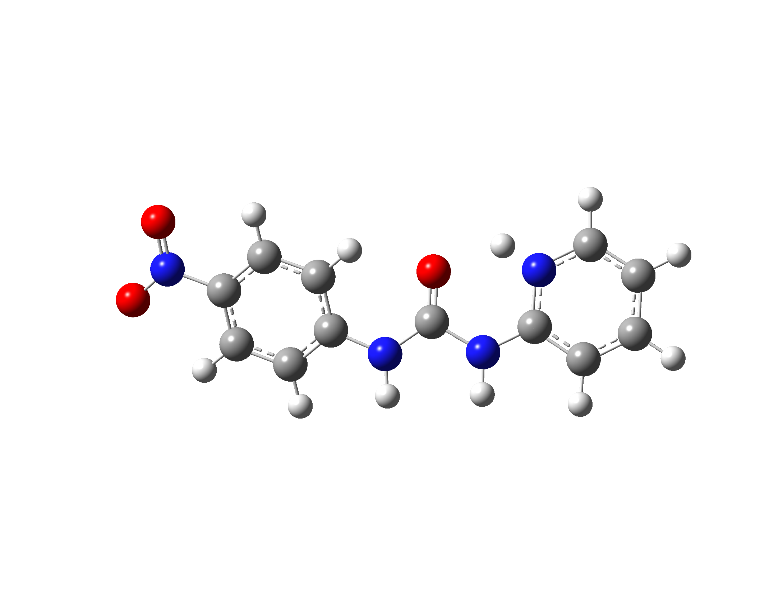


6 4.802972 1.636933 -0.351637

6 3.585461 -0.374880 0.055584

6 4.808086 -1.085260 0.165060

6 6.006269 -0.416231 0.012560

6 6.003384 0.966882 -0.250588

1 4.735491 2.721076 -0.555951

1 4.794604 -2.160377 0.369609

1 6.961860 -0.955212 0.094982

1 6.951439 1.505808 -0.373784

7 3.606187 0.976364 -0.199806

7 2.378124 -1.042320 0.196926

1 2.423415 -2.043966 0.400655

6 1.103145 -0.384244 0.116524

8 1.077115 0.820113 -0.056569

7 0.022667 -1.209692 0.254210

1 0.133568 -2.219261 0.325155

6 -1.319264 -0.718027 0.161107

6 -2.295409 -1.637534 -0.254654

6 -1.653881 0.595206 0.499624

6 -3.620328 -1.228943 -0.338265

1 -2.030098 -2.660852 -0.518583

6 -2.983343 1.000211 0.411026

1 -0.898322 1.310037 0.832770

6 -3.954291 0.088485 -0.007516

1 -4.395612 -1.937409 -0.664165

1 -3.257941 2.031993 0.672914

7 -5.349386 0.519138 -0.101594

8 -5.603540 1.678940 0.168351

8 -6.175535 -0.306527 -0.445139

1 2.697324 1.507942 -0.277837

Molecule: A2b


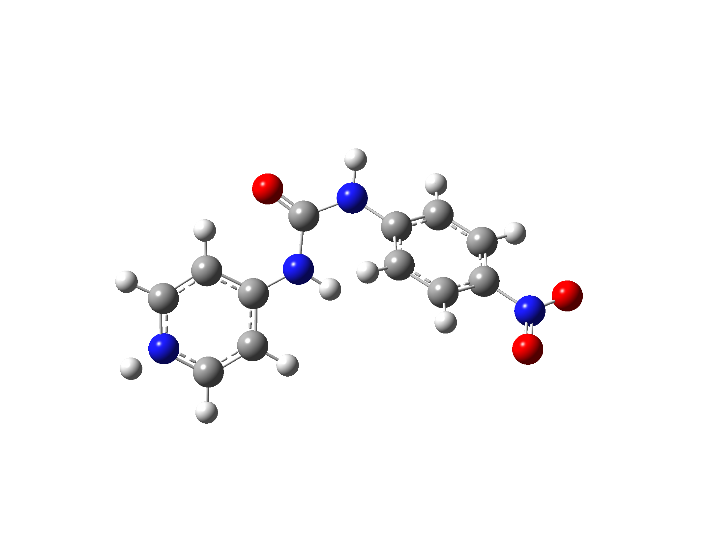


6 -4.076569 -2.237978 -0.542453

6 -2.858011 -1.598171 -0.580103

6 -2.742360 -0.238814 -0.164674

6 -3.925130 0.403426 0.291952

6 -5.120389 -0.276043 0.312016

1 -4.196317 -3.288982 -0.862451

1 -1.977387 -2.142563 -0.937543

1 -3.901738 1.455616 0.630302

1 -6.057043 0.198211 0.657959

7 -5.200275 -1.585667 -0.102412

7 -1.509895 0.350741 -0.214012

1 -0.715972 -0.205153 -0.554156

6 -1.273447 1.738421 0.088335

8 -2.187631 2.503047 0.283271

7 0.040863 2.151848 0.070440

1 0.190846 3.166772 0.148627

6 1.188970 1.313135 0.085724

6 2.253050 1.643667 -0.764002

6 1.264757 0.212093 0.949003

6 3.391658 0.845295 -0.771559

1 2.193061 2.516734 -1.417931

6 2.402775 -0.586310 0.936527

1 0.465256 0.003044 1.658764

6 3.449938 -0.272002 0.066268

1 4.235306 1.092567 -1.433065

1 2.479739 -1.447963 1.613826

7 4.634040 -1.131217 0.036305

8 5.609106 -0.733734 -0.572674

8 4.566168 -2.201214 0.614974

1 -6.101485 -2.073326 -0.083346

Molecule: A3a


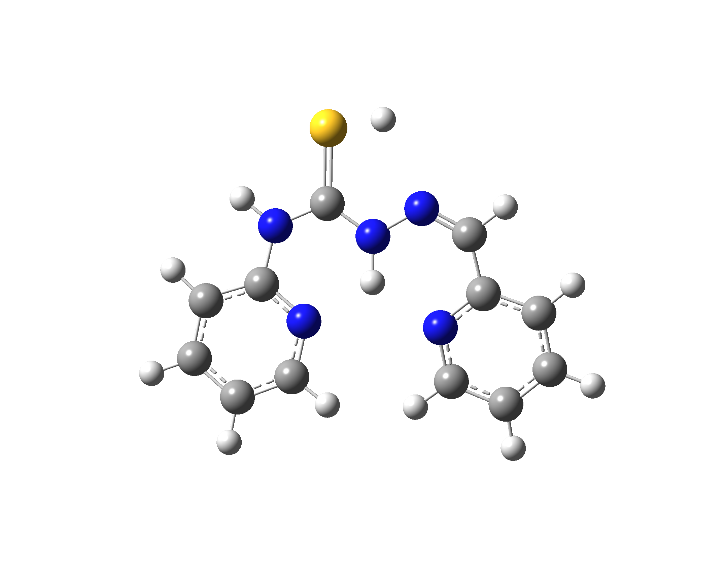


6 3.847662 -2.006109 -0.314209

6 4.691085 -0.929868 -0.065347

6 4.141751 0.338902 0.141797

6 2.750495 0.475279 0.093432

6 2.459193 -1.785850 -0.341478

1 4.247229 -3.006953 -0.487285

1 5.776591 -1.068740 -0.033582

1 4.787344 1.196125 0.334049

1 1.752015 -2.601470 -0.539878

7 1.923167 -0.574734 -0.136829

6 2.113560 1.789045 0.270539

1 2.803302 2.647218 0.442354

7 0.865224 2.127566 0.246776

7 -0.126023 1.192441 0.065066

1 0.112716 0.123765 0.072532

6 -1.366565 1.686642 -0.060118

16 -1.746708 3.435852 -0.106039

7 -2.455903 0.868935 -0.186900

1 -3.378752 1.293017 -0.358471

6 -2.454867 -0.546690 -0.058973

6 -3.664281 -1.251295 -0.184466

6 -1.244751 -2.477592 0.358918

6 -3.616533 -2.634392 -0.022349

1 -4.605147 -0.748399 -0.397606

6 -2.400541 -3.259244 0.256233

1 -0.259802 -2.911039 0.582281

1 -4.534240 -3.226723 -0.111364

1 -2.347190 -4.340404 0.393525

7 -1.268097 -1.140069 0.196619

1 -0.512566 3.958045 0.063072

Molecule: A3b


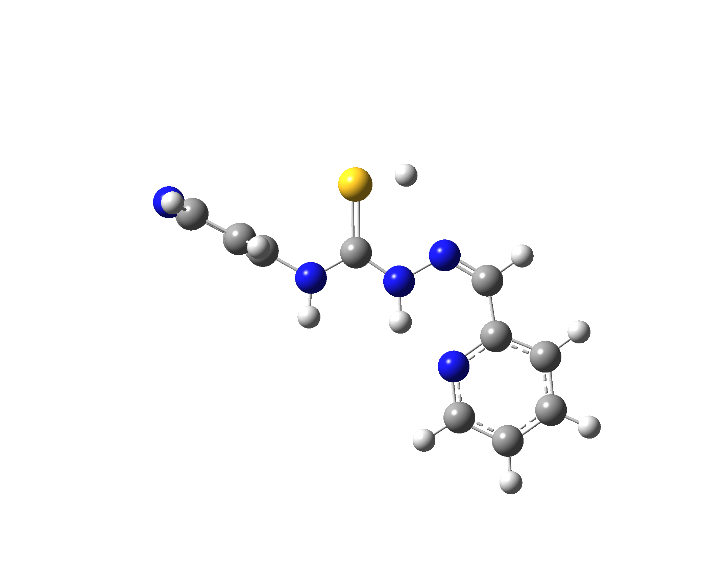


6 5.179118 -1.798626 0.000152

6 5.765525 -0.539185 -0.000015

6 4.951356 0.598914 -0.000049

6 3.565689 0.423943 0.000088

6 3.776206 -1.894040 0.000242

1 5.788997 -2.705486 0.000167

1 6.856059 -0.432031 -0.000123

1 5.394855 1.595820 -0.000218

1 3.265015 -2.867320 0.000315

7 2.989669 -0.808817 0.000162

6 2.648704 1.576695 -0.000117

1 3.119820 2.584912 -0.000384

7 1.353696 1.598306 -0.000105

7 0.649956 0.416891 0.000085

1 1.205294 -0.492095 0.000266

6 -0.701082 0.514038 -0.000042

16 -1.596386 2.050420 -0.000517

7 -1.399749 -0.642433 0.000154

1 -0.939830 -1.555135 0.000173

6 -2.842221 -0.652706 0.000134

6 -3.526429 -0.680300 1.214921

6 -4.928220 -0.736358 -1.160063

6 -4.928317 -0.734911 1.160276

1 -3.009317 -0.662950 2.172090

1 -5.540138 -0.762825 -2.075610

1 -5.540280 -0.760261 2.075824

7 -5.606963 -0.760417 0.000104

6 -3.526344 -0.681842 -1.214667

1 -3.009139 -0.665699 -2.171808

1 -0.559331 2.917885 -0.000385

Molecule: A4a


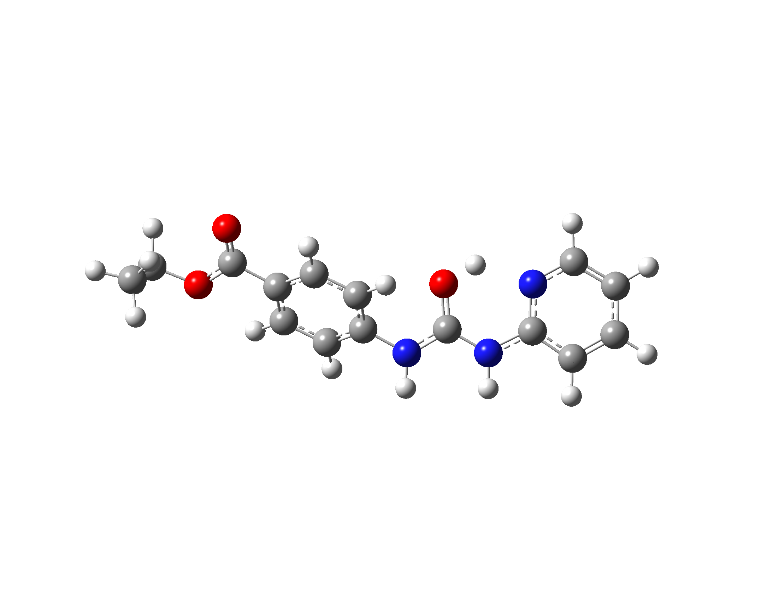


6 -4.398483 0.624230 -0.107339

8 -4.738457 1.730820 0.224395

8 -5.232552 -0.297563 -0.627167

6 -6.618841 0.062650 -0.746498

1 -6.977057 -0.724413 -1.438497

1 -6.702033 1.056625 -1.222410

6 -7.280478 0.004505 0.615274

1 -7.084098 -0.949302 1.120918

1 -6.915440 0.806401 1.273655

1 -8.369354 0.120137 0.535475

6 -3.016044 0.089670 -0.002587

6 -2.103021 0.823287 0.757987

6 -2.631190 -1.091354 -0.638674

6 -0.789629 0.378458 0.892367

1 -2.423515 1.752652 1.248534

6 -1.320549 -1.547518 -0.517654

1 -3.350480 -1.663561 -1.236997

6 -0.419138 -0.802500 0.248364

1 -0.080484 0.948813 1.492265

1 -1.016087 -2.463993 -1.021335

7 0.918388 -1.325554 0.390888

6 2.003988 -0.551737 0.162967

1 1.006194 -2.320162 0.599257

7 3.265942 -1.084974 0.293021

8 1.753072 0.688446 -0.162783

6 4.466593 -0.350486 0.078513

1 3.379500 -2.065648 0.567197

6 5.709938 -0.977440 0.238463

7 4.325331 0.946167 -0.278830

6 6.847759 -0.201749 0.010306

1 5.799982 -2.023155 0.527668

6 5.433476 1.681741 -0.496393

6 6.714759 1.136330 -0.359875

1 7.843638 -0.648343 0.123000

1 5.263981 2.731322 -0.786191

1 7.597533 1.755352 -0.542362

1 2.608755 1.295660 -0.362617

Molecule: A4b


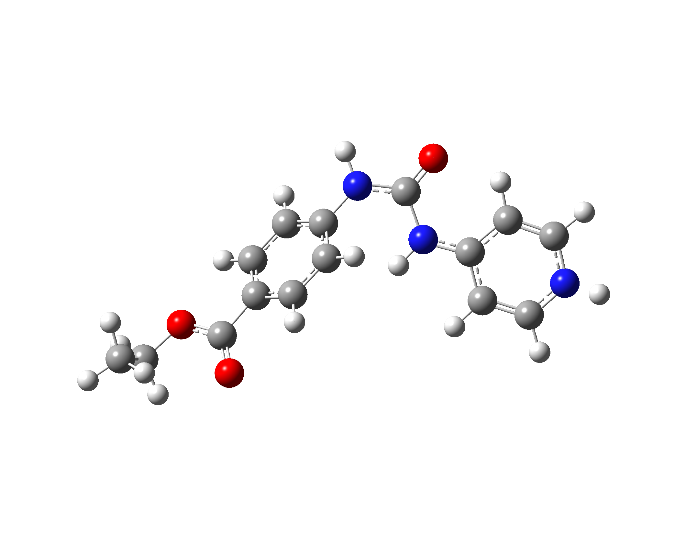


6 -3.858859 -0.771596 0.003880

8 -3.952478 -1.877711 0.472681

8 -4.865929 -0.127158 -0.617343

6 -6.142719 -0.784555 -0.658992

1 -6.011048 -1.826471 -1.001781

1 -6.656800 -0.187328 -1.437342

6 -6.808714 -0.691342 0.699059

1 -6.819112 0.340190 1.074213

1 -7.848371 -1.041086 0.659359

1 -6.285417 -1.306497 1.445433

6 -2.631096 0.059927 0.022126

6 -1.659023 -0.253212 0.975905

6 -2.440598 1.111161 -0.873730

6 -0.475201 0.474377 1.024552

1 -1.838683 -1.069548 1.686550

6 -1.255322 1.843280 -0.839743

1 -3.215831 1.364564 -1.606583

6 -0.277322 1.509233 0.102105

1 0.264123 0.263243 1.794985

1 -1.098166 2.664143 -1.540825

7 0.928718 2.272178 0.120852

6 2.201861 1.758920 0.108876

1 0.848653 3.291297 0.213091

7 2.314705 0.345897 -0.161563

8 3.184636 2.444653 0.266455

6 3.490857 -0.344083 -0.132712

1 1.459550 -0.155276 -0.435987

6 4.746663 0.214057 0.234682

6 5.878430 -0.564909 0.238341

1 4.827499 1.280233 0.516163

6 4.630911 -2.469050 -0.461610

1 2.530631 -2.210426 -0.766672

1 6.867593 -0.158744 0.515877

1 4.645674 -3.540775 -0.728466

6 3.471341 -1.728543 -0.479885

7 5.826657 -1.896476 -0.107622

1 6.682041 -2.458984 -0.101150

Molecule: A4c


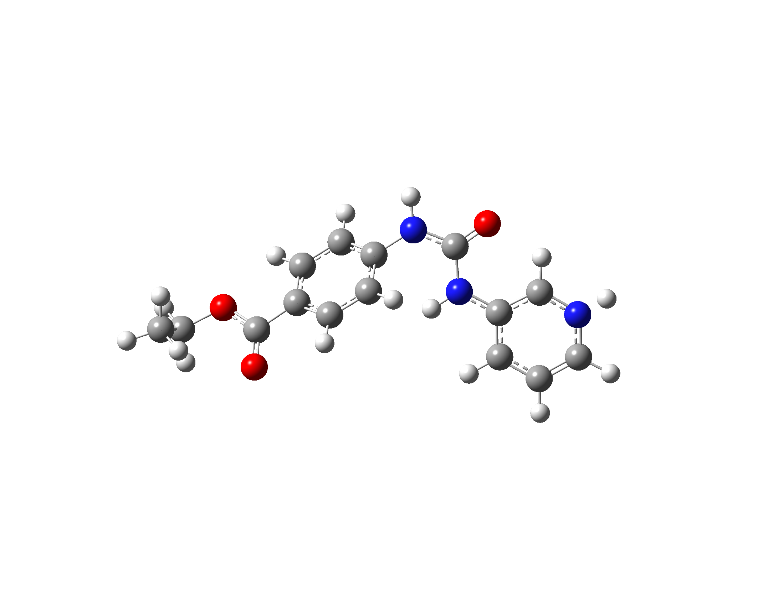


6 -3.896612 -0.760016 0.009001

8 -4.015407 -1.852418 0.503554

8 -4.884089 -0.116054 -0.643786

6 -6.169730 -0.755364 -0.689880

1 -6.048428 -1.807429 -1.004376

1 -6.662360 -0.170506 -1.491119

6 -6.855882 -0.618270 0.654405

1 -7.899265 -0.955616 0.606953

1 -6.352592 -1.220547 1.424694

1 -6.858356 0.422656 1.002652

6 -2.656411 0.052598 0.025957

6 -1.699146 -0.259271 0.995042

6 -2.440210 1.085659 -0.885149

6 -0.505027 0.451238 1.044674

1 -1.898435 -1.061148 1.716908

6 -1.244586 1.800624 -0.850096

1 -3.203207 1.338318 -1.630915

6 -0.281765 1.467728 0.107737

1 0.222986 0.242146 1.826498

1 -1.067137 2.606727 -1.563087

7 0.934461 2.213972 0.127938

6 2.198956 1.681151 0.133179

1 0.870370 3.235259 0.200480

7 2.309650 0.272573 -0.091008

8 3.189348 2.366777 0.281518

6 3.514246 -0.396615 -0.112245

1 1.459385 -0.257306 -0.310486

6 3.520471 -1.784745 -0.410095

6 4.715803 -2.479142 -0.428334

1 2.579321 -2.304533 -0.626273

1 4.828664 1.310170 0.405475

6 5.918456 -1.816920 -0.154062

1 4.734975 -3.554196 -0.658505

1 6.885027 -2.343377 -0.163730

7 5.906163 -0.490689 0.132675

6 4.755648 0.225988 0.162132

1 6.793063 -0.005911 0.333696

Molecule: A5a


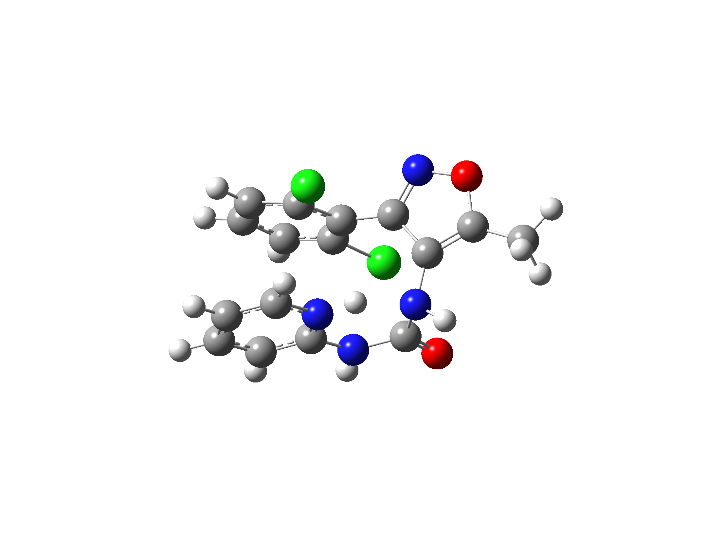


6 -0.247649 -0.147641 1.985314

6 0.034627 -0.908320 0.850575

6 -0.976288 -1.736781 0.368304

6 -2.195949 -1.872760 1.017595

6 -2.424443 -1.133671 2.179251

6 -1.457455 -0.250051 2.659852

1 -2.955757 -2.566016 0.647562

1 -3.366603 -1.255101 2.722662

1 -1.642895 0.328492 3.570556

17 0.935624 0.943058 2.518954

17 -0.691837 -2.570480 -1.085954

6 1.367167 -0.910170 0.270396

6 1.995227 0.041128 -0.634881

8 3.437469 -1.579479 -0.056555

6 3.293621 -0.421992 -0.783990

7 2.256272 -1.849971 0.581193

6 4.471972 0.034130 -1.511201

1 5.381581 -0.554312 -1.268643

1 4.345578 -0.042210 -2.606544

1 4.722964 1.086761 -1.277837

7 1.381861 1.141263 -1.291124

1 2.017924 1.535289 -2.017611

6 0.982385 2.188475 -0.368110

8 1.709161 2.983589 0.151949

7 -0.416407 2.295065 -0.132535

1 -0.667502 3.005036 0.579936

6 -1.446508 1.572505 -0.704090

6 -2.783386 1.754159 -0.267787

6 -2.192330 -0.029563 -2.309668

6 -3.798074 1.030383 -0.861486

1 -2.997977 2.461974 0.538258

6 -3.499765 0.126480 -1.898206

1 -1.894660 -0.720315 -3.118762

1 -4.838213 1.155117 -0.530214

1 -4.301059 -0.448423 -2.374963

7 -1.179545 0.685910 -1.720870

1 -0.154970 0.523256 -1.996399

Molecule: A5b


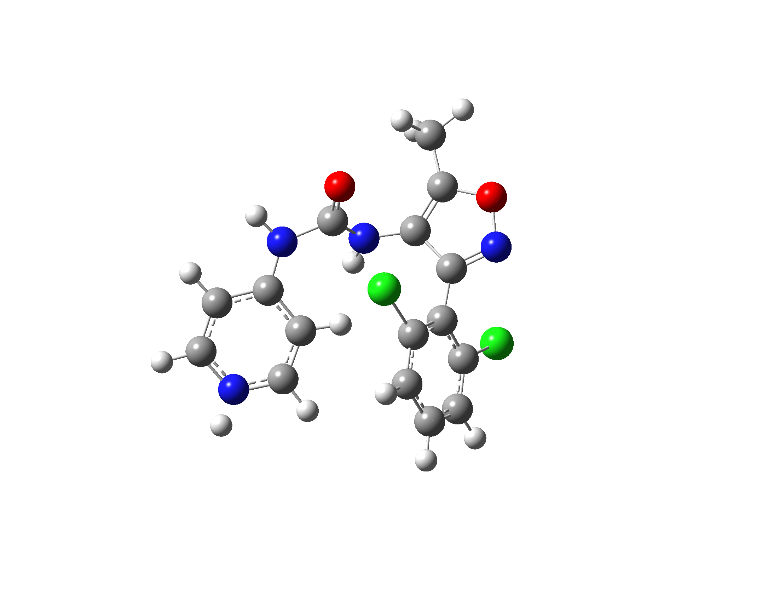


6 0.256190 -1.253986 1.267981

6 1.288839 -1.153197 0.334869

6 1.644554 -2.314615 -0.353193

6 1.029753 -3.536702 -0.100339

6 0.031099 -3.606032 0.869268

6 -0.370276 -2.458417 1.555496

1 1.340222 -4.438581 -0.636545

1 -0.431186 -4.567923 1.105192

1 -1.138965 -2.520595 2.328268

17 -0.262809 0.158571 2.069251

17 2.856630 -2.220852 -1.532043

6 2.003613 0.094774 0.134947

6 1.475394 1.346318 -0.396553

8 3.626835 1.578971 0.183503

6 2.533145 2.237499 -0.327039

7 3.280338 0.275972 0.453234

6 2.704302 3.652196 -0.629665

1 3.712425 4.035065 -0.383452

1 2.529205 3.872725 -1.698597

1 1.980450 4.269060 -0.055617

7 0.163854 1.572941 -0.839413

1 -0.174892 1.026122 -1.618737

6 -0.658082 2.292029 -0.008789

8 -0.333674 3.114353 0.806367

7 -2.102507 2.167469 -0.232687

1 -2.595506 3.046756 0.002830

6 -2.835257 1.045217 -0.406732

6 -2.292946 -0.257723 -0.604892

6 -5.049841 0.043084 -0.605916

6 -3.118935 -1.340048 -0.791488

1 -1.212488 -0.426877 -0.584797

1 -6.152880 0.095326 -0.615637

1 -2.721432 -2.359535 -0.941375

6 -4.265460 1.150847 -0.412501

1 -4.737882 2.127236 -0.257746

7 -4.485807 -1.199040 -0.796667

1 -5.085014 -2.015453 -0.939280

Molecule: A6a


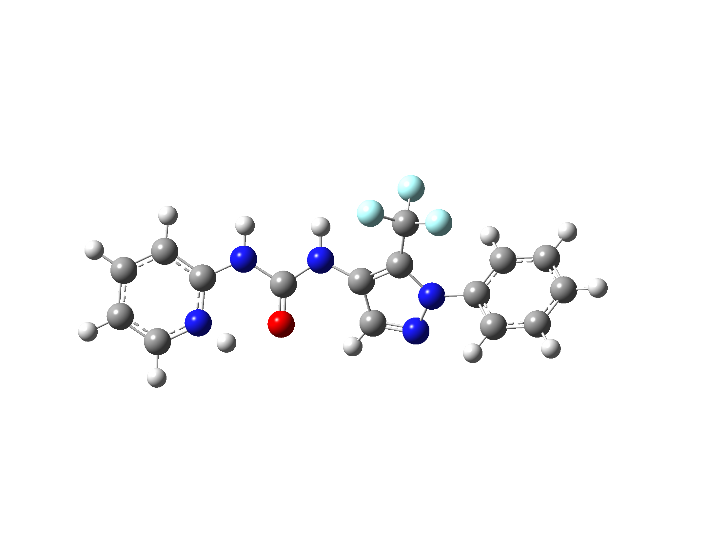


6 4.568441 0.155213 -0.816469

6 3.763969 -0.723021 -0.083296

6 4.295032 -1.582194 0.882136

6 5.664085 -1.533936 1.134458

6 6.477990 -0.649468 0.428114

6 5.933195 0.188428 -0.545812

1 4.143534 0.794134 -1.590073

1 3.651340 -2.278683 1.421357

1 6.097908 -2.193716 1.888186

1 7.549636 -0.614353 0.635076

1 6.578091 0.871993 -1.101598

7 2.354462 -0.748709 -0.337677

6 1.454889 0.296076 -0.217846

6 0.436742 -1.589860 -0.948689

6 0.202065 -0.221389 -0.628222

1 -0.266371 -2.337259 -1.282362

7 1.753235 -1.873154 -0.758771

6 1.766275 1.657586 0.284168

9 1.921851 2.555213 -0.689991

9 2.859583 1.752173 1.032087

9 0.787146 2.160042 1.040905

7 -1.004987 0.491484 -0.717993

1 -0.949648 1.491179 -0.892089

6 -2.170472 -0.136382 -0.383020

8 -2.303114 -1.325515 -0.157086

7 -3.328490 0.715219 -0.354062

1 -3.229302 1.715036 -0.549720

6 -4.597866 0.252318 -0.056689

6 -5.699450 1.147628 -0.042042

6 -6.061945 -1.539183 0.530391

6 -6.961096 0.677575 0.259084

1 -5.539741 2.206060 -0.269695

6 -7.145645 -0.688339 0.550002

1 -6.142434 -2.618774 0.750323

1 -7.823355 1.359830 0.274358

1 -8.145586 -1.068888 0.790535

7 -4.801536 -1.077679 0.230728

1 -3.982410 -1.745739 0.222206

Molecule: A6b


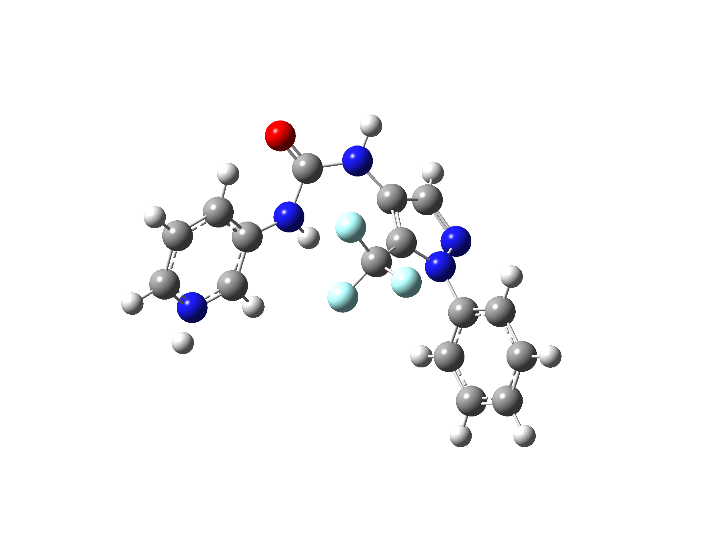


6 4.006799 -0.005058 -0.798620

6 3.156894 0.070464 0.307762

6 3.268524 1.084858 1.261982

6 4.242561 2.063023 1.079823

6 5.086915 2.015743 -0.029739

6 4.972611 0.984964 -0.962119

1 3.922485 -0.824143 -1.514879

1 2.617721 1.103684 2.134149

1 4.348269 2.864476 1.812735

1 5.847784 2.788132 -0.164937

1 5.644773 0.947722 -1.822707

7 2.143357 -0.931005 0.473243

6 0.898262 -0.970302 -0.135290

6 1.217659 -2.757366 1.213011

6 0.269848 -2.149275 0.336858

1 1.129472 -3.678880 1.768695

7 2.339381 -1.990387 1.269165

6 0.400332 0.065947 -1.070345

9 -0.618386 -0.336149 -1.829090

9 1.298795 0.542615 -1.923977

9 -0.074857 1.148004 -0.439435

7 -0.980142 -2.684854 0.014470

1 -1.001028 -3.609099 -0.433458

6 -2.167171 -1.983988 0.053980

8 -3.189654 -2.401984 -0.432880

7 -2.126911 -0.738893 0.768641

1 -1.391489 -0.609847 1.466213

6 -3.001612 0.290383 0.515808

6 -4.024845 0.238451 -0.471575

6 -4.833796 1.336467 -0.669580

1 -4.172052 -0.674210 -1.076766

6 -4.654580 2.501181 0.095904

1 -5.628021 1.314602 -1.430267

1 -5.288672 3.389022 -0.050265

7 -3.684129 2.546092 1.034962

6 -2.864955 1.484428 1.264155

1 -2.094337 1.589171 2.039029

1 -3.549205 3.398809 1.592572

Molecule: B1a


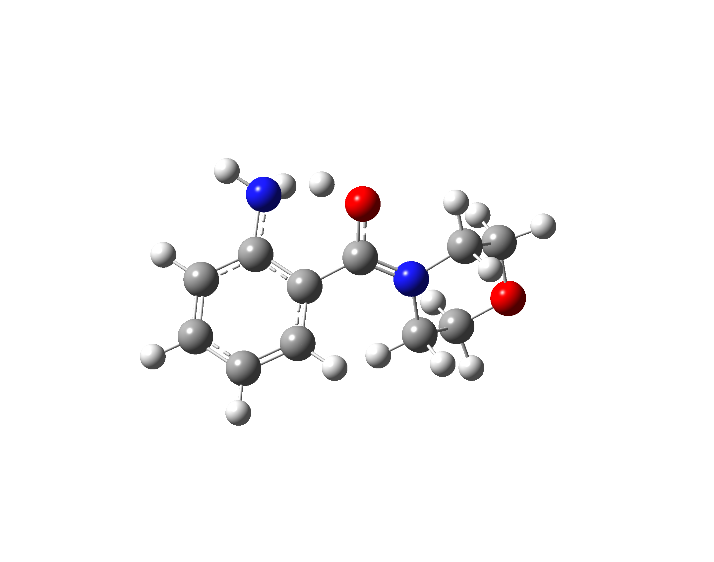


6 1.226800 -0.018737 -0.227572

6 2.216957 0.739859 0.431775

6 3.495599 0.214426 0.631808

6 3.795504 -1.060894 0.161859

6 2.830195 -1.807331 -0.519829

6 1.558966 -1.286817 -0.722002

1 4.260835 0.798925 1.148126

1 3.082648 -2.799904 -0.904836

1 0.829326 -1.862797 -1.297465

6 -0.070372 0.597017 -0.494850

8 -0.117829 1.881876 -0.824748

6 -1.503533 -1.357780 0.000969

6 -2.482515 0.740997 -0.895634

6 -2.613896 -1.324776 1.076260

6 -3.533445 0.657353 0.225771

7 -1.262053 0.016769 -0.474640

8 -3.762491 -0.696512 0.555772

1 -0.586136 -1.820803 0.424316

1 -1.835106 -1.983122 -0.865878

1 -2.973499 -2.347970 1.315862

1 -2.280960 -0.829510 2.005253

1 -3.244312 1.236289 1.120453

1 -4.530789 1.000917 -0.122711

1 -2.868631 0.253689 -1.825828

1 -2.268730 1.804654 -1.155479

1 4.797107 -1.479131 0.319384

7 1.890091 2.089925 0.776689

1 2.702144 2.680593 0.949111

1 1.243534 2.172279 1.557800

1 0.797289 2.386216 -0.641979

Molecule: B1b


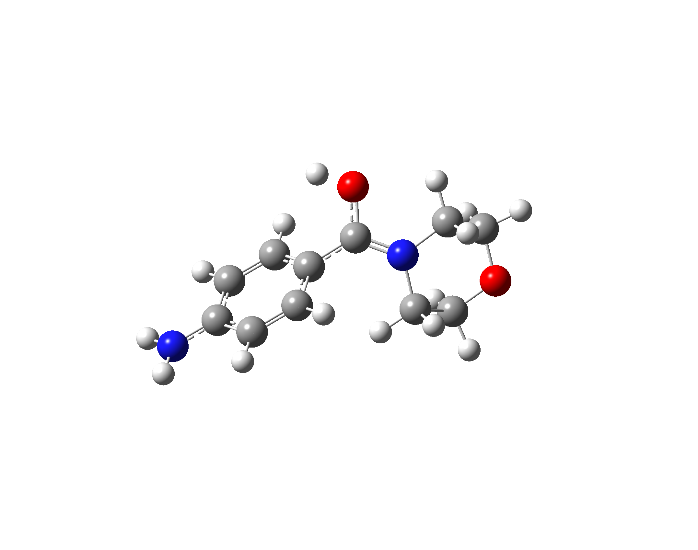


6 0.922319 0.486728 -0.150754

6 1.523359 -0.298988 -1.152390

6 2.830749 -0.706790 -1.030286

6 3.584536 -0.336766 0.119586

6 2.973501 0.460629 1.129037

6 1.667740 0.864376 0.983952

1 0.949627 -0.572328 -2.043204

1 3.297221 -1.306873 -1.811022

1 3.545598 0.745735 2.011701

1 1.195454 1.474561 1.760841

7 4.874711 -0.739995 0.252419

1 5.426342 -0.485847 1.050025

6 -0.438705 0.949799 -0.277317

8 -0.690910 2.268014 -0.222298

6 -2.870552 0.814720 -0.615808

6 -1.516842 -1.253510 -0.377739

6 -3.794887 0.259812 0.485192

6 -2.516361 -1.698019 0.711474

7 -1.527188 0.221030 -0.452743

8 -3.787433 -1.151429 0.438466

1 -2.843554 1.928471 -0.586370

1 -3.257395 0.526461 -1.625463

1 -3.517302 0.621529 1.491025

1 -4.859371 0.509546 0.289443

1 -2.700693 -2.792496 0.671514

1 -2.179583 -1.423322 1.726704

1 -0.499487 -1.648678 -0.159572

1 -1.829531 -1.659537 -1.372232

1 5.327767 -1.295138 -0.448528

1 0.149850 2.826042 -0.231621

Molecule: B2a


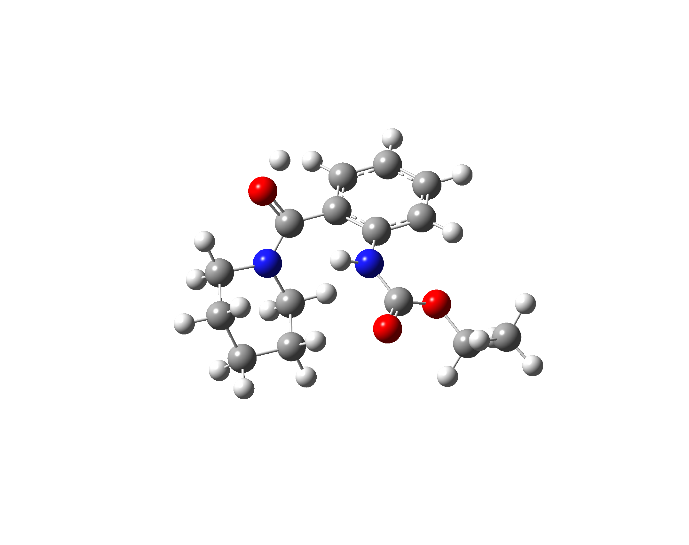


6 1.481096 -1.971298 1.080955

6 2.856721 -2.552090 0.746026

6 3.518183 -1.748583 -0.375750

6 3.623596 -0.265144 0.004224

1 1.026462 -2.519497 1.933771

1 3.502400 -2.571556 1.646154

1 2.752248 -3.615414 0.440054

1 4.534009 -2.146106 -0.588797

1 2.954904 -1.872126 -1.322533

1 4.285537 -0.127447 0.894106

1 4.083179 0.322438 -0.826058

1 0.781934 -2.132240 0.234435

7 2.278389 0.252460 0.349938

6 1.708246 1.236420 -0.312851

8 2.497195 1.785691 -1.250400

6 0.376345 1.784246 -0.110031

6 -0.788848 1.066338 -0.455151

6 0.268116 3.069532 0.450542

6 -2.048747 1.645330 -0.218657

6 -0.975684 3.622734 0.685434

1 1.174669 3.623375 0.709304

6 -2.134727 2.904933 0.350287

1 -2.962209 1.108697 -0.498262

1 -1.064971 4.617673 1.127997

1 -3.118000 3.353845 0.532590

6 1.586359 -0.481784 1.435452

1 0.578353 -0.048053 1.629527

1 2.181627 -0.341734 2.372730

7 -0.645413 -0.197086 -1.038478

1 0.173475 -0.392758 -1.615488

6 -1.542077 -1.274645 -0.910339

8 -1.274270 -2.342252 -1.404297

8 -2.608258 -0.969904 -0.153885

6 -3.551750 -2.065662 0.017023

1 -3.898615 -2.380872 -0.984213

1 -3.022360 -2.899234 0.511383

6 -4.638316 -1.445879 0.868715

1 -5.127511 -0.602455 0.362197

1 -5.419531 -2.184904 1.102637

1 -4.247524 -1.068986 1.823843

1 2.052686 2.554763 -1.732219

Molecule: B2b


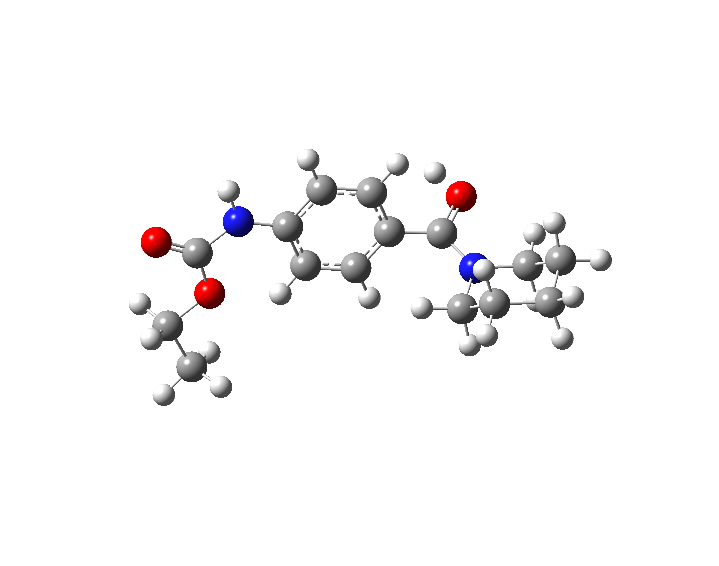


6 3.661779 1.027839 -1.791746

6 2.725450 1.285636 -0.601988

6 4.460468 0.421176 0.970023

6 5.409391 0.153322 -0.206528

6 5.128108 1.118648 -1.360758

1 2.848109 2.329288 -0.221410

1 1.659961 1.173658 -0.910815

1 3.454024 0.037279 -2.243329

1 3.448356 1.765526 -2.594444

1 4.632133 1.440221 1.395291

1 4.644946 -0.303614 1.798435

1 6.460914 0.256524 0.136891

1 5.317121 -0.896880 -0.549082

1 5.385149 2.156998 -1.071244

1 5.791209 0.885632 -2.220690

7 3.057138 0.353718 0.500710

6 2.213724 -0.571472 0.913978

6 0.828204 -0.740182 0.515986

6 -0.135432 0.232642 0.820440

6 0.452138 -1.911388 -0.160400

6 -1.453494 0.052653 0.449720

1 0.152736 1.135265 1.366528

6 -0.859297 -2.099637 -0.541758

1 1.203278 -2.672131 -0.393402

6 -1.840241 -1.113766 -0.247008

1 -2.186687 0.822957 0.711284

1 -1.144525 -3.009410 -1.073748

8 2.739510 -1.418367 1.812774

7 -3.130743 -1.373313 -0.666008

1 -3.318463 -2.270728 -1.137099

6 -4.282605 -0.564239 -0.541480

8 -5.343100 -0.965759 -0.948921

8 -4.042245 0.622227 0.039238

6 -5.219582 1.466030 0.181976

1 -5.622723 1.663059 -0.828131

1 -5.964987 0.916824 0.786106

6 -4.675015 2.700349 0.868705

1 -4.238811 2.465584 1.848876

1 -3.896030 3.192013 0.271234

1 -5.475191 3.437497 1.034371

1 2.049835 -2.038872 2.211778

Molecule: B3a


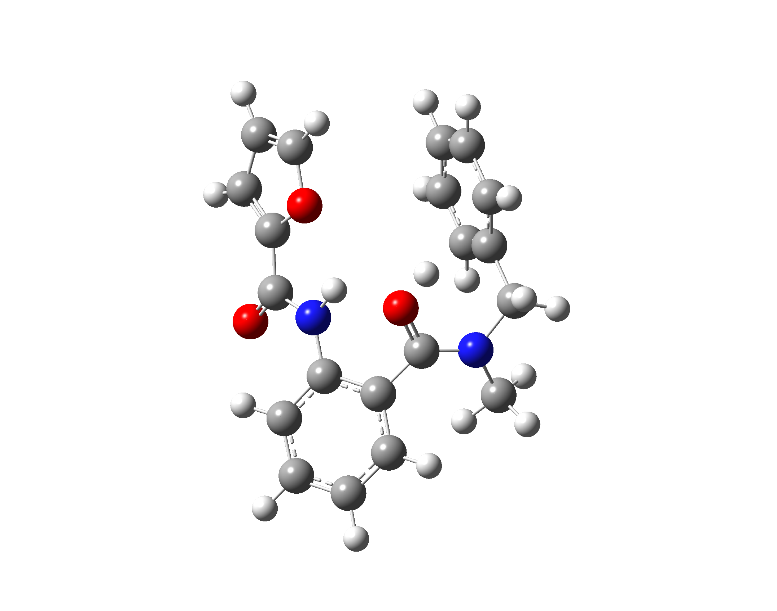


6 2.527640 1.252592 1.490368

6 1.291023 1.861290 1.291535

6 1.082244 2.661359 0.166666

6 2.118610 2.874797 -0.744222

6 3.356969 2.271223 -0.536379

6 3.560399 1.455539 0.575883

1 2.690220 0.622719 2.368079

1 0.497120 1.711978 2.022398

1 1.980082 3.529582 -1.605551

1 4.172698 2.447189 -1.241807

1 4.532810 0.985200 0.739919

6 -0.250868 3.322356 -0.063519

1 -0.329089 3.783847 -1.080091

1 -0.371638 4.179239 0.651629

7 -1.363814 2.363562 0.122558

6 -2.308742 2.670850 1.204072

1 -3.040436 3.435354 0.873336

1 -2.869800 1.774116 1.539138

1 -1.772942 3.060732 2.093605

6 -1.444918 1.238583 -0.579125

8 -0.537321 0.946078 -1.507554

6 -2.415378 0.173989 -0.414217

6 -1.955457 -1.153773 -0.227457

6 -3.798075 0.442299 -0.433565

6 -2.893987 -2.168066 0.058720

6 -4.702193 -0.568713 -0.189886

1 -4.147939 1.451341 -0.658703

6 -4.242995 -1.873091 0.075417

1 -2.549593 -3.192194 0.248593

1 -5.775422 -0.370535 -0.207405

1 -4.972101 -2.665082 0.282614

7 -0.588576 -1.406784 -0.330963

1 -0.029117 -0.778001 -0.928251

6 0.084267 -2.461143 0.329371

8 -0.496373 -3.221280 1.062183

6 1.514472 -2.483395 0.058666

6 2.548708 -3.267451 0.498218

8 1.978641 -1.520245 -0.836299

6 3.732071 -2.776050 -0.156925

1 2.503012 -4.092464 1.189048

6 3.333900 -1.732602 -0.949649

1 4.719440 -3.178927 -0.033846

1 3.808796 -1.060329 -1.630360

1 0.234897 1.578816 -1.633988

Molecule: B3b


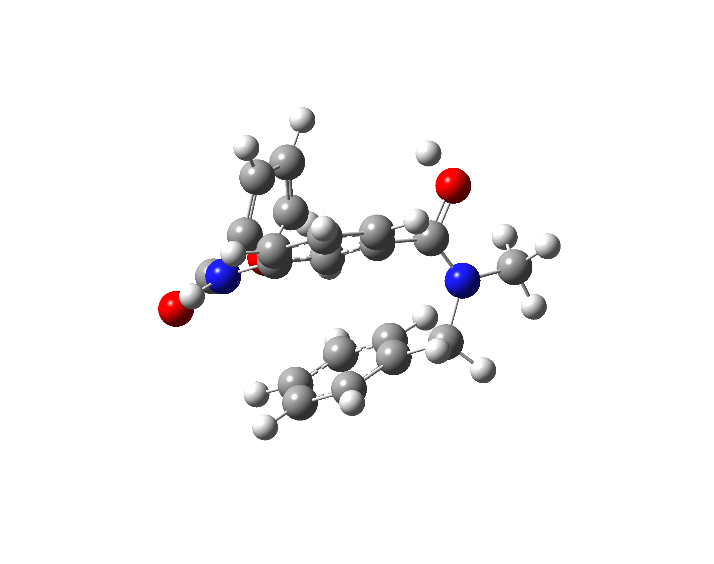


6 0.981366 1.258161 2.593691

6 -0.372409 0.977332 2.422856

6 -1.074073 1.559713 1.365801

6 -0.430922 2.446285 0.500678

6 0.925510 2.713538 0.668143

6 1.634765 2.113095 1.707933

1 1.533133 0.806806 3.421615

1 -0.873985 0.304808 3.118457

1 -0.980055 2.934521 -0.300947

1 1.435470 3.397561 -0.011681

1 2.704428 2.311016 1.827477

6 -2.529079 1.227137 1.188571

1 -3.162268 2.090060 1.522914

1 -2.829582 0.360240 1.832148

7 -2.856719 0.906159 -0.222122

6 -3.616402 1.890777 -1.002535

1 -3.073576 2.175747 -1.928607

1 -4.599894 1.474950 -1.308823

1 -3.798707 2.814613 -0.421904

6 -2.452266 -0.212946 -0.791105

8 -2.849588 -0.334068 -2.065315

6 -1.650557 -1.281568 -0.191287

6 -0.293066 -1.048997 0.013306

6 -2.241050 -2.500277 0.130983

6 0.506356 -2.063895 0.559638

1 0.135084 -0.071739 -0.224933

6 -1.443357 -3.504851 0.685347

1 -3.303877 -2.673998 -0.032392

6 -0.088992 -3.298450 0.901987

1 -1.895845 -4.466559 0.949139

1 0.523560 -4.095685 1.331796

7 1.865751 -1.878651 0.814697

1 2.288979 -2.428944 1.569512

6 2.681478 -0.842408 0.298391

8 3.648865 -0.541605 0.953087

6 2.340512 -0.282804 -1.006069

6 2.001657 -0.810188 -2.226689

8 2.449592 1.098341 -1.084252

6 1.886899 0.303578 -3.126312

1 1.865499 -1.841935 -2.488193

6 2.168535 1.425091 -2.389436

1 1.650509 0.236355 -4.169632

1 2.232766 2.473463 -2.587577

1 -2.515245 -1.185111 -2.497895

Molecule: B4a


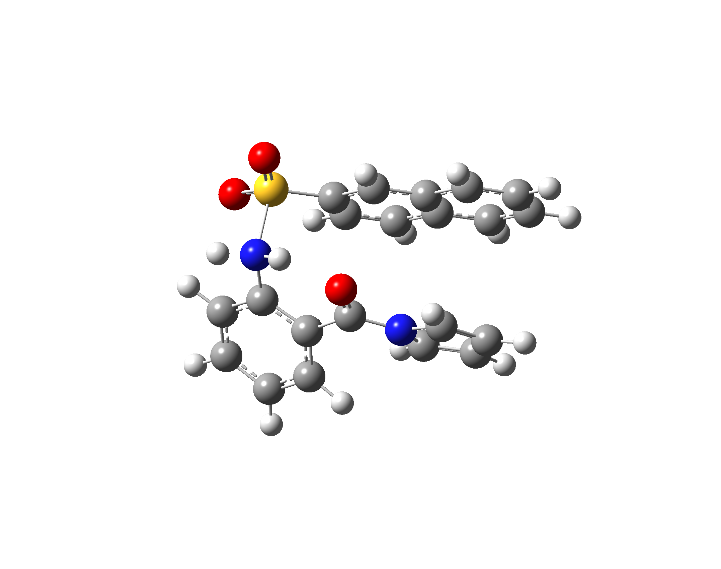


6 4.446313 -0.797639 0.960708

6 3.357081 -0.664008 1.784926

6 2.064454 -1.023855 1.323364

6 1.913768 -1.527631 0.008500

6 3.060381 -1.659322 -0.825024

6 4.296930 -1.299209 -0.357758

1 1.042169 -0.519352 3.169293

1 5.446606 -0.522033 1.309311

1 3.469037 -0.284173 2.801637

6 0.912489 -0.891939 2.150508

6 0.625733 -1.884382 -0.456089

1 2.935185 -2.049485 -1.837167

1 5.184401 -1.395418 -0.989124

6 -0.479399 -1.729671 0.358835

6 -0.326697 -1.235950 1.684074

1 0.541820 -2.279847 -1.480632

1 -1.198927 -1.142215 2.342761

16 -2.031174 -2.140353 -0.200257

8 -3.054428 -2.085403 0.787633

8 -2.031875 -2.998797 -1.333942

7 -2.535946 -0.536265 -1.187489

1 -1.882124 -0.388144 -2.023858

6 -2.564265 0.667359 -0.345008

6 -3.677342 0.876983 0.465685

6 -1.499214 1.585042 -0.361906

6 -3.762186 2.047661 1.221324

1 -4.480954 0.133638 0.526537

6 -1.630366 2.775964 0.350945

6 -2.755560 3.005703 1.143341

1 -4.632786 2.215177 1.863976

1 -0.857452 3.550787 0.280117

1 -2.843801 3.943168 1.701937

6 -0.297938 1.293766 -1.180872

8 -0.380069 0.565730 -2.161301

7 0.946395 1.804708 -0.843412

6 2.056962 1.797227 -1.730844

6 1.389798 2.315458 0.404605

6 3.138472 2.314510 -1.061873

1 1.945949 1.440268 -2.741591

6 2.720593 2.635282 0.282294

1 0.740252 2.370055 1.254501

1 4.128636 2.471109 -1.447267

1 3.360513 3.053973 1.036766

1 -3.481951 -0.691298 -1.604308

Molecule: B4b


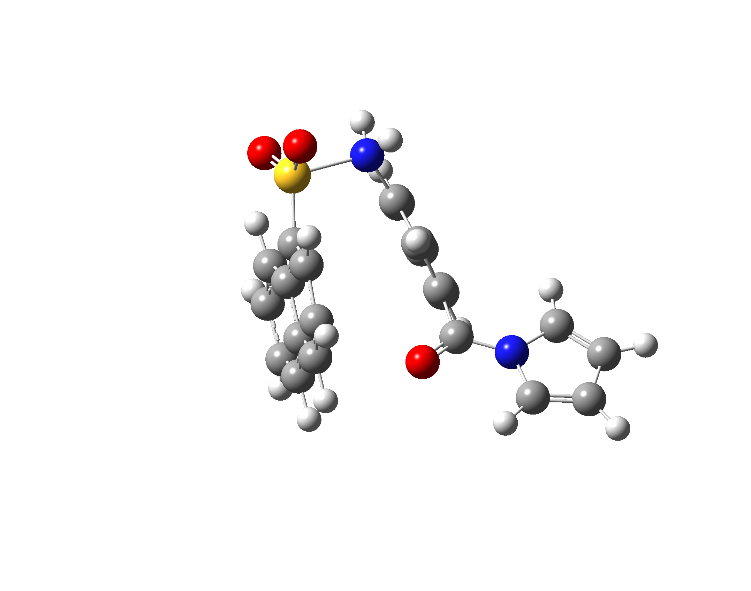


6 -1.098612 3.201289 1.725111

6 -0.510198 3.148097 0.489377

6 -0.895632 2.153977 -0.452742

6 -1.870436 1.182649 -0.106244

6 -2.491062 1.293620 1.166583

6 -2.114254 2.273131 2.053515

1 0.469233 2.887621 -1.980757

1 -0.810203 3.959588 2.456002

1 0.262331 3.868033 0.205189

6 -0.298763 2.141545 -1.739455

6 -2.157240 0.149223 -1.054320

1 -3.308818 0.628386 1.457008

1 -2.606919 2.353872 3.026936

6 -1.602812 0.209697 -2.331910

6 -0.682999 1.205069 -2.677539

1 -1.857573 -0.531925 -3.107776

1 -0.249236 1.219784 -3.680799

16 -3.079150 -1.216463 -0.678891

8 -3.979714 -1.135351 0.418853

8 -3.227229 -2.109013 -1.780996

7 -1.803885 -2.423104 0.256788

1 -2.347136 -2.926243 0.994091

6 -0.664372 -1.720733 0.853391

6 -0.654216 -1.456499 2.225268

6 0.354857 -1.262436 0.018559

6 0.385375 -0.702140 2.764111

1 -1.451382 -1.822028 2.877950

6 1.360636 -0.475360 0.574151

1 0.369135 -1.481590 -1.052218

6 1.390234 -0.196604 1.938718

1 0.409054 -0.494535 3.838603

1 2.190459 0.421753 2.359652

6 2.333815 0.161687 -0.358327

8 1.970656 0.978953 -1.176311

7 3.668944 -0.207320 -0.268809

6 4.242525 -1.188040 0.580713

6 4.703555 0.341358 -1.070736

6 5.590427 -1.237892 0.315375

1 3.645640 -1.746000 1.271944

6 5.880204 -0.276134 -0.721960

1 4.484845 1.107668 -1.793695

1 6.321880 -1.870380 0.781852

1 6.856234 -0.095573 -1.130520

1 -1.482453 -3.151797 -0.419277

Molecule: C1a


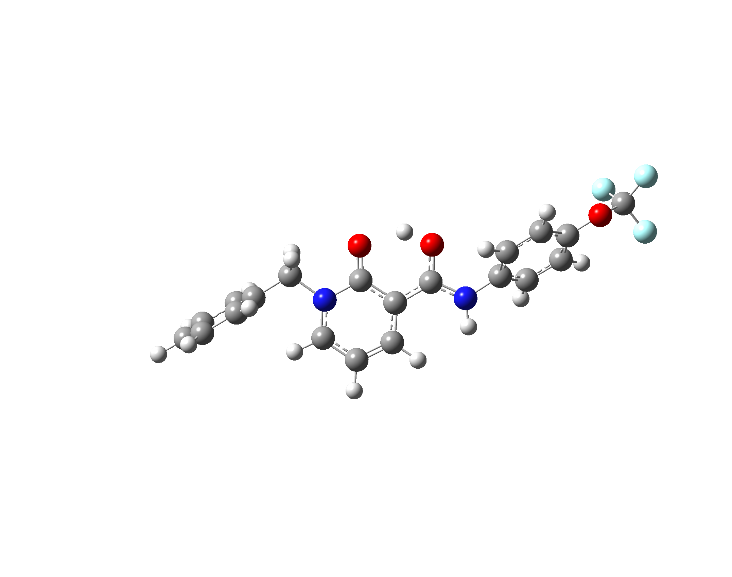


6 8.682645 -0.019070 -0.336035

6 7.927801 -0.124222 -1.503817

6 6.591359 -0.510893 -1.438003

6 6.009494 -0.788807 -0.199371

6 6.766512 -0.691681 0.970028

6 8.102619 -0.304686 0.899513

1 9.732202 0.278703 -0.390016

1 8.386639 0.087292 -2.471319

1 6.010058 -0.607077 -2.354864

1 6.322315 -0.929313 1.936433

1 8.698183 -0.234279 1.811468

6 4.580215 -1.225728 -0.128417

1 4.280961 -1.803294 -1.044003

1 4.406788 -1.929926 0.728851

7 3.643098 -0.078031 0.020195

6 2.258158 -0.412080 0.084632

6 4.051864 1.203177 0.093557

6 1.307469 0.660703 0.221211

8 1.972960 -1.603716 0.018734

6 3.127965 2.245873 0.233971

1 5.139332 1.415913 0.040546

6 1.770632 1.978066 0.296703

6 -0.083101 0.281878 0.271224

1 3.497405 3.273574 0.293043

1 1.069305 2.812469 0.407808

8 -0.475449 -0.975631 0.228093

7 -1.100896 1.150506 0.372769

6 -2.472632 0.717269 0.400191

1 -0.940889 2.156439 0.393561

6 -3.360815 1.298877 -0.509525

6 -2.895110 -0.239151 1.332920

6 -4.701386 0.925527 -0.492220

1 -3.016573 2.037817 -1.235499

6 -4.227512 -0.621286 1.350137

1 -2.189541 -0.685110 2.037744

6 -5.112139 -0.031908 0.433902

1 -5.391141 1.393431 -1.195895

1 -4.593213 -1.367977 2.062030

8 -6.399186 -0.455505 0.626118

6 -7.316261 -0.410488 -0.417814

9 -8.319083 -1.199681 -0.079341

9 -6.816152 -0.837519 -1.566086

9 -7.794756 0.808511 -0.604654

1 0.329421 -1.657184 0.119799

Molecule: C1b


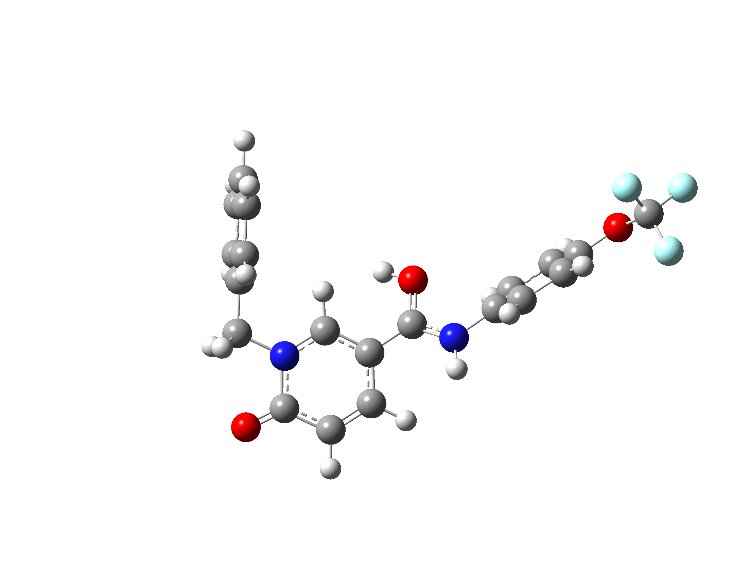


6 4.218638 -4.027982 -0.110397

6 4.219761 -3.453953 1.160104

6 4.569325 -2.114963 1.320596

6 4.915222 -1.349573 0.205667

6 4.923862 -1.926151 -1.066235

6 4.573033 -3.264683 -1.222431

1 3.953441 -5.080238 -0.233226

1 3.959298 -4.057042 2.031728

1 4.587200 -1.676317 2.318503

1 5.223503 -1.339396 -1.934609

1 4.591485 -3.721080 -2.213486

6 5.303390 0.086957 0.374185

1 5.742216 0.284533 1.388049

1 6.113275 0.373173 -0.351479

6 3.367537 3.340706 -0.010738

6 2.909252 0.582450 0.024171

8 5.646386 2.714474 0.368693

6 2.105489 2.883529 -0.170772

1 3.619301 4.406935 -0.027086

6 1.839344 1.476103 -0.153205

6 0.511400 0.972112 -0.305803

8 0.254696 -0.155701 -0.979704

7 -0.582649 1.537901 0.205585

6 -1.904752 1.019722 -0.014670

1 -0.502676 2.349917 0.826849

6 -2.722620 0.812006 1.099282

6 -2.357629 0.761397 -1.316481

6 -4.019308 0.337623 0.921568

1 -2.356939 1.011299 2.109501

6 -3.645493 0.288819 -1.501050

1 -1.709377 0.931268 -2.178518

6 -4.461943 0.080156 -0.374717

1 -4.643933 0.181731 1.802479

1 -4.033058 0.077946 -2.503312

8 -5.689681 -0.386928 -0.742498

6 -6.667759 -0.679253 0.203299

9 -6.289291 -1.626229 1.045716

9 -7.718740 -1.113054 -0.467014

9 -7.027586 0.382953 0.904164

1 2.716338 -0.508920 0.058496

1 1.276471 3.577956 -0.337175

6 4.490156 2.449776 0.192036

7 4.160774 1.015442 0.179762

1 1.044491 -0.501757 -1.498313

Molecule: C2a


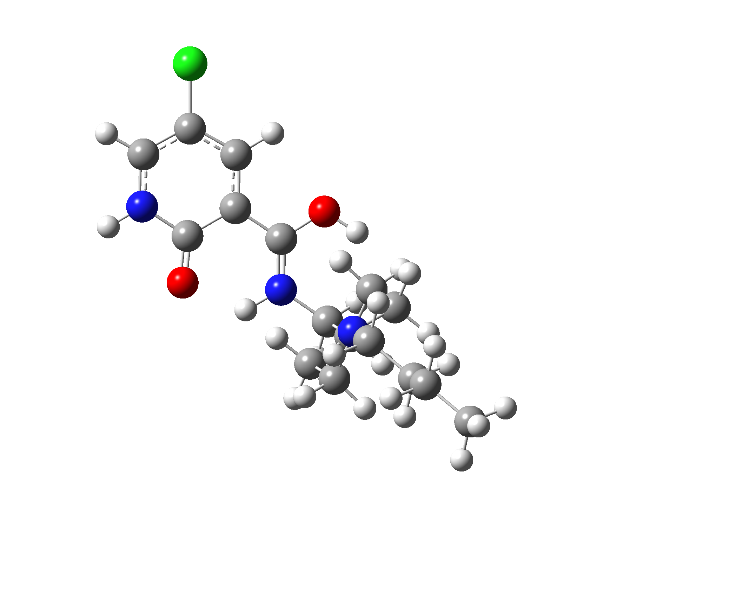


6 4.906107 0.839990 -1.355272

6 4.667877 1.193436 -0.033224

6 3.647055 0.603304 0.717062

6 2.852972 -0.365561 0.118368

1 5.713113 1.304480 -1.955771

1 3.480482 0.906340 1.764460

7 4.134552 -0.103805 -1.952570

1 4.282670 -0.389835 -2.929211

6 3.080003 -0.753492 -1.255986

8 2.442570 -1.591218 -1.869854

17 5.647969 2.373057 0.675341

6 1.765130 -1.023431 0.804508

8 1.580199 -0.605709 2.053180

7 1.031545 -1.990687 0.268191

1 1.192451 -2.256297 -0.743858

6 -0.118232 -2.621328 0.933738

1 -0.222926 -3.654369 0.507663

1 0.078337 -2.769290 2.019812

6 -1.433345 -1.840741 0.711706

6 -1.418876 -0.474753 1.410102

6 -1.723754 -1.650357 -0.783738

1 -2.258367 -2.459797 1.157165

6 -2.773256 0.233680 1.176078

1 -0.626450 0.175862 0.990849

1 -1.244691 -0.573160 2.495193

6 -3.055604 -0.892204 -0.964200

1 -0.920234 -1.066180 -1.271797

1 -1.775978 -2.621171 -1.308309

1 -2.758672 1.236813 1.657951

1 -3.594365 -0.343977 1.652547

1 -3.233832 -0.706802 -2.047806

1 -3.901688 -1.512496 -0.597665

7 -2.952437 0.416482 -0.280591

6 -4.082628 1.312226 -0.601854

1 -3.859651 2.303426 -0.144510

1 -4.068387 1.478400 -1.703860

6 -5.474346 0.827256 -0.158821

1 -5.498272 0.666721 0.933917

1 -5.705688 -0.152173 -0.615241

6 -6.542727 1.852898 -0.558099

1 -6.316818 2.836053 -0.101796

1 -6.524824 2.014988 -1.653345

6 -7.930657 1.384464 -0.124264

1 -7.991832 1.251217 0.961694

1 -8.700759 2.113385 -0.406990

1 -8.199581 0.429828 -0.590531

1 0.768416 -0.984780 2.520187

Molecule: C2b


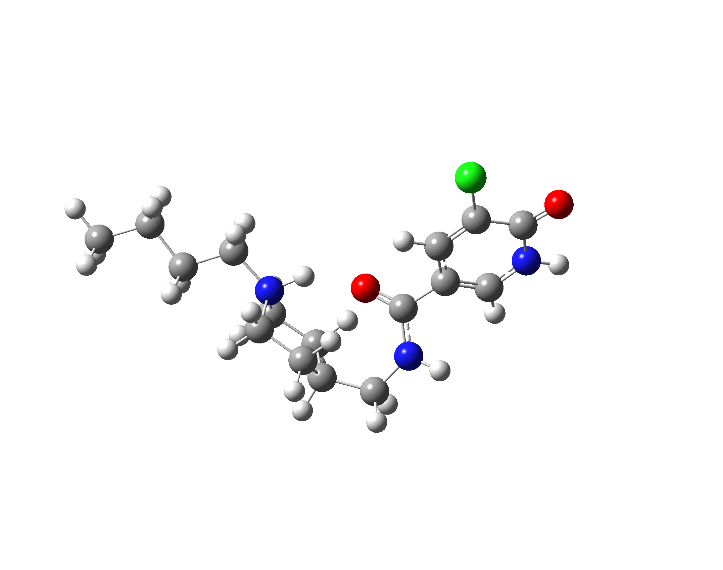


6 3.891539 1.310359 0.202649

6 2.666403 0.748716 0.340322

6 2.441215 -0.567617 -0.166760

1 1.834285 1.270812 0.823871

7 4.680723 -0.668829 -0.968332

1 5.433170 -1.153422 -1.463511

6 3.452580 -1.234660 -0.827127

17 4.217907 2.858203 0.789190

6 1.079067 -1.112305 -0.024954

8 0.100316 -0.410159 -0.242898

7 0.986719 -2.418339 0.387907

1 1.841764 -2.895563 0.671463

6 -0.211931 -3.262488 0.537956

1 -0.169818 -4.044685 -0.263512

1 -0.112872 -3.798748 1.517127

6 -1.608032 -2.598653 0.495795

6 -1.716343 -1.526805 1.595588

6 -1.921402 -2.154632 -0.945831

1 -2.351697 -3.403981 0.753402

6 -2.789224 -0.472618 1.325673

1 -0.736376 -1.026017 1.753951

1 -1.927191 -2.021456 2.569168

6 -2.982200 -1.059428 -1.037992

1 -0.993470 -1.816832 -1.460413

1 -2.254518 -3.035442 -1.536737

1 -2.700658 0.369459 2.050040

1 -3.811408 -0.895355 1.443992

1 -3.035751 -0.654370 -2.074881

1 -3.993292 -1.450350 -0.788849

7 -2.601751 0.050493 -0.082000

6 -3.384821 1.318649 -0.332893

1 -3.003249 2.104262 0.363504

1 -3.144542 1.674380 -1.364514

6 -4.896460 1.154357 -0.168630

1 -5.157940 0.813685 0.852020

1 -5.297525 0.388060 -0.860205

6 -5.591044 2.498074 -0.446131

1 -5.213290 3.278889 0.243460

1 -5.353394 2.852763 -1.468769

6 -7.102598 2.358743 -0.287764

1 -7.383546 2.051527 0.726532

1 -7.610208 3.314313 -0.483979

1 -7.523628 1.625518 -0.985814

1 -1.550488 0.254367 -0.220273

1 3.303189 -2.231105 -1.269347

6 4.996854 0.643179 -0.458035

8 6.125506 1.015077 -0.646876

Molecule: C3a


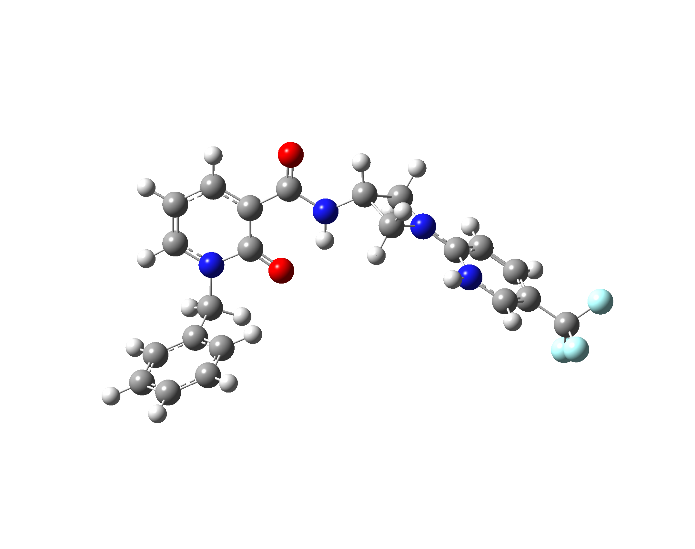


6 -6.128104 -2.653738 -0.656045

6 -4.858476 -2.082761 -0.577466

6 -4.007935 -2.399466 0.483006

6 -4.434504 -3.279630 1.473758

6 -5.706249 -3.847306 1.401282

6 -6.549661 -3.537375 0.335725

1 -6.789771 -2.421455 -1.488540

1 -3.012738 -1.956526 0.531335

1 -3.773881 -3.525962 2.303637

1 -6.039381 -4.536705 2.177586

1 -7.540660 -3.987864 0.276680

6 -4.396338 -1.112735 -1.629121

1 -5.044578 -1.127364 -2.535726

1 -3.367157 -1.385689 -1.987124

6 -5.540928 0.927673 -0.868777

6 -5.555079 2.177908 -0.282943

1 -6.467366 0.430490 -1.196693

6 -3.157547 2.092906 -0.030626

6 -4.350284 2.764623 0.142030

1 -6.497028 2.707970 -0.144555

1 -4.360037 3.758711 0.614534

7 -4.369285 0.256301 -1.068731

6 -3.126692 0.788526 -0.637907

8 -2.126657 0.103620 -0.818976

6 -1.916095 2.728442 0.428784

8 -1.870748 3.817109 0.965216

7 -0.725885 2.020391 0.223328

1 -0.742514 1.091925 -0.228529

6 0.513655 2.578763 0.640001

6 1.393980 1.597974 1.534549

6 1.659916 2.493292 -0.454306

1 0.394698 3.596868 1.086052

1 0.919161 0.624580 1.738155

1 1.760541 2.046819 2.471378

1 1.355290 2.036049 -1.410997

1 2.189371 3.441437 -0.645970

7 2.409066 1.578655 0.450962

6 3.488850 0.839531 0.227071

6 4.261731 0.922874 -0.980084

6 5.392577 0.172297 -1.128561

1 3.921188 1.600745 -1.767902

6 5.064444 -0.782543 1.072850

6 5.803723 -0.698372 -0.079332

1 5.989662 0.232543 -2.052944

1 5.321123 -1.436813 1.928454

7 3.916989 -0.031625 1.232309

6 7.054867 -1.508989 -0.278421

9 7.371482 -2.294344 0.747538

9 6.979890 -2.320200 -1.330365

9 8.129877 -0.752930 -0.485589

1 3.380784 -0.105742 2.099128

Molecule: C3b


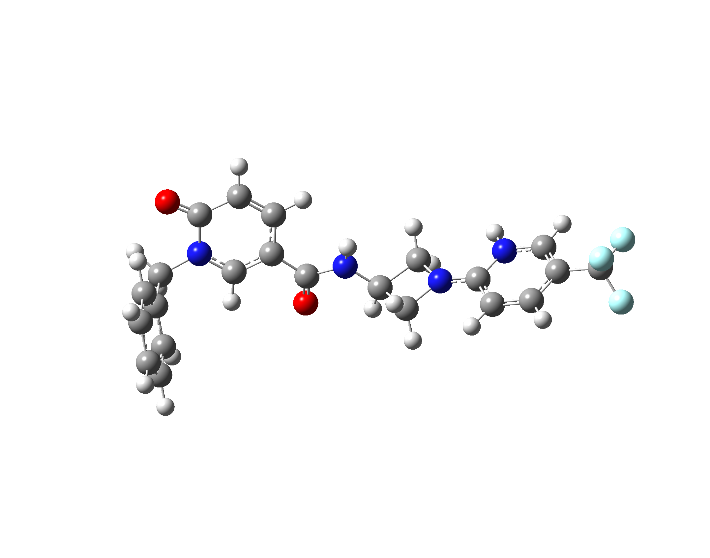


6 -6.898989 -1.126886 1.185974

6 -6.588663 -0.731137 -0.115668

6 -6.406294 -1.684862 -1.116933

6 -6.529840 -3.038539 -0.811249

6 -6.833731 -3.436818 0.489806

6 -7.019596 -2.481058 1.487661

1 -7.048580 -0.375027 1.964775

1 -6.178197 -1.380745 -2.136345

1 -6.394947 -3.786199 -1.591528

1 -6.933096 -4.496131 0.725512

1 -7.265232 -2.791810 2.503083

6 -6.441253 0.732186 -0.427720

1 -7.236987 1.330196 0.095024

1 -6.566902 0.947481 -1.513610

6 -3.685778 2.255111 1.710395

6 -2.744198 1.280503 -0.282950

6 -2.597926 1.979317 0.948015

1 -3.623613 2.787474 2.660250

1 -1.606106 2.306330 1.262771

7 -5.113289 1.204391 0.024674

6 -1.639837 0.940146 -1.168919

8 -1.752877 0.690372 -2.351111

7 -0.343916 0.878105 -0.606435

1 -0.200437 0.927173 0.387957

6 0.756387 0.537124 -1.445173

6 2.045146 1.445387 -1.237429

6 1.629817 -0.693269 -0.952747

1 1.941187 2.222931 -0.463470

1 2.435667 1.889425 -2.167084

1 1.297950 -1.161818 -0.011357

1 1.795360 -1.473811 -1.714094

7 2.779459 0.236857 -0.785148

6 4.042433 0.001748 -0.447202

6 4.527951 -1.299270 -0.085767

6 5.832868 -1.471701 0.279963

1 3.829296 -2.140320 -0.110994

6 6.251329 0.890526 -0.050319

6 6.712736 -0.352783 0.299548

1 6.210756 -2.469325 0.559097

1 6.876489 1.805021 -0.061546

7 4.934304 1.076174 -0.422858

6 8.142945 -0.580521 0.709380

9 8.895596 0.516010 0.710448

9 8.250516 -1.082160 1.936782

9 8.773518 -1.439745 -0.086665

6 -4.018751 0.922231 -0.714577

1 -4.160178 0.397370 -1.678345

6 -5.019083 1.857106 1.312450

8 -6.048307 2.011455 1.922994

1 4.609503 2.010033 -0.681413

1 0.448917 0.454264 -2.519699

Molecule: C4a


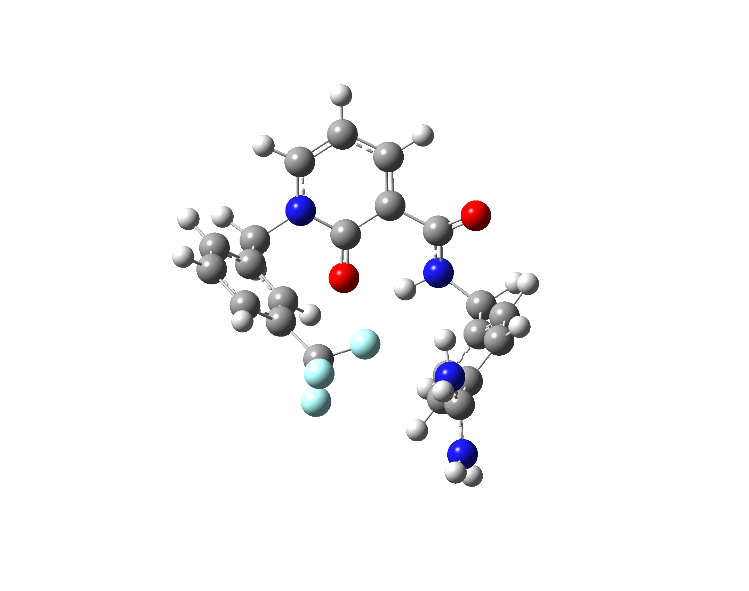


6 -2.399482 -1.250428 0.538305

6 -2.180324 -2.295114 -0.429496

6 -3.017935 -2.460174 -1.501939

6 -4.112105 -1.581473 -1.674699

6 -4.303288 -0.541523 -0.794882

7 -3.480008 -0.367291 0.288842

1 -2.834902 -3.262938 -2.230545

1 -4.791536 -1.726575 -2.513427

1 -5.121671 0.180392 -0.922699

8 -1.719208 -1.060794 1.538045

6 -0.968381 -3.131310 -0.294433

7 -0.437113 -3.261894 0.981754

1 -0.745213 -2.605817 1.718341

8 -0.460480 -3.694008 -1.247108

6 0.927381 -3.776645 1.104442

6 1.893055 -2.693611 0.679139

1 1.040401 -4.696878 0.475132

1 1.100864 -4.096726 2.158579

6 2.225087 -1.665704 1.565463

6 2.377047 -2.674777 -0.632653

6 3.017338 -0.607303 1.138341

1 1.839177 -1.678754 2.588240

6 3.173676 -1.623181 -1.065502

1 2.093438 -3.476893 -1.326959

6 3.484603 -0.582301 -0.181207

1 3.241772 0.208353 1.827885

1 3.551065 -1.619260 -2.089147

6 4.269508 0.567087 -0.648408

7 3.959231 1.176698 -1.811770

7 5.320778 1.013229 0.073949

1 3.133016 0.900162 -2.332343

1 5.860292 1.825996 -0.176716

6 -3.631840 0.829332 1.140998

6 -2.772341 1.944514 0.605263

1 -3.339633 0.579669 2.197111

1 -4.707188 1.118284 1.196173

6 -1.383426 1.845667 0.695328

6 -3.351995 3.060511 0.001173

6 -0.583900 2.838267 0.129832

1 -0.932847 0.986768 1.207877

6 -2.544671 4.067205 -0.525779

1 -4.437856 3.154606 -0.059238

6 -1.156204 3.957404 -0.473870

6 0.897484 2.646292 0.176089

1 -3.001226 4.945657 -0.986427

1 -0.540930 4.750838 -0.902809

9 1.296128 1.474472 -0.337941

9 1.431390 2.649528 1.397200

9 1.637193 3.528267 -0.498646

1 4.447921 1.985632 -2.160048

1 5.604215 0.533898 0.919575

Molecule: C4b


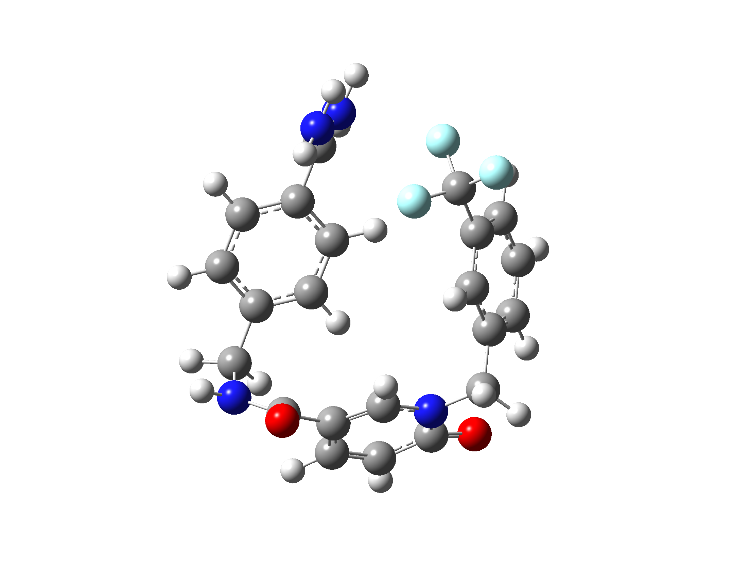


6 3.259869 2.108299 -0.687225

6 2.423686 0.642429 1.087066

6 4.157408 1.037658 -1.060015

8 3.120284 3.175199 -1.239073

6 3.204652 -0.394858 0.620133

1 1.774819 0.512165 1.968360

6 4.134883 -0.165427 -0.432279

1 4.854097 1.272748 -1.866262

6 3.097559 -1.688561 1.309843

8 3.010048 -1.796360 2.515091

7 3.110827 -2.876883 0.562001

6 2.566912 -2.930808 -0.792564

1 3.006089 -3.728049 1.112705

6 1.066176 -2.735202 -0.781064

6 0.230279 -3.796116 -0.423256

6 0.519514 -1.486289 -1.089254

6 -1.145639 -3.610226 -0.362041

1 0.657212 -4.774501 -0.186781

6 -0.855419 -1.291732 -1.031185

1 1.174606 -0.655852 -1.368953

6 -1.685439 -2.356209 -0.665379

1 -1.793813 -4.445698 -0.087908

1 -1.271806 -0.306872 -1.255678

6 -3.143255 -2.157758 -0.595620

7 -3.820534 -1.773019 -1.696719

1 -3.337687 -1.631675 -2.575210

1 -4.809981 -1.572601 -1.696874

6 1.539848 2.921487 0.953507

1 2.006275 3.923809 0.750887

1 1.445262 2.866426 2.064516

7 2.436117 1.856455 0.467960

6 0.203535 2.805028 0.269150

6 -0.831981 2.100995 0.881915

6 0.007534 3.391378 -0.982947

6 -2.056165 1.975173 0.225156

1 -0.681170 1.668474 1.873614

6 -1.223304 3.271663 -1.624628

1 0.822203 3.951726 -1.460864

6 -2.262608 2.567304 -1.021226

1 -1.377068 3.743653 -2.597726

1 -3.229750 2.509595 -1.523328

6 -3.167828 1.234108 0.895409

9 -2.771442 0.183590 1.623461

9 -4.049777 0.674939 0.054165

9 -3.914348 1.953720 1.723388

1 3.063527 -2.166160 -1.438282

1 2.835274 -3.916778 -1.248840

1 4.834474 -0.964996 -0.700887

1 -4.795044 -2.245247 0.664998

1 -3.295367 -2.635942 1.399526

7 -3.804410 -2.406438 0.551723

Molecule: C5a


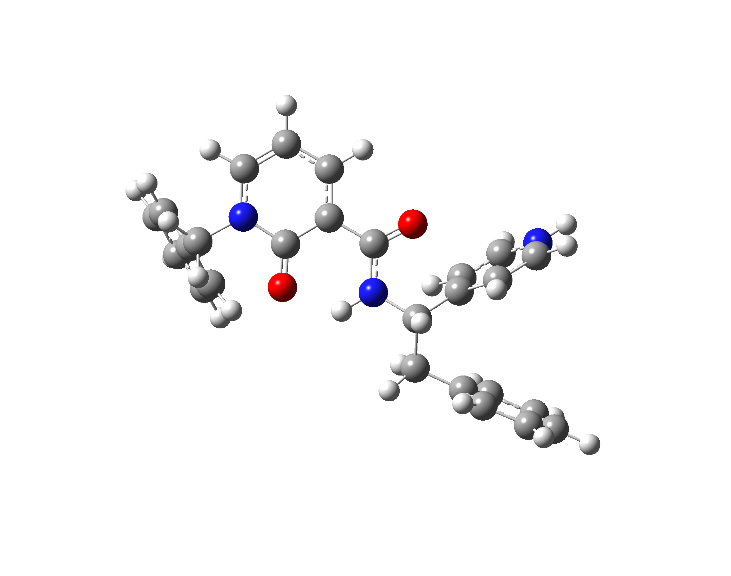


6 5.608666 2.206023 0.847004

6 6.502654 1.912761 -0.180876

6 6.022476 1.521638 -1.429876

6 4.651566 1.412313 -1.648817

6 3.756200 1.687377 -0.613179

6 4.236664 2.095297 0.632354

1 5.982633 2.536697 1.815806

1 7.576058 2.005101 -0.013245

1 6.721691 1.312738 -2.240579

1 4.281582 1.127845 -2.632879

1 3.544010 2.346247 1.432899

6 2.284085 1.526531 -0.833389

1 1.694289 1.999364 -0.019642

1 1.965660 2.062123 -1.756225

6 1.931032 0.024063 -0.952896

1 2.287454 -0.363311 -1.951930

6 2.609493 -0.750211 0.163818

6 3.629321 -1.668685 -0.133802

6 2.283853 -0.521133 1.514114

6 4.265965 -2.350235 0.890008

1 3.906127 -1.870587 -1.174701

6 2.937579 -1.221832 2.510371

1 1.483050 0.185052 1.766212

7 3.919718 -2.124599 2.194268

1 5.058054 -3.091431 0.695666

1 2.696407 -1.089878 3.576695

7 0.479845 -0.153534 -0.871159

1 -0.136614 0.567323 -1.288505

6 -0.012307 -1.377636 -0.469033

8 0.786566 -2.201099 -0.025555

6 -1.455178 -1.611932 -0.539991

6 -1.970152 -2.819210 -0.118487

6 -3.355666 -3.046774 -0.175820

1 -1.298861 -3.601154 0.262116

6 -4.194679 -2.055576 -0.647712

1 -3.765139 -4.000126 0.154450

1 -5.286310 -2.198504 -0.685444

6 -2.315446 -0.571254 -1.046227

8 -1.940356 0.523908 -1.438957

7 -3.705190 -0.863034 -1.093646

6 -4.648888 0.201657 -1.505635

1 -4.163159 0.860895 -2.271610

1 -5.522745 -0.256664 -2.025252

6 -5.065982 0.996379 -0.298685

6 -6.242220 0.673326 0.378673

6 -4.264698 2.048172 0.146079

6 -6.610986 1.400139 1.508258

1 -6.878233 -0.136483 0.027579

6 -4.637071 2.772519 1.276104

1 -3.348510 2.302527 -0.390159

6 -5.808199 2.447491 1.958559

1 -7.530765 1.152698 2.038019

1 -4.014072 3.596157 1.622275

1 -6.100028 3.016409 2.841629

1 4.388623 -2.644581 2.941181

Molecule: C5b


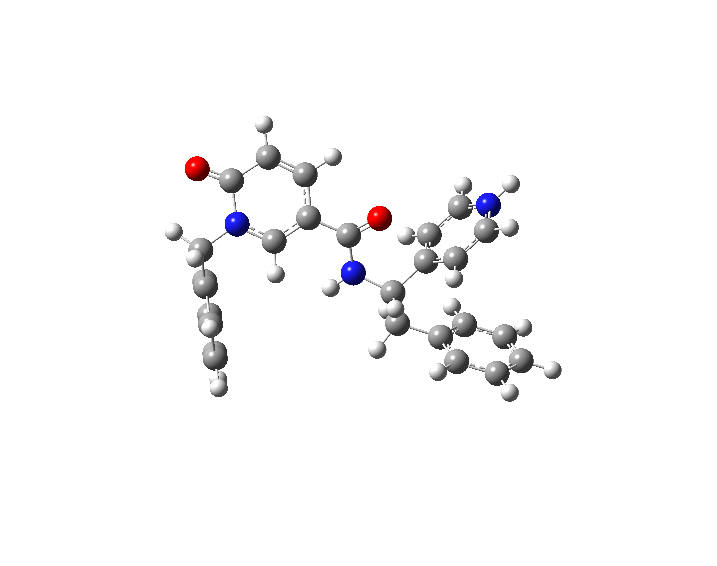


6 -5.679917 1.644349 -1.150579

6 -6.263423 1.984582 0.068211

6 -5.459658 2.221331 1.182432

6 -4.075234 2.108026 1.082258

6 -3.491856 1.748820 -0.134405

6 -4.295481 1.527264 -1.253876

1 -6.306123 1.483950 -2.028563

1 -7.347122 2.081554 0.146292

1 -5.915126 2.508013 2.131544

1 -3.451401 2.315958 1.950108

1 -3.845843 1.281897 -2.213685

6 -2.005683 1.586729 -0.233413

1 -1.675682 1.527919 -1.291637

1 -1.492384 2.485277 0.176656

6 -1.564626 0.325840 0.546382

1 -1.648163 0.526778 1.655271

6 -2.461932 -0.843399 0.177128

6 -3.317847 -1.401803 1.140840

6 -2.507258 -1.349115 -1.135156

6 -4.152097 -2.452967 0.798742

1 -3.307906 -1.030859 2.172170

6 -3.351159 -2.401344 -1.441155

1 -1.845277 -0.931916 -1.902423

7 -4.163038 -2.942342 -0.479066

1 -4.822105 -2.931522 1.532333

1 -3.398558 -2.841732 -2.450177

7 -0.164787 0.000737 0.248345

1 0.438956 0.746674 -0.052629

6 0.263211 -1.313628 0.423540

8 -0.603876 -2.148358 0.650047

6 1.680875 -1.602349 0.271231

6 2.052672 -2.912311 -0.157785

6 3.360641 -3.223948 -0.336020

1 1.257592 -3.645275 -0.329788

1 3.692652 -4.210474 -0.664158

6 2.669093 -0.674470 0.560965

7 3.976369 -0.965856 0.379873

6 5.002854 0.061297 0.664921

1 5.235415 0.046275 1.756777

1 5.962911 -0.231439 0.153914

6 4.551693 1.413953 0.192141

6 4.475346 1.677489 -1.176670

6 4.208590 2.401390 1.116035

6 4.040871 2.924003 -1.619991

1 4.764156 0.911846 -1.897634

6 3.777585 3.648646 0.668659

1 4.290644 2.209853 2.185367

6 3.689118 3.908920 -0.697811

1 3.988958 3.133831 -2.688359

1 3.521389 4.425561 1.388555

1 3.359379 4.887804 -1.045837

1 2.419202 0.323881 0.955909

6 4.414430 -2.264640 -0.096855

8 5.603103 -2.407572 -0.240892

1 -4.774021 -3.730182 -0.715918

Molecule: D1a


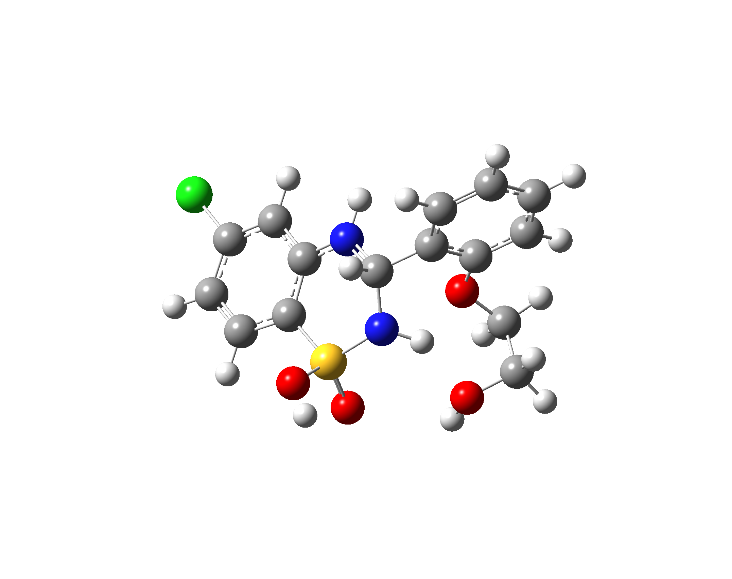


6 2.641508 0.909797 -0.453999

6 3.823643 1.560282 -0.804337

6 4.366617 2.483107 0.083777

6 3.740815 2.746156 1.305449

6 2.561326 2.093044 1.642398

6 1.991935 1.166530 0.765085

1 5.291958 3.007570 -0.176152

1 4.184005 3.465549 1.997190

1 2.079314 2.304053 2.600757

7 -0.410905 1.171975 0.455729

6 -1.630859 0.625300 0.197506

6 -1.902723 -0.757415 0.377495

16 -0.666634 -1.828814 0.671422

7 0.761353 -0.951064 0.779714

1 1.562111 -1.223295 0.123140

6 -2.667446 1.485174 -0.272352

1 -2.448750 2.535028 -0.472096

6 -3.233483 -1.225702 0.214816

1 -3.464444 -2.282335 0.406151

6 -3.925088 0.967875 -0.446100

6 -4.238910 -0.383609 -0.185619

1 -5.263337 -0.743633 -0.308214

17 -5.166185 1.984173 -0.989221

8 -0.928500 -2.343098 2.174622

8 -0.483149 -2.948386 -0.190483

1 4.310284 1.366062 -1.757809

8 1.981348 0.010692 -1.238662

6 2.715185 -0.734820 -2.206087

1 3.396057 -0.103479 -2.797600

1 1.890604 -1.086053 -2.864647

6 3.420363 -1.885879 -1.489829

1 4.171888 -1.527741 -0.756242

1 3.898994 -2.594991 -2.188068

8 2.481187 -2.556204 -0.672677

1 1.873552 -3.122227 -1.203450

1 -0.225212 2.142496 0.201947

6 0.715055 0.465688 1.143991

1 0.517532 0.541963 2.261300

1 -0.411125 -3.092457 2.544138

Molecule: D1b


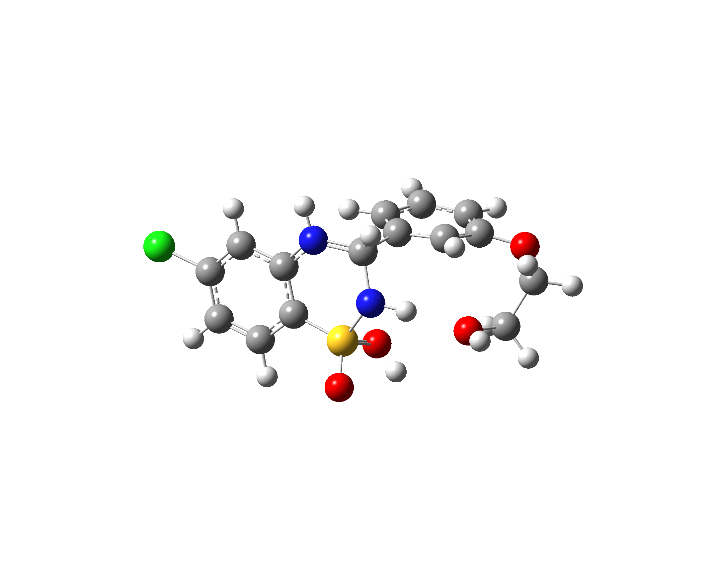


6 2.562567 0.989887 0.755101

6 3.377604 1.536230 -0.229157

6 2.883802 2.485605 -1.139611

6 1.531605 2.790606 -1.104231

6 0.665016 2.120303 -0.227498

6 1.185650 1.211811 0.680531

1 2.989722 0.388889 1.554070

1 3.558419 2.953703 -1.858410

1 1.125369 3.539173 -1.790144

1 -0.401931 2.320413 -0.283841

8 4.677579 1.178633 -0.403091

6 5.068487 -0.085123 0.132018

1 5.054500 -0.052777 1.235583

1 6.126178 -0.120023 -0.212298

6 4.236417 -1.237737 -0.436762

1 4.867632 -2.068306 -0.802894

1 3.569629 -0.900226 -1.255895

8 3.327403 -1.753221 0.528846

1 3.786541 -2.328387 1.174119

7 -1.059666 0.631589 1.616468

6 -2.013075 0.191343 0.738155

6 -1.810090 -0.925246 -0.109722

16 -0.369156 -1.760921 -0.111303

7 0.588799 -1.111917 1.119539

1 1.614113 -1.361978 1.106515

6 -3.272031 0.853465 0.738151

1 -3.436855 1.709781 1.394789

6 -2.869098 -1.353650 -0.951140

1 -2.727811 -2.223073 -1.609065

6 -4.261137 0.395957 -0.098301

6 -4.080618 -0.707376 -0.956107

1 -4.894121 -1.039680 -1.606959

17 -5.757449 1.185285 -0.110291

8 -0.395945 -3.181697 -0.102703

8 0.395044 -1.312677 -1.467368

1 -1.273595 1.433498 2.213227

6 0.369076 0.287912 1.566915

1 0.747004 0.331116 2.644707

1 0.896702 -1.967159 -1.995284

#

# Molecular Structures & XYZ Coordinates of Optimized CRGs

XYZ atomic coordinate data of the lowest energy isomer of each acrylamide CRG, as calculated at the B3LYP/6-311++G(d,p) level of theory, are provided below. Sites of protonation are highlighted on the chemical structure in red, and sites of deprotonation are highlighted in green.

### Acrylamide CRG 1a


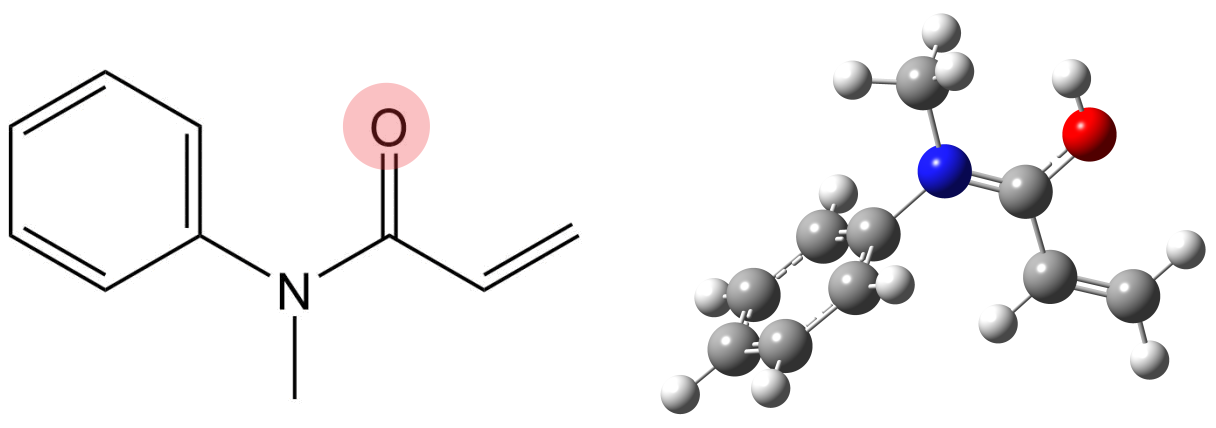


C -0.665350 0.329556 0.003354

C -1.327230 0.171927 -1.211722

C -2.645670 -0.279407 -1.203751

C -3.282874 -0.560136 0.004397

C -2.606682 -0.391779 1.211970

C -1.287781 0.058701 1.219233

H -0.824066 0.393354 -2.145971

H -3.171682 -0.410878 -2.141443

H -4.308343 -0.909336 0.004769

H -3.102598 -0.609562 2.149996

H -0.755074 0.194412 2.153467

N 0.699845 0.846953 0.004964

C 1.744626 0.043200 -0.005859

O 2.970688 0.526982 0.021289

C 1.632362 -1.405425 -0.052477

H 0.632334 -1.811907 -0.096255

C 2.712360 -2.196528 -0.043693

H 3.719988 -1.801547 0.001708

H 2.600417 -3.273190 -0.082272

C 0.868712 2.318052 0.037737

H 1.332586 2.633890 0.976782

H 1.457195 2.660020 -0.818606

H -0.117249 2.768879 -0.025675

H 3.017247 1.492380 0.063312

###

### Acrylamide CRG 1b


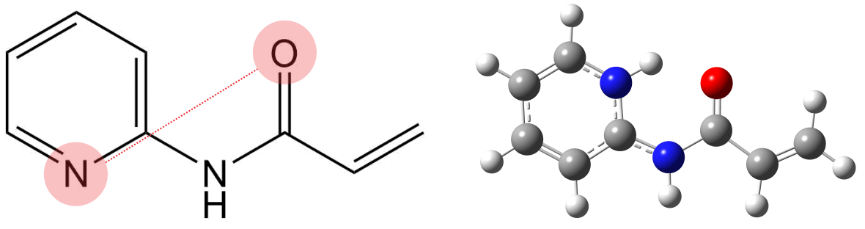


C -0.669624 0.443476 -0.000031

C -1.715973 1.374704 0.00013989

C -3.021417 0.919619 0.000103

C -3.301784 -0.456710 -0.000104

C -2.249292 -1.337044 -0.000176

N -0.975213 -0.874910 -0.000169

H -3.834398 1.635882 0.000233

H -1.490884 2.433538 0.000309

H -4.317609 -0.826489 -0.000182

H -2.360060 -2.412903 -0.000303

N 0.653512 0.816116 -0.000197

H 0.836128 1.810519 0.000265

C 1.761856 -0.054852 0.000153

C 3.068549 0.619231 -0.000075

H 3.089165 1.704251 -0.000088

C 4.197276 -0.094239 -0.000265

H 4.169935 -1.178035 0.000004

H 5.166376 0.389124 -0.000306

O 1.591346 -1.265540 0.000504

H -0.155066 -1.505114 0.000124

### Acrylamide CRG 1c (deprotonated)


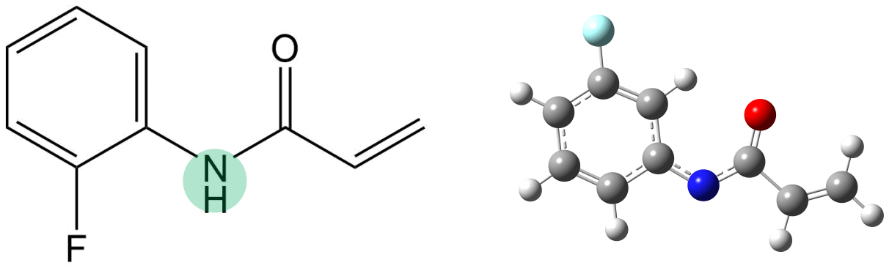


C 0.270095 0.616087 0.000570

C 0.892302 -0.665756 0.000248

C 2.270589 -0.746912 -0.000098

C 3.124756 0.345104 -0.000204

C 2.519447 1.609746 -0.000350

C 1.140701 1.742951 -0.000081

H 0.278269 -1.553979 0.000060

H 4.199147 0.209500 -0.000689

H 3.145236 2.497880 -0.000631

H 0.676812 2.722954 -0.000248

N -1.076319 0.887046 0.000675

C -2.004026 -0.088401 0.000401

O -1.863466 -1.332990 0.000350

C -3.397958 0.486986 -0.000081

H -3.455829 1.571840 0.000003

C -4.499793 -0.266650 -0.000768

H -4.409724 -1.348533 -0.000964

H -5.494836 0.168682 -0.001254

F 2.845134 -2.001409 -0.000181

### Acrylamide CRG 1d


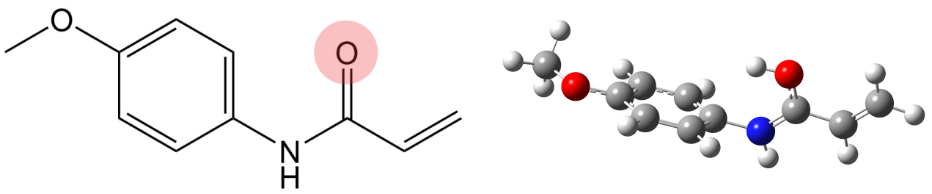


C 0.229787 0.209839 -0.498307

C -0.292863 -1.079313 -0.679524

C -1.637032 -1.307904 -0.449918

C -2.481525 -0.253296 -0.055264

C -1.951207 1.037141 0.108085

H -2.066642 -2.292198 -0.586326

H 0.344286 -1.888795 -1.018627

N 1.631241 0.457602 -0.755456

H 1.891979 0.975667 -1.588326

C 2.608864 0.054432 0.032365

C 3.996999 0.318465 -0.295699

H 4.178395 0.853987 -1.220870

C 5.003395 -0.072045 0.494295

H 4.830910 -0.610264 1.418777

H 6.030187 0.143042 0.224950

O 2.347178 -0.593185 1.136530

C -0.597395 1.263671 -0.113814

H -0.190055 2.260081 0.014660

H -2.580841 1.863644 0.405970

O -3.766497 -0.580402 0.134589

C -4.711818 0.428502 0.518116

H -5.665401 -0.086507 0.600107

H -4.445902 0.863984 1.484951

H -4.779919 1.207710 -0.245497

H 1.385647 -0.701827 1.267468

### Acrylamide CRG 1e (deprotonated)


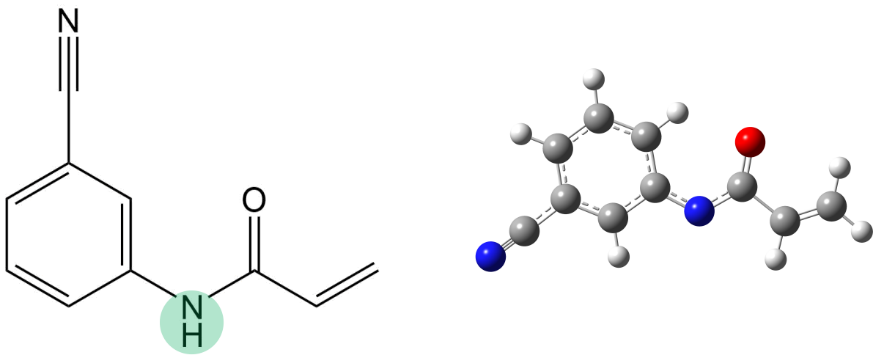


C -2.615838 1.224110 -0.00001588

C -2.463993 -0.176171 -0.000008

C -1.195624 -0.758309 0.000027

C -0.009298 0.021495 0.000072

C -0.189354 1.435984 0.000031

C -1.462223 2.002214 0.000015

H -3.603676 1.668275 -0.000048

H -1.087601 -1.836242 0.000022

H 0.695548 2.055048 0.000015

H -1.554898 3.084938 -0.000004

C -3.628597 -1.008904 -0.000037

N -4.579570 -1.670522 -0.000049

N 1.169186 -0.679313 0.000069

C 2.367409 -0.062768 0.000009

C 3.494921 -1.063053 0.000034

H 3.194635 -2.106888 0.000169

C 4.782212 -0.711248 -0.000083

H 5.579219 -1.448749 -0.000043

H 5.052530 0.340111 -0.000206

O 2.639906 1.159031 -0.000039

### Acrylamide CRG 1f (deprotonated)


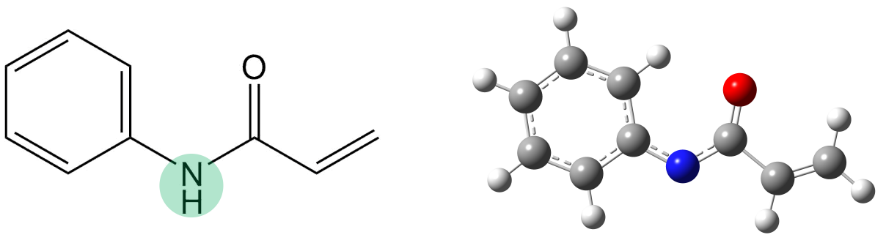


C -0.614869 -0.326877 -0.000095

C -1.613449 -1.338566 0.000055

C -2.969398 -1.043526 0.000128

C -3.409490 0.285478 0.000057

C -2.449159 1.299374 -0.000062

C -1.084447 1.017295 -0.000127

H -1.269929 -2.368096 0.000097

H -3.693751 -1.854632 0.000229

H -4.469419 0.520905 0.000108

H -2.769005 2.339198 -0.000101

H -0.350893 1.810041 -0.000185

N 0.692142 -0.763459 -0.000147

C 1.734056 0.085138 -0.000032

O 1.758062 1.339411 0.000140

C 3.043732 -0.663964 -0.000092

H 2.961893 -1.747443 -0.000295

C 4.234758 -0.060025 0.000112

H 4.286497 1.024464 0.000317

H 5.164712 -0.621485 0.00008390

### Acrylamide CRG 1g


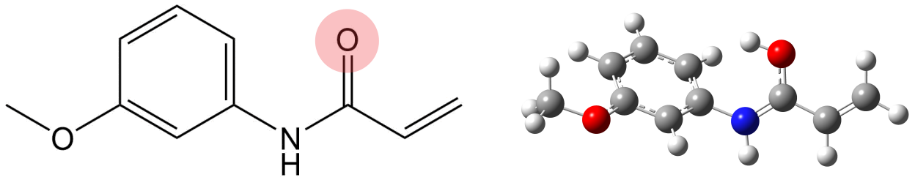


C 0.106077 0.124677 -0.484838

C -0.106476 1.507410 -0.454613

C -1.397829 1.959810 -0.189376

C -2.445996 1.069995 0.026271

C -2.214449 -0.313609 -0.022714

H -1.594265 3.024902 -0.169817

H 0.688732 2.204468 -0.690979

H -3.436745 1.456023 0.221720

N 1.433934 -0.384079 -0.746706

H 1.559729 -1.001546 -1.541630

C 2.491300 -0.150760 0.007016

C 3.783987 -0.718854 -0.325460

H 3.825171 -1.333166 -1.217885

C 4.870863 -0.502919 0.424160

H 4.839134 0.111598 1.315980

H 5.822512 -0.943791 0.153900

O 2.402947 0.591321 1.078555

C -0.916905 -0.787751 -0.274775

H -0.742560 -1.856622 -0.291940

O -3.146909 -1.260582 0.163700

C -4.504467 -0.876618 0.428639

H -5.051199 -1.809572 0.538218

H -4.572184 -0.300394 1.355031

H -4.917023 -0.305119 -0.406692

H 1.496234 0.927575 1.217131

### Acrylamide CRG 1h (deprotonated)


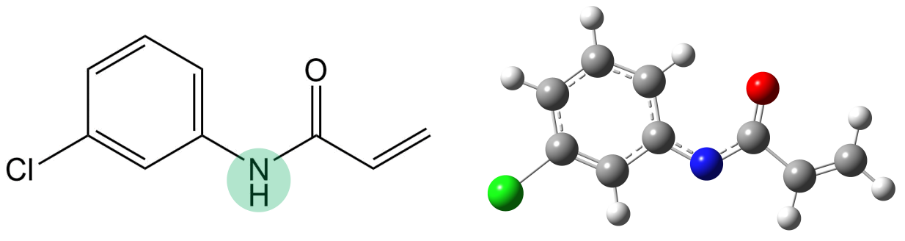


C -0.122258 0.058767 0.000360

C 0.048556 1.473207 -0.000032

C 1.319552 2.037828 -0.000150

C 2.482325 1.261893 0.000065

C 2.310045 -0.120376 0.000031

H 1.415113 3.120674 -0.000256

H -0.837460 2.089996 -0.000249

H 3.469866 1.704637 -0.000103

N -1.297472 -0.650556 -0.000002

C -2.500557 -0.045266 -0.000090

C -3.617148 -1.058951 -0.000335

H -3.304141 -2.099173 -0.001010

C -4.908997 -0.723072 0.000221

H -5.191381 0.325204 0.000785

H -5.697509 -1.469897 0.00000495

O -2.789476 1.173122 -0.000018

C 1.068931 -0.722710 0.000166

H 0.968087 -1.800313 0.000237

Cl 3.770167 -1.157066 -0.000039

### Acrylamide CRG 2a


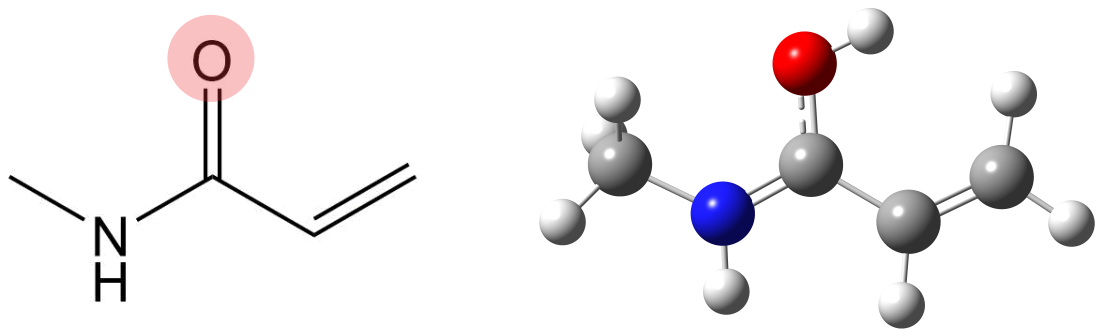


C -2.519458 -0.067968 0.084366

H -2.633751 0.997272 0.264703

H -3.441519 -0.635409 0.052274

C -1.341486 -0.678035 -0.072456

H -1.294507 -1.751223 -0.221506

C -0.057206 0.012559 -0.034909

O 0.066229 1.318514 -0.039392

N 1.062704 -0.661601 0.008037

H 0.987167 -1.670246 0.058751

C 2.411975 -0.071962 0.044080

H 3.115772 -0.821621 -0.310655

H 2.445066 0.802888 -0.602027

H 2.665594 0.217565 1.065485

H -0.775534 1.776308 -0.174632

### Acrylamide CRG 2b


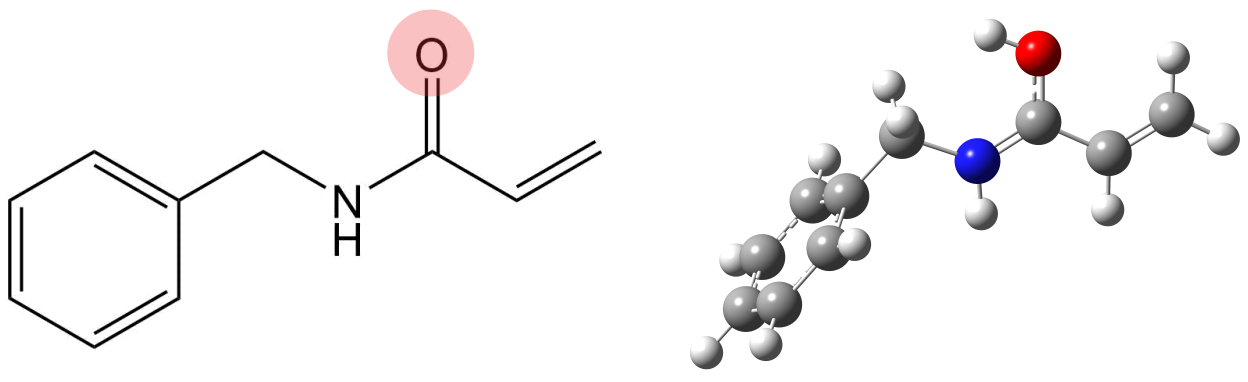


C -1.949027 -0.023707 -1.222624

C -3.226768 -0.572430 -1.146136

C -3.888247 -0.631399 0.079804

C -3.274905 -0.138011 1.230395

C -1.997104 0.411679 1.157557

C -1.327317 0.466954 -0.069339

H -1.444727 0.033168 -2.182159

H -3.708156 -0.943767 -2.042802

H -4.884935 -1.052545 0.136567

H -3.793451 -0.171647 2.180973

H -1.530433 0.808390 2.053712

C 0.067554 1.033415 -0.144904

H 0.245327 1.486479 -1.125476

H 0.221359 1.787561 0.633302

N 1.074443 -0.050542 0.053408

H 0.697869 -0.972551 0.247486

C 2.378402 0.072029 -0.002658

C 3.251541 -1.069988 0.203812

H 2.762170 -2.016417 0.403937

C 4.584219 -0.969970 0.151238

H 5.082587 -0.028858 -0.048359

H 5.207417 -1.841577 0.309270

O 2.948476 1.229262 -0.248279

H 2.325976 1.960025 -0.376943

### Acrylamide CRG 2c


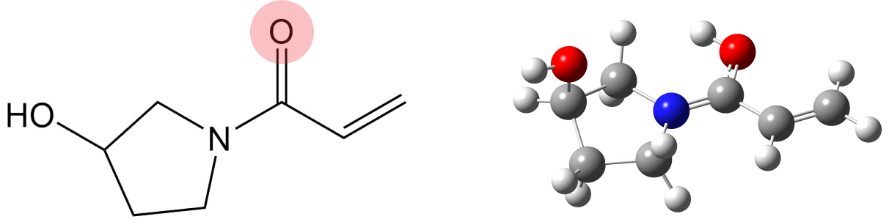


C -2.021792 -1.122346 -0.593118

C -0.578866 -1.357014 -0.136226

C -1.002660 1.043423 -0.475058

C -2.312110 0.320449 -0.153678

H -2.104365 -1.208932 -1.679944

H -2.710182 -1.838248 -0.143917

H -0.038229 -2.058279 -0.769838

H -0.532024 -1.682519 0.903817

H -0.870287 1.893533 0.197904

H -0.967376 1.367741 -1.519102

H -3.148960 0.769084 -0.695266

N 0.036249 0.005641 -0.235881

O -2.476569 0.430028 1.252385

H -3.357197 0.134842 1.509761

C 1.310313 0.267318 -0.084304

C 2.297065 -0.758734 0.214128

H 1.933359 -1.767996 0.345638

C 3.599739 -0.476624 0.336451

H 3.986879 0.527421 0.213330

H 4.310323 -1.260861 0.567089

O 1.776005 1.498031 -0.195033

H 1.098687 2.161421 -0.386294

### Acrylamide CRG 2d


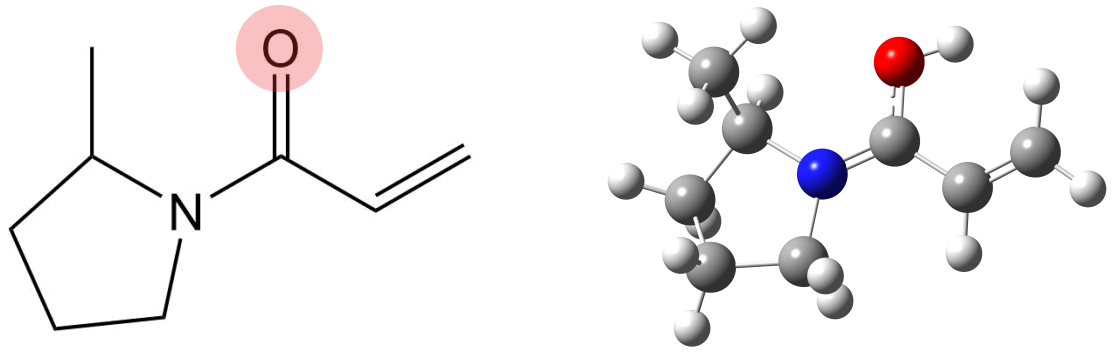


C -0.591979 -1.536016 0.186758

C -2.115414 -1.379584 0.262939

C -2.406070 -0.167094 -0.634635

C -1.249778 0.802519 -0.347670

H -0.142906 -1.900664 1.110286

H -0.298196 -2.189681 -0.638484

H -2.426655 -1.188248 1.291513

H -2.621351 -2.284758 -0.071493

H -2.398532 -0.460253 -1.687749

H -3.371468 0.292572 -0.423729

H -0.987382 1.406674 -1.216832

C -1.477578 1.709576 0.864936

H -0.599481 2.318394 1.082249

H -1.737443 1.135792 1.757167

H -2.305989 2.385495 0.643357

N -0.106663 -0.153029 -0.109984

C 1.146702 0.186356 -0.195443

C 2.239906 -0.772420 -0.031216

H 1.991910 -1.816636 -0.171056

C 3.484814 -0.413712 0.296623

H 3.781678 0.611261 0.499024

H 4.262754 -1.159460 0.404180

O 1.387193 1.465344 -0.445886

H 2.318528 1.620209 -0.655216

### Acrylamide CRG 3a


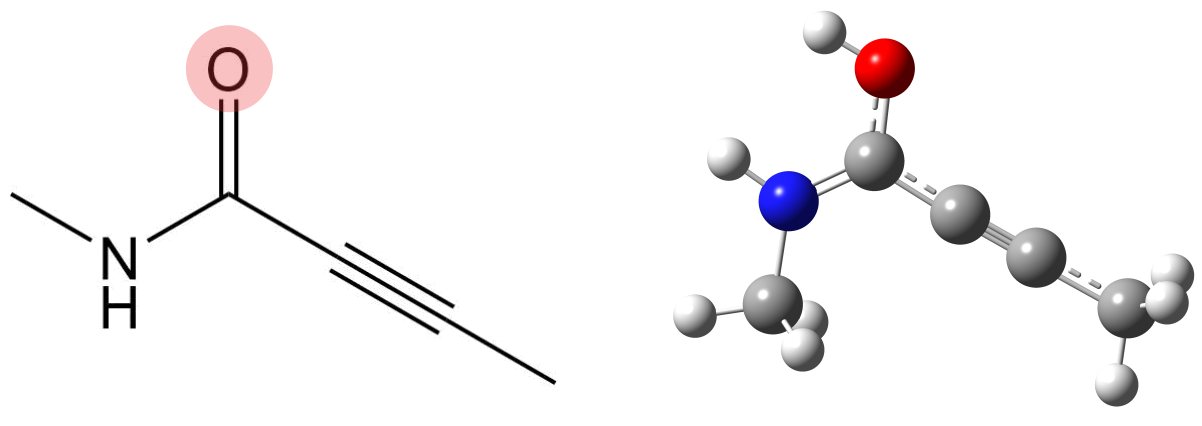


C 1.477765 1.798457 -0.000233

H 0.930602 2.080281 -0.899665

H 2.439918 2.305241 0.011964

H 0.909953 2.076879 0.887264

N 1.735942 0.345175 0.000366

H 2.706476 0.051845 -0.000533

O 1.081023 -1.866261 -0.000209

C 0.799755 -0.580075 0.000097

C -0.560977 -0.251477 0.000341

C -1.747912 -0.019257 0.000140

C -3.166714 0.246027 -0.000145

H -3.368788 1.319549 -0.001226

H -3.628843 -0.203963 -0.884437

H -3.628873 -0.202179 0.885038

H 2.028274 -2.075842 -0.000490

### Acrylamide CRG 3b


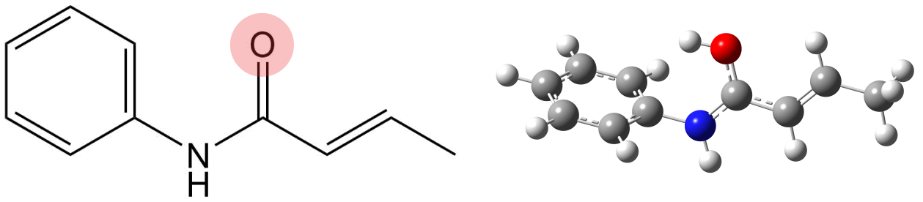


C 3.129251 -1.227296 -0.240932

C 3.874478 -0.161631 0.261339

C 3.286141 1.091283 0.435026

C 1.945742 1.286091 0.112106

C 1.209808 0.211018 -0.388547

C 1.788783 -1.046174 -0.577078

H 3.590649 -2.196487 -0.385318

H 4.917884 -0.306256 0.51432087

H 3.868948 1.918261 0.821681

H 1.478256 2.254926 0.244389

H 1.208930 -1.860055 -0.997752

N -0.180539 0.412567 -0.742672

H -0.394534 0.916493 -1.596163

C -1.201908 0.001701 -0.008460

C -2.561458 0.238382 -0.416805

H -2.700635 0.759547 -1.357922

C -3.615647 -0.157896 0.323460

H -3.422992 -0.677169 1.259072

C -5.036554 0.060332 -0.039444

H -5.158442 0.583989 -0.987547

H -5.557718 -0.902644 -0.089466

H -5.537559 0.628171 0.753116

O -0.988050 -0.631335 1.118949

H -0.036431 -0.730920 1.304691

### Acrylamide CRG 3c


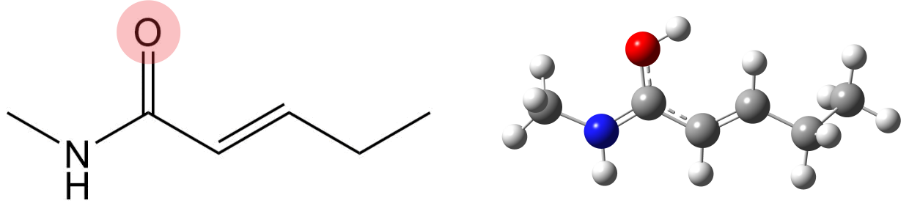


C 3.707854 0.224374 -0.552784

H 3.391302 0.129520 -1.593465

H 3.798143 1.286874 -0.313456

H 4.697879 -0.223656 -0.457356

C 2.725035 -0.488847 0.398878

H 2.675706 -1.557362 0.176798

H 3.096529 -0.387757 1.427159

C 1.362888 0.110946 0.353172

H 1.328991 1.177550 0.584553

C 0.230675 -0.554205 0.060543

H 0.264319 -1.618727 -0.146824

C -1.082882 0.052299 0.022872

N -2.163643 -0.680589 -0.103281

H -2.035178 -1.683850 -0.125139

C -3.538791 -0.159159 -0.161515

H -3.576466 0.705731 -0.821573

H -4.179848 -0.946587 -0.551081

H -3.875926 0.129088 0.835579

O -1.298670 1.347475 0.109185

H -0.479277 1.861050 0.107288

### Acrylamide CRG 3d


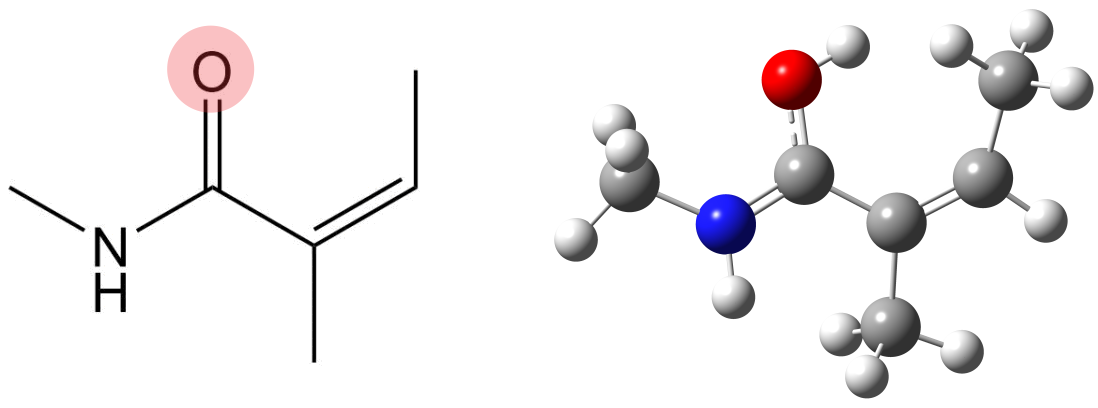


C 2.897615 -0.430535 0.192061

H 2.951724 -0.989762 -0.740361

H 2.960542 -1.121900 1.033913

H 3.717294 0.282551 0.242351

N 1.634721 0.324301 0.244588

H 1.660938 1.268746 0.605187

C 0.460409 -0.146058 -0.097398

C -0.742794 0.689151 -0.069061

O 0.467533 -1.400664 -0.497785

C -1.980818 0.164299 0.072658

H -2.789020 0.891050 0.048438

C -2.444683 -1.239977 0.304669

H -3.240136 -1.229373 1.054383

H -2.907944 -1.646071 -0.60406292

H -1.678687 -1.931159 0.659249

C -0.526964 2.185640 -0.168314

H -1.484451 2.694144 -0.272904

H -0.055681 2.593282 0.733805

H 0.091036 2.453705 -1.029191

H -0.385516 -1.665131 -0.868333

### Acrylamide CRG 3e


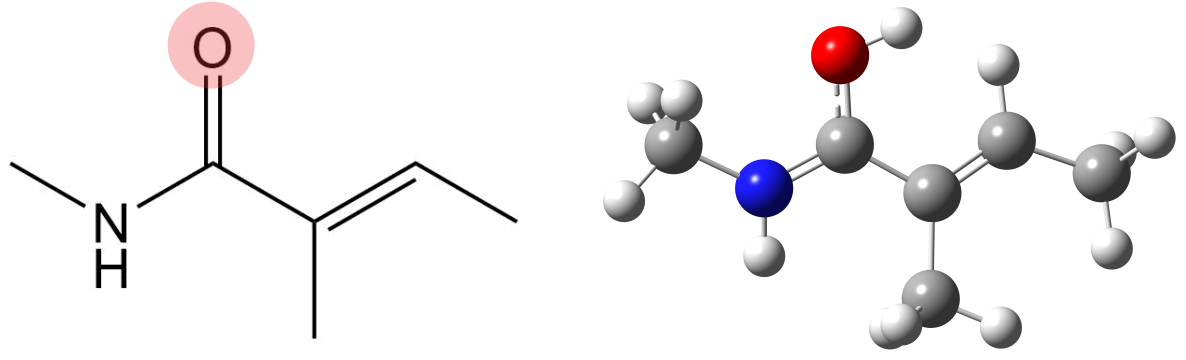


C 3.094152 0.090594 -0.156969

H 3.269819 -0.593201 0.671469

H 3.719122 0.973681 -0.044896

H 3.331371 -0.410218 -1.097003

N 1.687573 0.525135 -0.151063

H 1.490352 1.493470 -0.364859

C 0.657282 -0.258585 0.054415

C -0.714306 0.256527 0.067800

O 0.964122 -1.525007 0.248441

C -1.725812 -0.621330 -0.116195

H -1.483611 -1.669756 -0.289592

C -3.185850 -0.342874 -0.167060

H -3.577689 -0.623381 -1.151563

H -3.706884 -0.980112 0.555912

H -3.447694 0.694622 0.027349

C -0.880375 1.750039 0.222711

H -1.921640 2.012863 0.390771

H -0.308658 2.132455 1.072496

H -0.564702 2.288288 -0.67864194

H 0.203692 -2.040831 0.550267

### Acrylamide CRG 3f


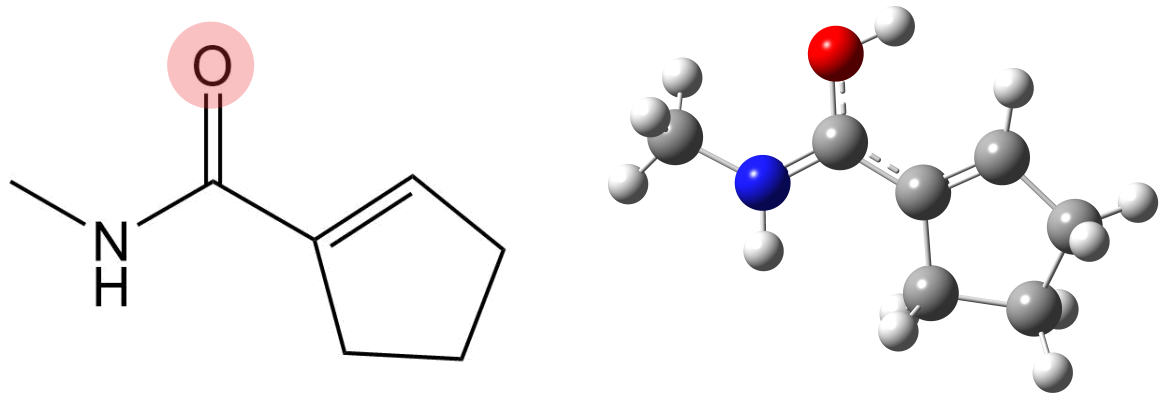


C -2.591856 -0.907593 0.229745

C -1.096104 -1.255541 0.007748

C -0.431177 0.112982 -0.001107

C -1.338262 1.103700 -0.120409

C -2.736819 0.577981 -0.184281

H -2.838474 -1.017779 1.287243

H -0.942162 -1.761228 -0.953696

H -0.710015 -1.912056 0.792539

H -1.122558 2.164433 -0.216389

H -3.112616 0.695333 -1.210060

H -3.420885 1.146565 0.451776

H -3.254532 -1.564819 -0.331473

C 1.000787 0.313366 0.030503

N 1.837541 -0.689761 -0.089084

H 1.432140 -1.602614 -0.248725

C 3.304630 -0.577792 -0.062856

H 3.713790 -1.564192 0.143690

H 3.673648 -0.221791 -1.026314

H 3.605528 0.116757 0.719238

O 1.565947 1.494814 0.181589

H 0.918565 2.188587 0.366985

### Acrylamide CRG 3g


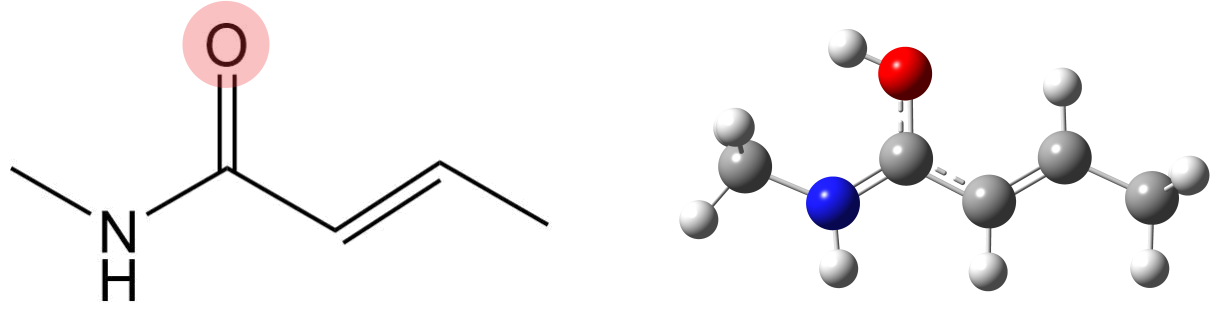


C 2.976646 -0.248028 0.000017

H 3.625291 -1.120819 -0.000186

H 3.192069 0.334910 0.899227

H 3.192045 0.335270 -0.898969

N 1.583442 -0.713338 -0.000056

H 1.420326 -1.712001 -0.000138

C 0.512552 0.057570 0.000011

C -0.811992 -0.503365 -0.000007

H -0.878846 -1.586000 -0.000033

O 0.626429 1.367380 0.000120

C -1.922040 0.262232 0.000024

H -1.804639 1.343091 0.000073

C -3.309657 -0.258004 -0.000059

H -3.848627 0.127920 0.873079

H -3.848162 0.127219 -0.873813

H -3.357637 -1.346810 0.000322

H 1.539594 1.689119 -0.00004091

### Acrylamide CRG 3h


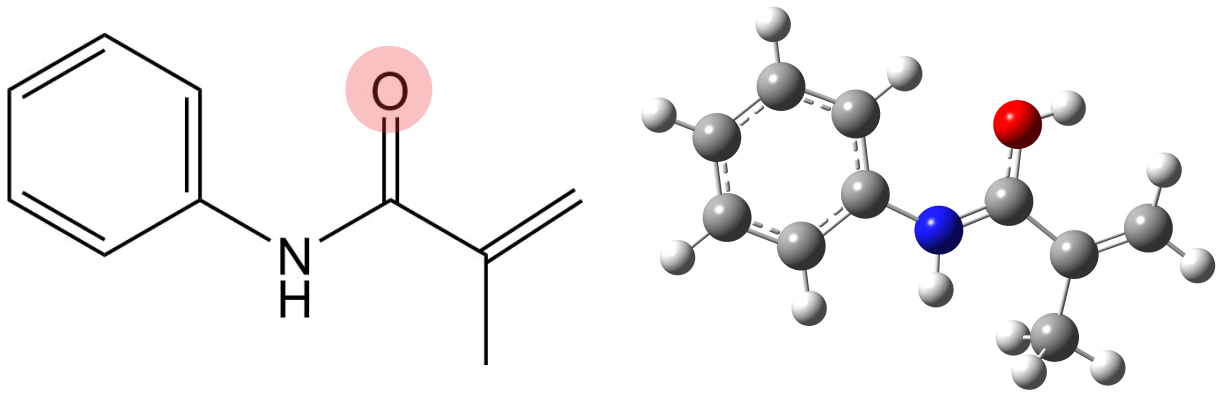


C 3.170489 -1.212992 -0.029935

C 3.764524 0.048404 0.009448

C 2.968661 1.192358 0.014735

C 1.580296 1.099651 -0.015531

C 0.995017 -0.170520 -0.047024

C 1.788007 -1.325693 -0.060736

H 3.781230 -2.107147 -0.037474

H 4.843638 0.138587 0.032023

H 3.428710 2.172572 0.039078

H 0.982484 1.996304 -0.01905293

H 1.326808 -2.308184 -0.090619

N -0.415296 -0.405921 -0.083740

H -0.652415 -1.381226 -0.226794

C -1.458998 0.382660 0.035627

C -2.822053 -0.172650 -0.012715

C -3.851627 0.648998 -0.266365

H -3.745291 1.707160 -0.483836

O -1.228156 1.668308 0.194493

H -4.865454 0.270195 -0.301452

C -2.995586 -1.657463 0.198605

H -4.054849 -1.908955 0.222301

H -2.554154 -2.240489 -0.618187

H -2.551937 -1.988710 1.141723

H -2.038843 2.148358 0.415881

### Acrylamide CRG 3i


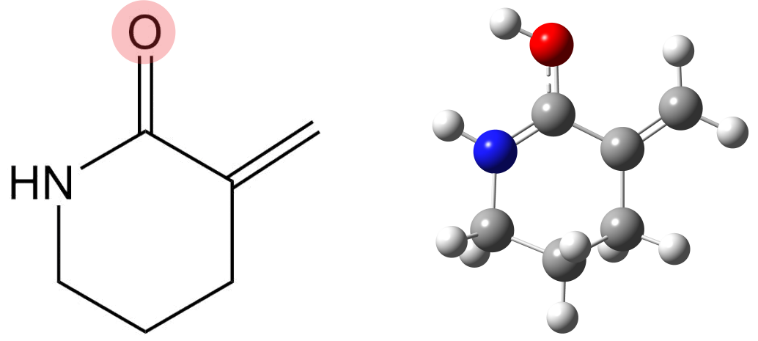


C -0.698065 1.441995 0.292558

C -1.809439 0.640512 -0.393883

C -1.810337 -0.797434 0.107224

H -0.672733 2.466493 -0.079909

H -1.671198 0.654876 -1.479128

H -2.439547 -1.442149 -0.507416

H -0.896077 1.500739 1.369447

H -2.786591 1.080073 -0.186779

H -2.157571 -0.864990 1.141976

C 0.652077 0.794599 0.068913

C 1.804112 1.464542 -0.088560

H 1.814597 2.548205 -0.072027

H 2.751305 0.961709 -0.234743

C 0.673122 -0.665013 0.025118

O 1.849442 -1.251618 -0.045647

N -0.436476 -1.367283 0.057789

H -0.371768 -2.380072 0.071054

H 1.820560 -2.216168 -0.130034

### Acrylamide CRG 4a


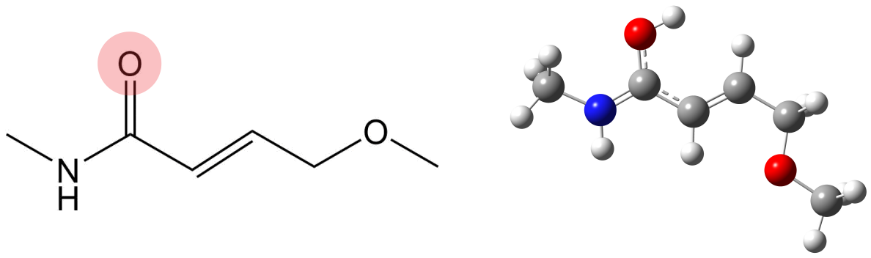


C -2.260620 -0.791823 -0.101752

H -2.694044 -1.407493 0.707168

H -2.662252 -1.205726 -1.044034

O -2.567661 0.561840 0.049359

C -3.972030 0.837172 0.052662

H -4.076226 1.913169 0.174959

H -4.468070 0.326398 0.885082

H -4.431909 0.531378 -0.893306

C -0.785616 -0.992515 -0.096051

H -0.471001 -2.027280 -0.223918

C 1.528427 -0.153176 0.034197

O 2.135784 -1.319331 0.105744

C 0.092387 0.014823 0.035169

H -0.284821 1.027096 0.125925

N 2.325710 0.885174 -0.042367

H 1.882965 1.788778 -0.151110

C 3.795850 0.832953 -0.039882

H 4.134437 0.079386 0.668416

H 4.166834 1.811173 0.258043

H 4.166630 0.588105 -1.036912

H 1.522117 -2.045866 0.279375

### Acrylamide CRG 4b


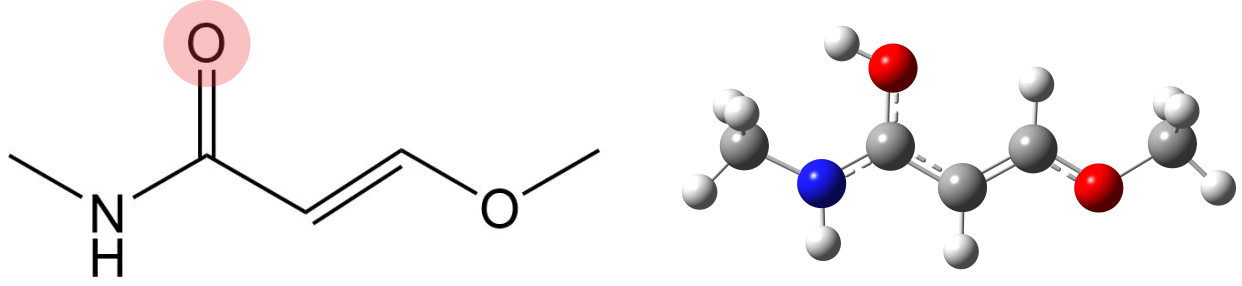


C -3.760171 0.242162 0.000002

H -4.575396 -0.475094 0.000252

H -3.796829 0.856299 -0.900491

H -3.796647 0.856720 0.900216

O -2.544206 -0.555949 0.000054

C -1.395555 0.063013 0.000015

H -1.403719 1.152013 -0.000006

C -0.226716 -0.640412 0.000037

H -0.252374 -1.722454 0.000065

C 1.030236 0.010973 0.000012

N 2.170483 -0.667100 -0.000062

H 2.097151 -1.675309 -0.000169

C 3.514212 -0.079042 -0.000091

H 3.681361 0.520684 0.898698

H 3.681457 0.520417 -0.899041

H 4.238205 -0.890630 0.000078

O 1.047343 1.334839 0.000079

H 1.936279 1.715774 -0.000095

### Acrylamide CRG 4c (deprotonated)


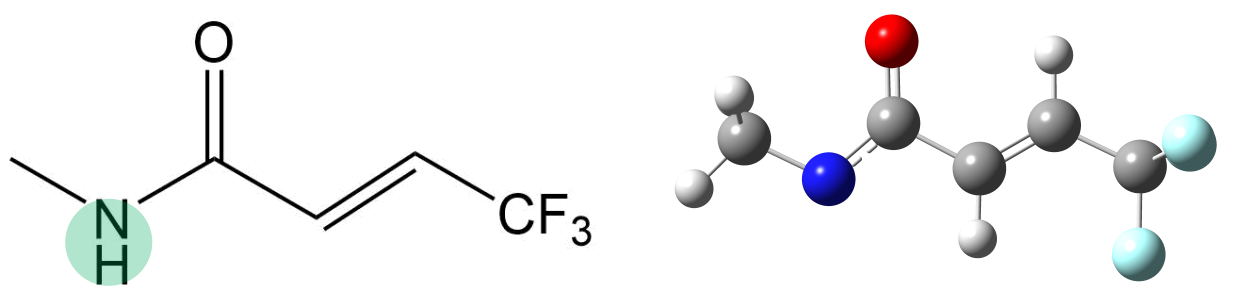


N -2.705775 0.805180 -0.000163

C -1.815799 -0.175654 -0.000480

C -0.409697 0.357740 -0.000176

H -0.304391 1.437306 -0.000125

C 0.657865 -0.446353 -0.000090

H 0.519747 -1.522994 -0.000166

O -1.997584 -1.426123 0.000026

C -4.079300 0.341925 0.000297

H -4.314699 -0.283847 -0.877253

H -4.314207 -0.283610 0.878150

H -4.756151 1.204758 0.000385

C 2.055415 0.023386 0.000043

F 2.201730 1.372508 0.000374

F 2.767987 -0.430145 1.084766

F 2.768048 -0.429603 -1.084876
